# Design, Synthesis, and Evaluation of a New Fluorescent Ligand for the M_2_ Muscarinic Acetylcholine Receptor

**DOI:** 10.1021/acsmedchemlett.4c00592

**Published:** 2025-03-20

**Authors:** Renáta Szabó, Dénes Szepesi Kovács, Dóra Judit Kiss, Zeinab Nezafat Yazdi, András Dávid Tóth, Jose Brea, María Isabel Loza, Domokos Meszéna, Lucia Wittner, István Ulbert, Balázs Volk, László Hunyady, György Miklós Keserű

**Affiliations:** † Medicinal Chemistry Research Group, 579839 HUN-REN Research Centre for Natural Sciences, H-1117 Budapest, Hungary; ‡ Department of Organic Chemistry and Technology, Budapest University of Technology and Economics, H-1111 Budapest, Hungary; § National Laboratory for Drug Research and Development, H-1117 Budapest, Hungary; ⊥ Institute of Molecular Life Sciences, Centre of Excellence of the Hungarian Academy of Sciences, HUN-REN Research Centre for Natural Sciences, H-1117 Budapest, Hungary; ¶ Department of Internal Medicine and Haematology, Semmelweis University, H-1088 Budapest, Hungary; ∥ Innopharma Drug Screening and Pharmacogenomics Platform, BioFarma Research Group, Center for Research in Molecular Medicine and Chronic Diseases (CiMUS), Department of Pharmacology, Pharmacy, and Pharmaceutical Technology, 16780 University of Santiago de Compostela, 15705 Santiago de Compostela, Spain; ○ Department of Neurology, Center for Neurotechnology and Neurorecovery, Massachusetts General Hospital, Harvard Medical School, Boston, Massachusetts 02114, United States; ◆ Integrative Neuroscience Research Group, Institute of Cognitive Neuroscience and Psychology, HUN-REN Research Centre for Natural Sciences, H-1117 Budapest, Hungary; ∇ Department of Neurosurgery and Neurointervention, Semmelweis University, H-1145 Budapest, Hungary; ⬣ Department of Information Technology and Bionics, Péter Pázmány Catholic University, H-1083 Budapest, Hungary; ⬒ Directorate of Drug Substance Development, Egis Pharmaceuticals Plc., P.O. Box 100, H-1475 Budapest, Hungary; ◮ Department of Physiology, Faculty of Medicine, Semmelweis University, H-1094 Budapest, Hungary

**Keywords:** fluorescent probe, G protein-coupled receptor, imaging, two-photon microscopy, STED nanoscopy

## Abstract

The M_2_ muscarinic acetylcholine receptor (M_2_R) is a G protein-coupled receptor involved in regulating cardiovascular functions and mediation of central muscarinic effects, such as movement, temperature control, and antinociceptive responses. Molecular probes targeting this receptor are therefore important in exploring its pathophysiological role at a molecular level. Herein, we report the design, synthesis, and evaluation of a new fluorescent probe for M_2_R based on an anthranilamide ligand. In radioligand binding experiments, the presented Oregon Green 488-labeled conjugate (**33**) exhibited high M_2_R affinity (*K*_i_ = 2.4 nM), a moderate preference for the M_2_R over the M_4_ receptor, and excellent to pronounced M_2_R selectivity compared to the M_1_, M_3_, and M_5_ receptors. The utility of the probe was demonstrated in confocal, two-photon, and stimulated emission depletion nanoscopy (STED) imaging to specifically label the receptors in human embryonic kidney (HEK) 293T cells. These properties suggest that our probe may be utilized in advanced microscopy to study the pharmacology of the M_2_R.

## Graphical abstract

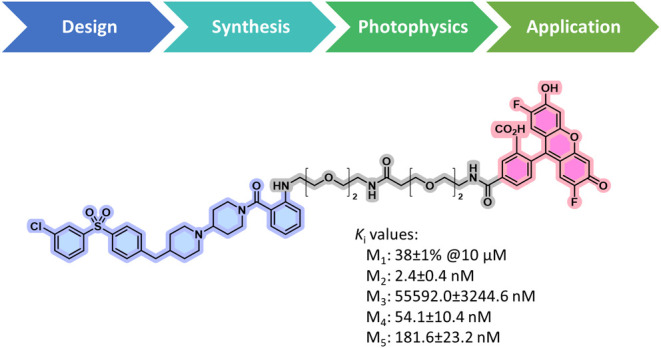

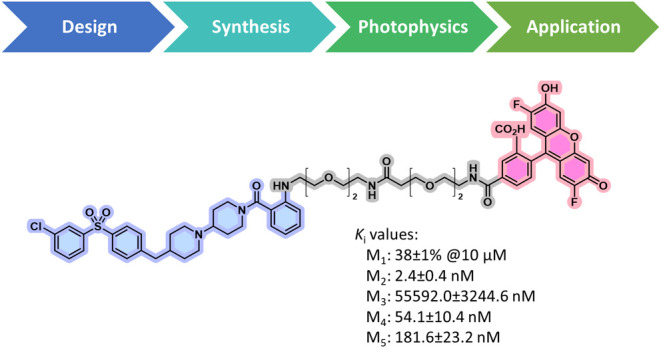

Muscarinic acetylcholine receptors (mAChRs) are G protein-coupled receptors (GPCRs) with five subtypes (M_1_–M_5_) divided into two functional classes based on G protein coupling: M_1_, M_3_, and M_5_ with G_q_ and M_2_ and M_4_ with G_i_/G_o_.^1^ Activation of mAChRs by agonists like acetylcholine (ACh) triggers biochemical and electrophysiological responses, depending on the receptor subtype and location. Involved in both central and parasympathetic nervous systems,^2^ muscarinic receptors are therapeutic targets for conditions like Alzheimer’s disease (AD), addiction, epilepsy, schizophrenia, and Parkinson’s disease.^3–5^

Designing subtype-selective mAChR ligands remains a significant challenge for medicinal chemists. Early efforts focused on compounds with a pyridobenzodiazepinone core (**1**, **2**), but their selectivity versus other muscarinic receptor subtypes is modest (Figure 1).^6,7^ The discovery of potent M_2_R antagonists led to the synthesis of piperidine analogues, including **3**, a highly potent and selective antagonist with poor pharmacokinetics.^5,8^ Further optimization focused on reducing clearance rates and enhancing selectivity, leading to antagonist **4**, which replaces the (2-methyl)­benzoyl group of **3** with an anthranilic acid amide. This compound (**4**) shows high M_2_R selectivity, good oral bioavailability, and *in vivo* efficacy in rats.^9^

**Figure 1:**
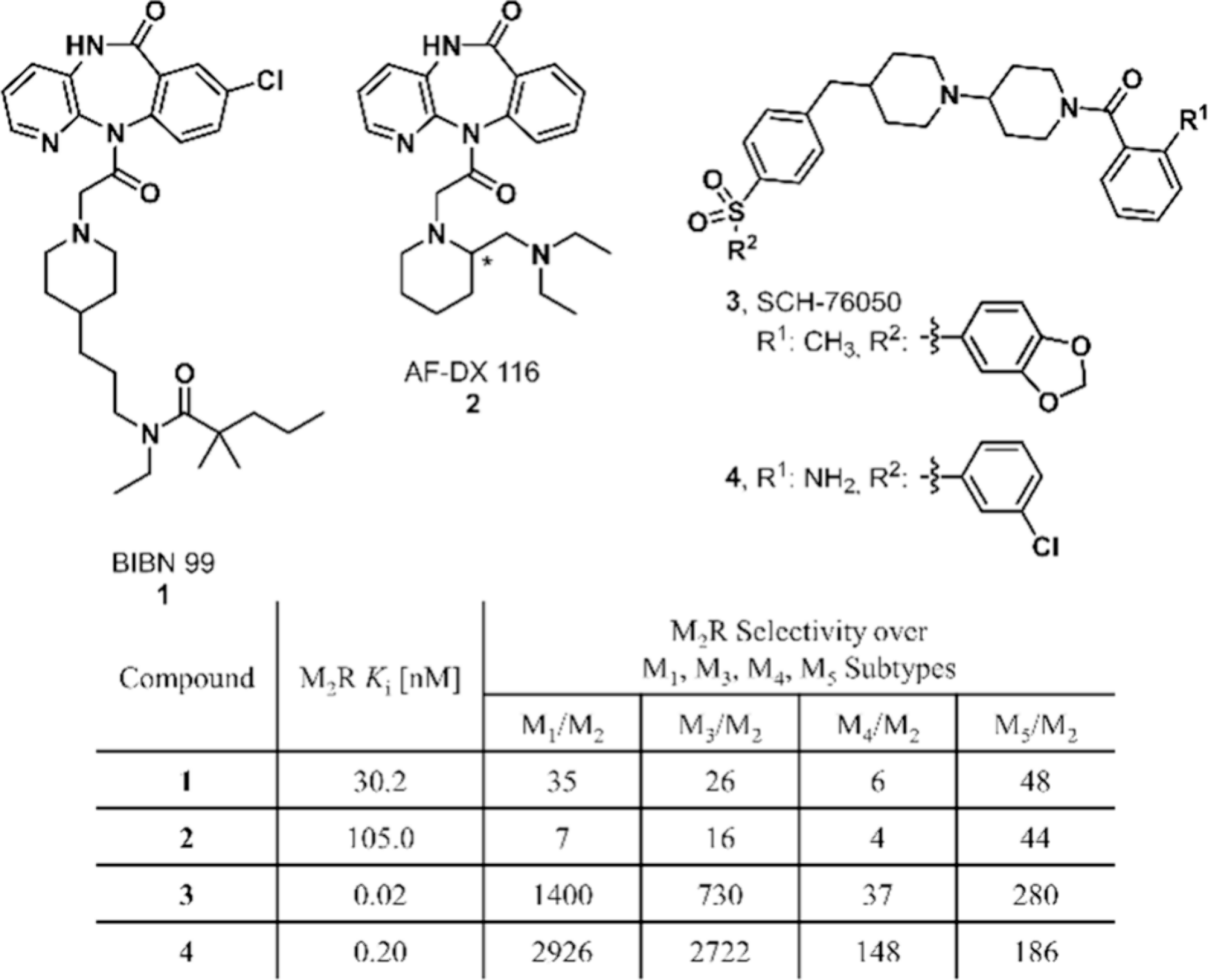
Structural formulas, M_2_R affinities, and M_2_R selectivity over M_1_, M_3_, M_4_, and M_5_ subtypes of M_2_R antagonists.^6–9^

Various fluorescent probes for muscarinic receptors prepared by the conjugation of a ligand with a fluorophore have been reported in the literature. These fluorescent ligands are based on derivatives preferring the M_1_ subtype, e.g., pirenzepine,^10–12^ telenzepine,^13,14,12^ and AC-42,^15^ and those preferring the M_3_ subtype, e.g., tolterodine.^16^ Few studies used ligands (structurally closely related to pyridobenzodiazepinones shown in Figure 1) with various fluorescent dyes having high affinity but moderate selectivity for M_2_R (Figure 2). These probes exhibit potential for use in imaging and binding studies with evidence suggesting a dualsteric mode of action.^17–20^ Unfortunately, despite all efforts, these fluorescent M_2_R ligands lack selectivity toward the M_1_ and M_4_ receptors.^21^

**Figure 2:**
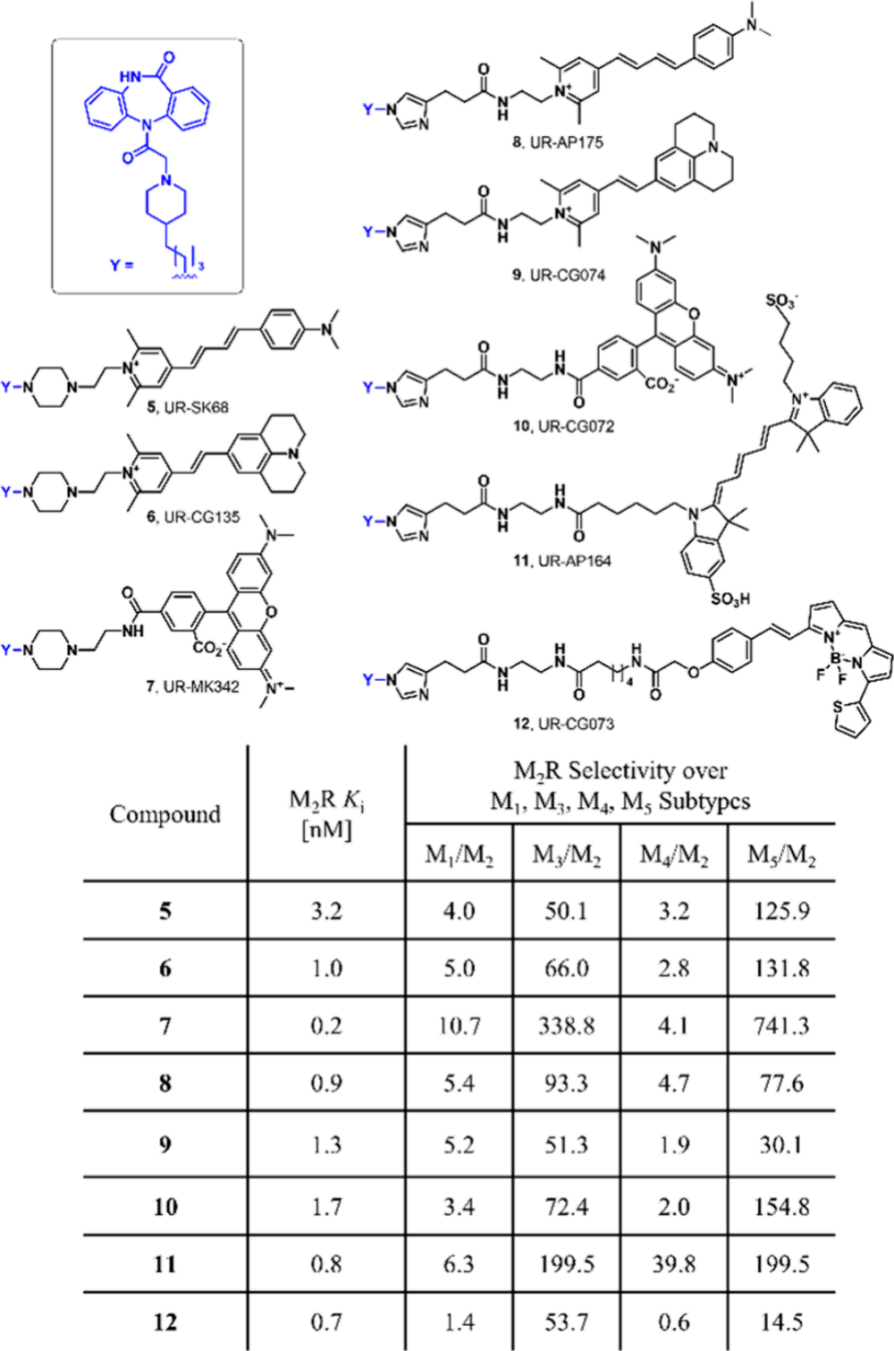
Structures, M_2_R binding data, and M_2_R selectivity over M_1_, M_3_, M_4_, and M_5_ subtypes of dibenzodiazepinone-type fluorescent ligands conjugated with various fluorescent dyes reported in the literature.^17,20^

In recent years, modern high-resolution imaging techniques have become useful and common tools for visualization and dynamic monitoring.^22^ Confocal microscopy is a widely used technique for studying the labeling of proteins with fluorescent probes^23–27^ while there are a number of examples of two-photon microscopy^28,29^ and stimulated emission depletion nanoscopy (STED) reported in the literature.^30–32^ Two-photon and STED imaging techniques offer efficient visualization with significant advantages over confocal microscopy for three-dimensional fluorescence imaging.^32^ These benefits include enhanced 3D resolution and a minimized photodamage.

In the present study, we report a new fluorescently labeled anthranilamide-type M_2_R ligand using Oregon Green 488 (OG488) fluorescent dye to improve the selectivity and to broaden the scope of the application compared to existing probes. The OG488 fluorophore was chosen because it has a high extinction coefficient and fluorescence quantum yield, and it is suitable for both two-photon microscopy and STED imaging.

## Results and Discussion

While searching for selective M_2_ antagonists in the literature, we selected compound **4**.^9^ Based on the published data obtained by radioligand displacement assays, **4** shows high affinity (*K*_i_ = 0.2 nM) on the M_2_ receptor and exhibits remarkable selectivity toward most muscarinic receptor subtypes with values of 2926, 2722, 148, and 186 against M_1_, M_3_, M_4_, and M_5_, respectively (Figure 1). As the experimental binding mode of this compound is unknown, computational tools have been used to predict its binding pose and to design the appropriate attachment point and length of the linker. For these structure-based calculations, we started from the crystal structure of the receptor available in the Protein Data Bank (PDB: 5ZKB).^33^ As our selected ligand core differs from the ligand in the experimental structure, induced fit docking calculations have been run that allow the movement and adaptation of the side chains in the binding site in order to determine the binding mode of the ligand (Figure 3). Several plausible binding poses have been obtained; therefore, we have further assessed their stability with binding pose metadynamics. In the most plausible binding mode (Figure 3a), the ligand is stabilized in a dualsteric binding mode occupying the orthosteric pocket and also extending toward the extracellular vestibule interacting with residues on the top part of TM2,3,7 and ECL2. The molecule is anchored to the conserved Asp103^3.32^ by a hydrogen bond and further stabilized with two additional hydrogen bonds with Asn404^6.52^ and Tyr426^7.39^. Linker attachment on the free amine group was chosen as the best exit vector since it is positioned toward the extracellular side and allows the elongation of the ligand without installing additional substituents into the core. Docking calculations into the grid prepared based on the ligand-bound structure obtained from IFD showed that two PEG_3_ linkers positioned the attachment of the Oregon Green 488 dye well outside the extracellular side of the receptor, therefore allowing the ligand core of the conjugate to occupy the same binding pose as the original small ligand.

**Figure 3:**
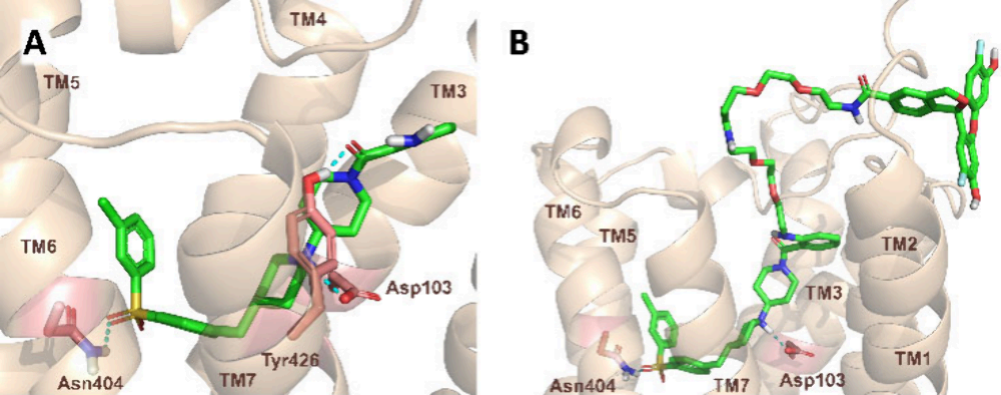
Design of the fluorescent probe. (A) The predicted binding mode of the base compound (**28**) and (B) the fluorescently labeled molecule (**33**). The receptor is represented as a wheat cartoon and the ligand, as green licorice; the hydrogen bonds are indicated with cyan dashed lines.

Based on the information obtained from the molecular docking studies, a novel fluorescent antagonist for the M_2_ receptor was synthesized featuring an OG488-based probe. The synthetic route to the desired probe is illustrated in Schemes 1–3.

**Scheme 1:**
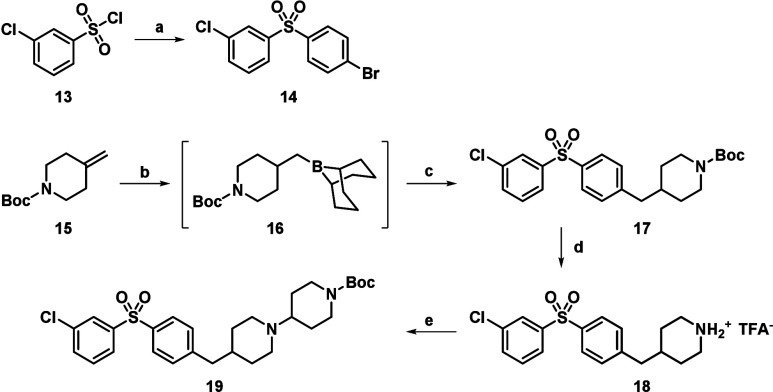
Synthesis of the Sulfone Core^a^

a Reagents and conditions: (a) bromobenzene, AlCl_3_, 80 °C, 1 h, 81%; (b) 9-BBN, 65 °C, 1 h; (c) **14**, PdCl_2_(dppf)·CH_2_Cl_2_, K_2_CO_3_, DMF:water (10:1), 60 °C, 3 h, 83%; (d) TFA, DCM, 25 °C, 2 h, 93%; (e) 1-Boc-4-bromopiperidine, K_2_CO_3_, KI, MeCN, 80 °C, 24 h, 78%.

For the first step, we developed an efficient solvent- and chromatography-free synthesis of sulfone **14** (Scheme 1). We have found that bromobenzene could be acylated with 3-chlorobenzenesulfonyl chloride (**13**) in the presence of aluminum chloride (AlCl_3_) at 80 °C to afford the expected sulfone (**14**) in excellent yield. Then, we followed the literature procedure^9^ for the synthesis of intermediate **17**. Boc-protected methylidene piperidine **15** was treated with 9-BBN to form a borane intermediate (**16**), which was then coupled with bromoarylsulfone **14** in the presence of a palladium catalyst to produce compound **17**. The Boc group was removed, and the corresponding amine was generated as its TFA salt (**18**). Then, N-alkylation with 1-Boc-4-bromopiperidine followed to give **19**.

Subsequently, compound **25** was prepared using the available literature protocols (Scheme 2).^34,35^ The synthesis of the final intermediates containing the PEG functionalized tail was started by selective protection of one amino group of commercially available **22** with Boc_2_O in DCM. The resulting Boc-protected amine was coupled with 2-iodobenzoic acid under copper catalysis at room temperature to give **23**. The next step was the removal of the Boc functional group under acidic conditions to generate the corresponding amine as its TFA salt (**24**). The carboxylic acid group of compound **20** was activated with *N*-hydroxysuccinimide to afford **21**. Then, the amine (**24**) was N-acylated with NHS-ester-activated compound (**21**) to give compound **25**.

**Scheme 2:**
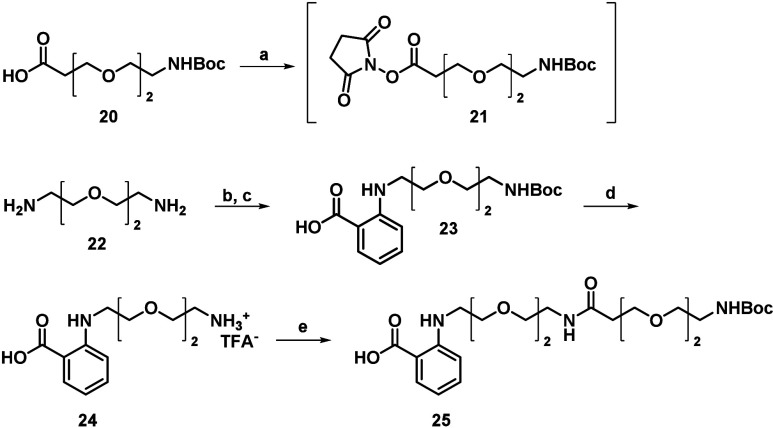
Synthesis of the PEG Linker Containing Derivatives^a^

a Reagents and conditions: (a) NHS, DCC, THF, 25 °C, 2 h; (b) Boc_2_O, DCM, 0 °C → 25 °C, 24 h; (c) 2-iodobenzoic acid, CuI, rac-BINOL, K_3_PO_4_, DMF, 25 °C, 3 h, 69%; (d) TFA, DCM, 25 °C, 2 h, 93%; (e) **21**, TEA, 25 °C, 0.5 h, 60%.

The Boc group in **19** was removed, and the resulting piperidine (**26**) was coupled with various carboxylic acids (Boc-2-Abz-OH, **23**, **25**) under standard conditions (Scheme 3). The Boc protecting group was then removed, and three different amine intermediates were obtained (**28**, **30**, **32**). Finally, **32** was coupled to the appropriate commercially available OG488 succinimidyl esters. The novel fluorescent conjugate **33** was isolated and purified by preparative HPLC. Additionally, the chemical identity of new compounds and that of the probe was confirmed by NMR and high-resolution mass spectrometry (HRMS).

**Scheme 3:**
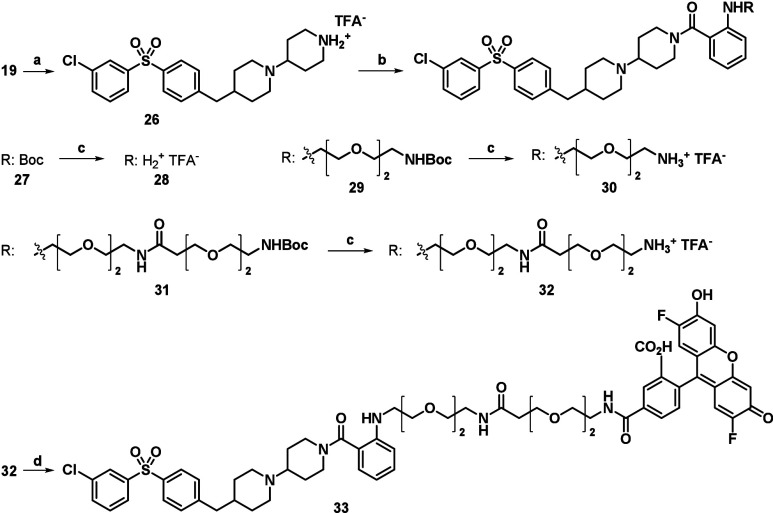
Synthesis of the PEG-Containing Intermediates and the Novel Fluorescent M_2_R Antagonist **33** ^a^

a Reagents and conditions: (a) TFA, DCM, 25 °C, 2 h, 95%; (b) Boc-2-Abz-OH or **23** or **25**, HOBt·H_2_O, EDC·HCl, TEA, DMF, 25 °C, 2 h, 55% (**27**); 50% (**29**); 48% (**31**); (c) TFA, DCM, 25 °C, 2 h, 95% (**28**); 92% (**30**); 97% (**32**); (d) OG488-NHS, DIPEA, DMF, 25 °C, 0.5 h, 40%.

Next, we investigated the photophysical properties of the fluorescent probe **33**. In acetonitrile, the probe has an absorption maximum at 520 nm and a fluorescence maximum at 547 nm with a Stokes shift of 27 nm. The molar extinction coefficient (ε = 42504.9 M^–1^ cm^–1^), fluorescence quantum yield (Φ_f_ = 0.20), and brightness (*f* = 8501) values are lower than those of the original dye^36^ but are considered adequate for future biological applications. Figure S1 shows the absorbance and fluorescence spectra of probe **33** in various solvents, including DCM, dioxane, ethanol, ethyl acetate, phosphate buffered saline (PBS), THF, toluene, water, and acetonitrile. Higher solvent polarity correlated with increased fluorescence intensity, while aqueous media showed a lower intensity. The dye was pH sensitive, with intensity increasing at higher pH. Following a literature procedure, we examined the effect of protein (BSA) on the ligand.^20^ We found that the absorbance, excitation, and emission maxima show no significant change in PBS with and without BSA, as shown in Figure S1. Finally, to prove the ability for biological application, we have investigated the photostability of **33** in PBS (pH 7) excited with UV light (4 W, 450 nm). Half of the original fluorescence intensity was reached after 10 min of irradiation, which is still considered appropriate because a typical imaging process does not require longer continuous excitation.^20^

The M_1_R–M_5_R affinities of the intermediates **28**, **30**, **32** and the fluorescently labeled derivative **33** were determined at membranes from CHO cells transfected with hM_*x*_R (*x* = 1–5) using the orthosteric antagonist [^3^H]-scopolamine (*N*-methyl) as a radioligand (for competition binding curves, see Figure S2). Before testing the fluorescent probe (**33**), we checked for the binding affinity and selectivity of the base derivative (**28**) and the PEG linker-containing derivatives (**30**, **32**). The corresponding *K*_i_ values are listed in Table 1.

**Table 1:** Results of Binding Affinity of Compounds **28**, **30**, **32**, and **33** Tested^a^

	Receptor *K*_i_ [nM]				
Compounds	M_1_	M_2_	M_3_	M_4_	M_5_
**28**	425 ± 32	1.1 ± 0.2	193 ± 23	9.4 ± 1.1	119.0 ± 20.0
**30**	143 ± 11	1.5 ± 0.3	496 ± 36	7.4 ± 0.6	8.6 ± 0.7
**32**	113 ± 8	1.1 ± 0.1	373 ± 38	6.2 ± 0.9	8.5 ± 0.9
**33**	n.d.^a^	2.4 ± 0.4	55592 ± 3245	54.1 ± 10.4	182 ± 23

a Results are the mean ± SD of three independent experiments (*n* = 3).

b *K*_i_ value could not be determined because inhibition at 10 μM was only 38 ± 1%.

We observed that the initial molecule (**28**) exhibited lower selectivity for the M_4_ receptor than previously reported in the literature.^9^ This discrepancy is likely due to differences in the assay conditions used during our testing.

Interestingly, the application of the dye enhances the selectivity of the molecule across all four additional receptors (M_1_, M_3–5_). The new fluorescent ligand **33** we developed is a more selective M_2_R probe compared to the dibenzodiazepinone-type fluorescent compounds reported in the literature.^20,17^ Its selectivity for the M_1_ and M_3_ receptors is particularly notable, while its selectivity for the M_4_ and M_5_ receptors is comparable to those of previously published compounds (see Figure 2).

The ability of the compounds to antagonize the carbachol-induced G protein activation of M_2_R was assessed using the bioluminescence resonance energy transfer (BRET)-based TRUPATH G_oA_ activation assay (Figure 4A,B).^37^ All synthesized compounds (**28**, **30**, **32**, and **33**) acted as competitive antagonists, displaying similar half-inhibitory concentration (IC_50_) values (Table 2).

**Table 2:** IC_50_ Values for Carbachol-Induced G_oA_ Protein Activation^a^

Compound	Log(IC_50_) ± SD (M)
**28**	–7.10 ± 0.23
**30**	–6.88 ± 0.13
**32**	–6.97 ± 0.12
**33**	–7.39 ± 0.09

a TRUPATH GoA biosensor-expressing HEK 293T cells were treated with the indicated compounds at increasing concentrations for 20 min, followed by stimulation with 10 μM carbachol for 15 min, as shown in Figure 4A,B. Concentration–response curves were fitted with GraphPad Prism 9 software (Hill-slope = 1, *n* = 3).

**Figure 4:**
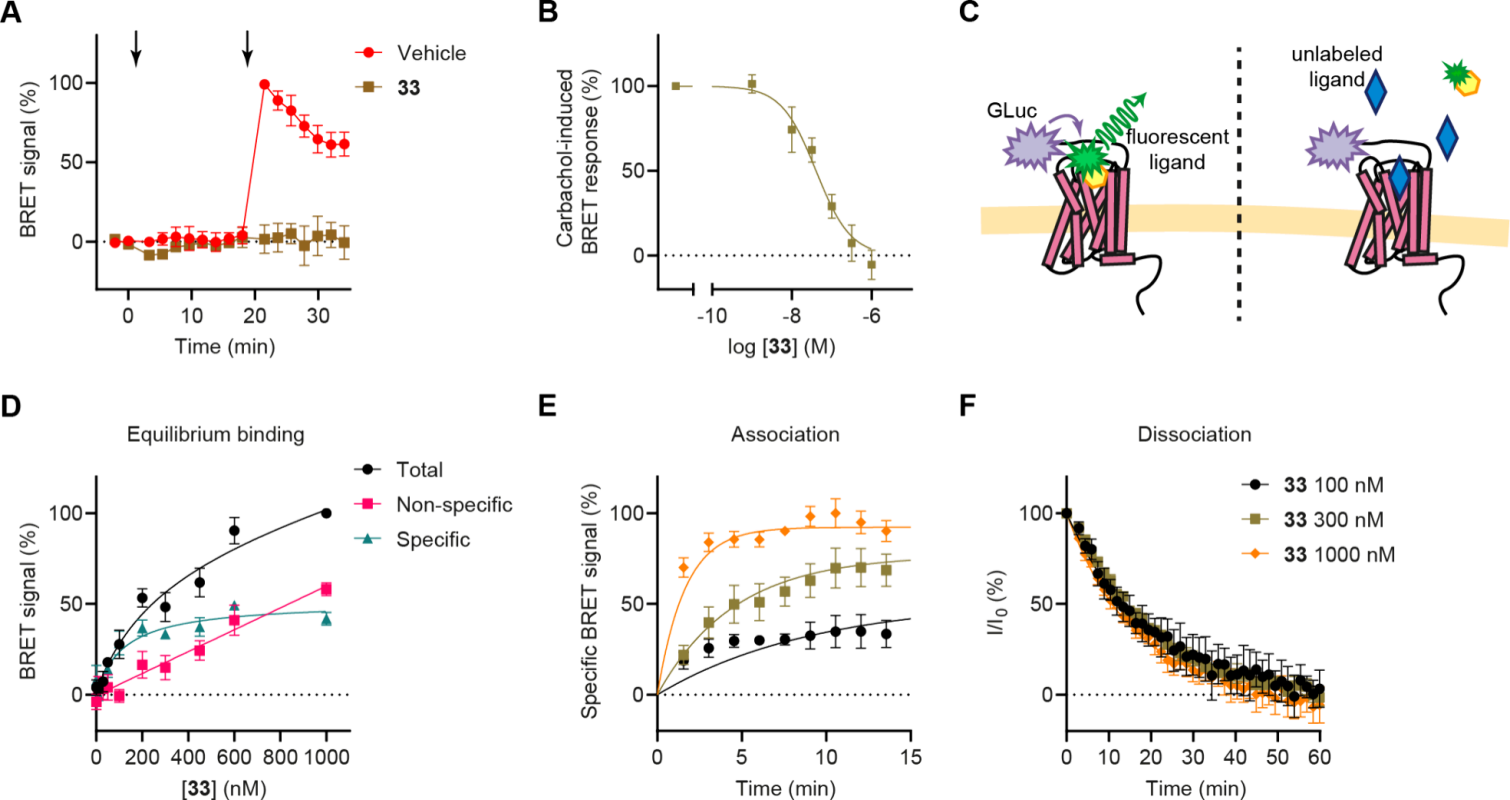
Functional activity and BRET-based ligand binding results. (A) Kinetic measurement of M_2_R activity in the TRUPATH G_oA_ assay. The first arrow indicates the time of treatment with either vehicle or 300 nM compound **33**, while the second arrow marks the application of 10 μM carbachol. (B) Concentration–response analysis of the antagonistic effect of compound **33** on 10 μM carbachol-induced G_oA_ activation in the TRUPATH assay, and the IC_50_ value is shown in Table 2. (C) Schematic representation of the BRET-based ligand binding assay. (D) Equilibrium BRET-based ligand binding data. Nonspecific signals were determined using a 5 min pretreatment with 100 μM atropine. (E) Kinetic measurement of **33** binding to assess association kinetics. Data from a representative measurement performed in triplicate are shown and represented as mean ± SD. The experiment was repeated three times with consistent results. The “Association kinetics–Two or more conc. of hot.” equation (GraphPad Prism software) was fitted to the data points (*R* ^2^ = 0.83). (F) Monitoring the dissociation kinetics of compound **33**. Compound **33** was displaced from the receptor by the application of 100 μM atropine. The “One phase decay” equation was fitted to the data points (*R* ^2^ = 0.88). Data in (A), (B), (D), and (F) are presented as the mean ± SD of three independent biological replicates.

The suitability of the fluorescent compound for BRET-based ligand binding measurements was also evaluated (Figure 4). In these experiments, binding of the fluorescent receptor ligand to the BRET donor-fused receptor construct ensures a molecular proximity, resulting in resonance energy transfer and an increase in the BRET ratio (Figure 4C).^38,39^ The specificity of the signal can be verified by treatment with nonlabeled competitive receptor ligands, which prevents the BRET signal. To generate the BRET donor labeled receptor construct, we fused the bright *Gaussia* luciferase enzyme^39^ to the N-terminus of M_2_R. In our measurements, a concentration-dependent and specific increase in the BRET signal was observed after treatment with **33**, yielding a dissociation constant (*K*_D_) of 119 nM (Figure 4D). This value is higher than the *K*_i_ value obtained from the radioligand binding assay, likely due to differences in the experimental conditions and the receptor constructs used. The amplitude of the specific BRET ratio change was relatively low, potentially reflecting a suboptimal orientation of the BRET partners, which may have limited the efficiency of the resonance energy transfer. However, the low variability in the signal ensured precise and reliable measurements. Competitive ligand binding measurements were performed using unlabeled M_2_R ligands, specifically atropine and compound **28** (Figure S3). This assay also proved to be effective for determining kinetic ligand binding parameters (Figure 4E,F). The calculated association and dissociation rate constants were 8.06 × 10^–3^ s^–1^ M^–1^ and 8.67 × 10^–4^ s^–1^, respectively.

Having validated **33** as a selective and useful fluorescent probe for M_2_R pharmacological investigations, we next examined the potential for visualization of M_2_R in live transfected HEK 293T cells by confocal microscopy. HEK 293T cells were transfected with Cerulean-labeled M_2_ receptors, and nontransfected cells were used as controls. The images revealed significant labeling in M_2_R–Cerulean-expressing cells (Figure 5A) after addition of **33**, whereas no labeling was observed in nontransfected cells (Figure 4B). The signal of **33** was not detected on the control cells during the examined time frame (3 min), but on the transfected cells, it remained unchanged. For additional confocal images, see Figures S4, S5, and S9. To evaluate the specificity of the binding, the receptors pretreated with atropine show no labeling with ligand **33**, as shown in Figure S6.

**Figure 5:**
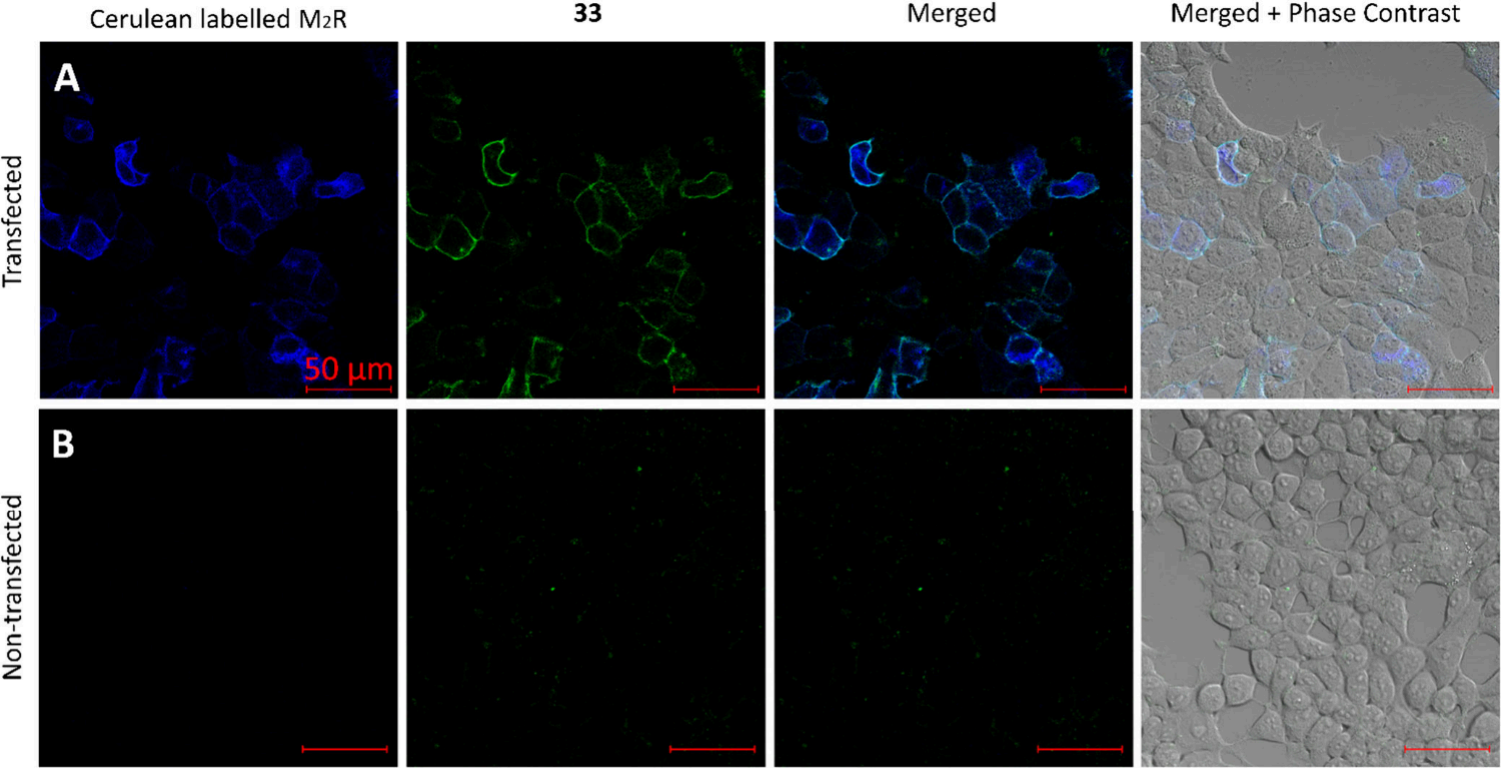
Visualization of **33** (300 nM) labeling of live M_2_R–Cerulean-expressing HEK 293T cells using confocal microscopy. (A, B) Images obtained after 3 min from M_2_R–Cerulean-transfected (A) and nontransfected (B) cells treated with **33** (300 nM). Blue represents the Cerulean fluorescence, and the fluorescent signal of **33** is shown in green. Scale bars: 50 μm. Representative images of two independent experiments are shown.

Confocal microscopy results confirmed the selective labeling of **33** against M_2_ receptors. Based on the encouraging results, we have evaluated its potential in two-photon and higher-resolution imaging techniques.

We examined live M_2_R cells with two-photon microscopy as well (Figure 6). A labeling pattern similar to that observed in the confocal microscopy studies was identified. Figure 6A illustrates **33** labeling in HA–M_2_R-transfected cells. The labeling can be observed selectively only at the plasma membrane of cells where the receptors can be found. Similarly to the confocal images, no dye labeling was detected inside the cells. Figure 6B presents a microscopic image of nontransfected cells, showing a minor degree of autofluorescence. When the probe was applied to these nontransfected cells, no receptor labeling was observed. This investigation confirmed the usefulness of the fluorescent probe in two-photon microscopy and demonstrated the labeling of the M_2_ receptors in living cells. For additional two-photon images, see Figures S7 and S8.

**Figure 6:**
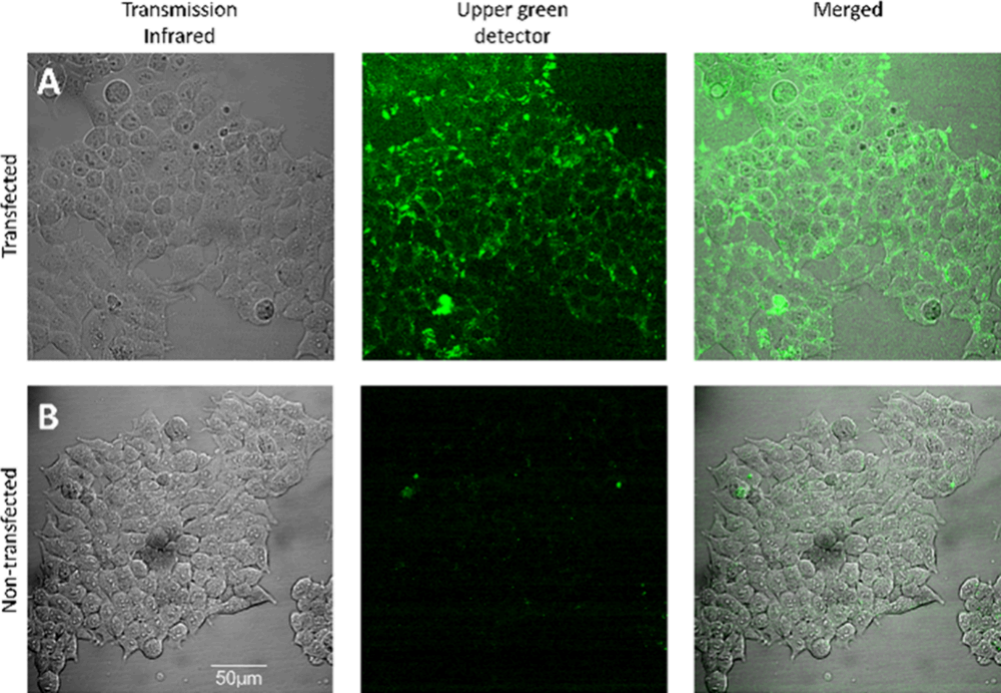
Two-photon microscopy imaging of M_2_R using **33**. (A) **33** (300 nM) labeling of live HA–M_2_R-transfected HEK 293T cells excited at 820 nm. (B) Images obtained from nontransfected HEK 293T cells. The green outlines show that **33** bound to the M_2_ receptor in the plasma membrane. Scale bar: 50 μm. Representative images of two independent experiments are shown.

As STED microscopy is used for the deeper investigation of cellular processes even at the subcellular level, we challenged the fluorescent probe **33** in STED nanoscopy (Figure 7). For STED, we used the same transfected cells as in the case of two-photon microscopy. Using the STED function and the available largest magnification objective, we observed improvement of optical resolution. The **33** green signal is concentrated in the membranes of cells, where the M_2_Rs are located.

**Figure 7:**
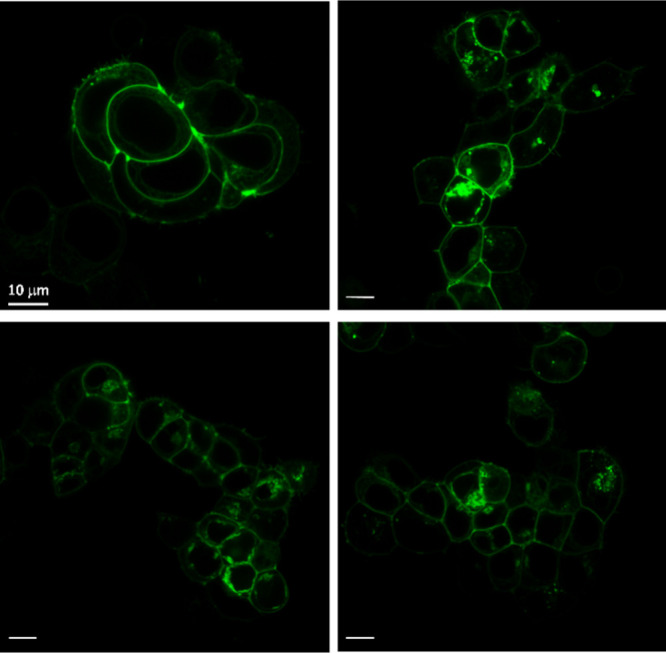
Images with the STED function of living cells expressing HA–M_2_R stained with **33**. The green color represents **33** (300 nM) labeling. Scale bar: 10 μm. Representative images of two independent experiments are shown. For control STED images, see Figure S10.

In conclusion, the diverse functions and structural characteristics of the M_2_ receptor make it a critical target for understanding its role under various physiological and pathological conditions. Extending the possibilities of molecular level investigations, we have developed a new anthranilamide-based fluorescent probe for M_2_R. The conjugate has a nanomolar affinity toward M_2_R (*K*_i_ = 2.4 nM) and demonstrates greater selectivity compared to the previously reported dibenzodiazepinone derivatives. The OG488-labeled probe (**33**) exhibits suitable photophysical properties (λ_abs_^max^: 520, λ_max_^em^: 547, ε: 42504.9 M^–1^ cm^–1^, Φ_f_: 0.20), making it highly suitable for microscopy. In order to demonstrate its versatility, we successfully applied it in confocal microscopy, two-photon microscopy imaging, and STED imaging with living cells. Due to the selected Oregon Green 488 dye, the ideal kinetic properties, and beneficial selectivity profile, the probe has the potential to unlock new avenues for investigating M_2_R trafficking, interactions, and its involvement in diverse signaling pathways at high resolution with a broad range of microscopic methods.

## Supplementary Material



## Notes

General chemistry methods; computational modeling; photophysical measurements; radioligand competition binding studies; in vitro pharmacology; microscopy imaging; synthetic procedures; ^1^H and ^13^C NMR spectra (PDF)

## Abbreviations

M_2_R: M_2_ muscarinic acetylcholine receptor
GPCRs: G protein-coupled receptors
STED: stimulated emission depletion nanoscopy
mAChRs: muscarinic acetylcholine receptors
Ach: acetylcholine
AD: Alzheimer’s disease
OG488: Oregon Green 488
IFD: induced fit docking
PEG: polyethylene glycol
PBS: phosphate buffered saline
BRET: bioluminescence resonance energy transfer
CHO: Chinese hamster ovary
HEK: human embryonic kidney
NMR: nuclear magnetic resonance
HRMS: high-resolution mass spectrometry
