# Supplementary material for: Design, Synthesis, and Evaluation of a New Fluorescent Ligand for the M2 Muscarinic Acetylcholine Receptor
Source: ACS Med Chem Lett. 2025 Mar 20;16(4):552–9. doi: 10.1021/acsmedchemlett.4c00592 (PMC11995211; doi:10.1021/acsmedchemlett.4c00592)
Supplement: Supplementary file 1 [file ml4c00592_si_001.pdf]

Supporting Information

Design, Synthesis, and Evaluation of a New Fluorescent Ligand for the M$_2$ Muscarinic Acetylcholine Receptor

Renáta Szabó[a,b,c], Dénes Szepesi Kovács[a,b,c], Dóra Judit Kiss[a,c], Zeinab Nezafat Yazdi[d], András Dávid Tóth[d,e], Jose Brea[f], María Isabel Loza[f], Domokos Meszéna[g,h], Lucia Wittner[c,h], István Ulbert [c,h,i,j], Balázs Volk[k], László Hunyady[d,l] and György Miklós Keserű[a,b,c]*

AUTHOR ADDRESS

[a] Medicinal Chemistry Research Group, HUN-REN Research Centre for Natural Sciences, H-1117 Budapest, Hungary

[b] Department of Organic Chemistry and Technology, Budapest University of Technology and Economics, H-1111 Budapest, Hungary

[c] National Laboratory for Drug Research and Development, H-1117 Budapest, Hungary

[d] Institute of Molecular Life Sciences, Centre of Excellence of the Hungarian Academy of Sciences, HUN-REN Research Centre for Natural Sciences, H-1117, Budapest, Hungary

[e] Department of Internal Medicine and Haematology, Semmelweis University, H-1088 Budapest, Hungary

[f] Innopharma Drug Screening and Pharmacogenomics Platform. BioFarma research group. Center for Research in Molecular Medicine and Chronic Diseases (CiMUS). Department of Pharmacology, Pharmacy, and Pharmaceutical Technology. University of Santiago de Compostela, Santiago de Compostela, Spain

[g] Department of Neurology, Center for Neurotechnology and Neurorecovery, Massachusetts General Hospital, Harvard Medical School, Boston, MA 02114, USA

[h] Integrative Neuroscience Research Group, Institute of Cognitive Neuroscience and Psychology, HUN-REN Research Centre for Natural Sciences, H-1117 Budapest, Hungary

[i] Department of Neurosurgery and Neurointervention, Semmelweis University, H-1145, Budapest, Hungary

[j] Department of Information Technology and Bionics, Péter Pázmány Catholic University, H-1083 Budapest, Hungary

[k] Egis Pharmaceuticals Plc., P.O. Box 100, H-1475 Budapest, Hungary

[l] Department of Physiology, Faculty of Medicine, Semmelweis University, H-1094 Budapest, Hungary

* keseru.gyorgy@ttk.hu

Table of Contents

General Chemistry Methods 2

Computational Modeling 3

Photophysical Measurements 3

Radioligand Competition Binding Studies 6

In vitro Pharmacology 7

Microscopy Imaging 10

Synthetic Procedures 18

Notes and references 49

General Chemistry Methods

The melting points were measured using a Jasco SRS OptiMelt apparatus (Stanford, CA, USA) and are reported without correction. ^{1}H and ^{13}C NMR spectra were recorded at room temperature in $CDCl_3$ or DMSO-d_6 solutions on a Varian Unity Inova 500 spectrometer (500 and 125 MHz for ^{1}H and ^{13}C NMR spectra, respectively), and on Varian Unity Inova 300 spectrometer (300 and 75 MHz for ^{1}H and ^{13}C spectra, respectively). The deuterium signal of the solvent served as the lock, and TMS was used as the internal standard. Chemical shifts (δ) are given in ppm, and coupling constants (J) are reported in Hz. High-resolution mass spectra (HRMS) were obtained using a Waters Q-TOF Premier mass spectrometer operating in positive ESI ionization mode or in positive APCI ionization mode. The reactions were monitored by analytical thin-layer chromatography (TLC) on silica gel 60 F_{254} plates and HPLC-MS analysis on a Shimadzu LCMS-2020 instrument (Shimadzu Corporation, Kyoto, Japan). The HPLC system utilized a Reprospher 100 C18 column (5 µm, 100 × 3 mm) with a positive-negative double ion source (DUIS±) and a quadrupole mass analyzer covering a range of 50–1000 m/z. Samples were eluted using gradient elution with eluent A (0.1% HCOOH in H_2O) and eluent B (0.1% HCOOH in MeCN) at a flow rate of 1.5 mL/min. The column temperature was maintained at 30 °C, and the injection volume was 1 µL. The detection was carried out at wavelengths of 190 and 254 nm. Two methods were used for sample elution. In the first method, the gradient started at 0% eluent B, increased linearly to 100% by 2 minutes, and was maintained at 100% eluent B from 2 to 3.75 minutes. From 3.75 to 4.5 minutes, the gradient returned to the initial condition (0% B) and was held until 5 minutes. In the second method, the gradient started at 0% eluent B, increased linearly to 100% by 7 minutes, and was maintained at 100% eluent B from 7 to 8 minutes. From 8 to 9 minutes, the gradient returned to the initial condition (0% B) and was held until 10 minutes.

All reagents were sourced from commercial suppliers such as Sigma-Aldrich (St. Louis, MO, USA), Fluorochem (Hadfield, Derbyshire, UK), and Combi-Blocks (San Diego, CA, USA). Oregon Green™ 488 Succinimidyl Ester was obtained from Thermo Fisher Scientific.

Computational Modeling

All calculations were performed using the Schrödinger Small-Molecule Drug Discovery Suite 2022-1, Schrödinger, LLC, New York, NY, 2022.

For the structure-based modeling we have downloaded the M_2 receptor crystal structure from the GPCRdb database[1] (PDB code: 5ZKB)[2] and prepared it with default settings with the Protein Preparation Wizard[3] available in the Schrödinger Program Package. The ligands were prepared with Ligprep using OPLS4[4] force field. During the procedure all ligands have been protonated at the piperidine ring. For the selected core ligand Induced Fit Docking was applied to determine the plausible binding mode. The grid was centered on the co-crystallized ligand and the outer grid box size was determined automatically based on the co-crystallized ligand size. Side chains within 5 Å of the ligand poses were refined with Prime. The obtained poses were evaluated by their docking score and visual inspection. The stability of selected poses was further assessed with binding pose metadynamics available in the Schrödinger package.[5] The selected structure obtained with IFD and containing the selected binding mode of the core ligand was used to dock the intermediate and dye attached ligands. For the new grid the inner box was centered on the ligand and the inner box size was increased to 15Å × 15Å × 15Å and the outer box size was increased to 51Å × 51Å × 51Å. During the docking calculations carried out with Glide single precision protocol enhanced conformational sampling was used and set to 4 times and post-docking minimization were carried out for all poses.[6] During all calculations hydrogen bond formation with Asp3.32 was constrained.

Photophysical Measurements

Absorbance measurements were performed using a Jasco V-750 Spectrophotometer standard cell quartz cuvette with 1 cm light path length, 1 nm bandwidth, 400 nm/min recording speed) operating at 21 °C. The fluorescence measurements were carried out on a Jasco FP8300 spectrofluorometer in a standard cell quartz cuvette (1 cm light path length). Both the excitation and emission slit widths were both set to 2.5 nm, and the scanning speed was 400 nm/min. Pure solvents were used for blank correction. Absorbance spectra and excitation spectra were recorded in the range of 250–500 nm. For the emission spectra, we excited the sample at the excitation maximum, and measured it from excitation maximum plus 10 nm to 800 nm.

Quantum yields were calculated by recording the fluorescence spectra of a series of different concentrations. The gradient of the integrated fluorescence intensities plotted against absorbance at the excitation wavelength was used for the calculation of the quantum yields:

$$\Phi_x = \Phi_{St} \cdot \left(\frac{Grad_x}{Grad_{St}}\right) \cdot \left(\frac{\eta_x^2}{\eta_{St}^2}\right)$$

The compound **33** was excited at 360 nm, OG488-NHS was used as reference ($\Phi_F^{water} =$ 0.92).[7]

Solvent screening measurements were conducted using 5 μM solutions of the fluorescent probe **33** in various solvents, including acetonitrile, 1,4-dioxane, water, toluene, dichloromethane, tetrahydrofuran, ethanol, PBS buffer (pH=5; 7; 7.4, 9), PBS buffer (pH=7.4) with 1% BSA, and ethyl acetate. Photostability measurements were performed with 5 μM solution of in PBS buffer (pH=7). The samples were exposed to a 4 W light-emitting diode emitting at 450 nm, and emission spectra were recorded.

a)

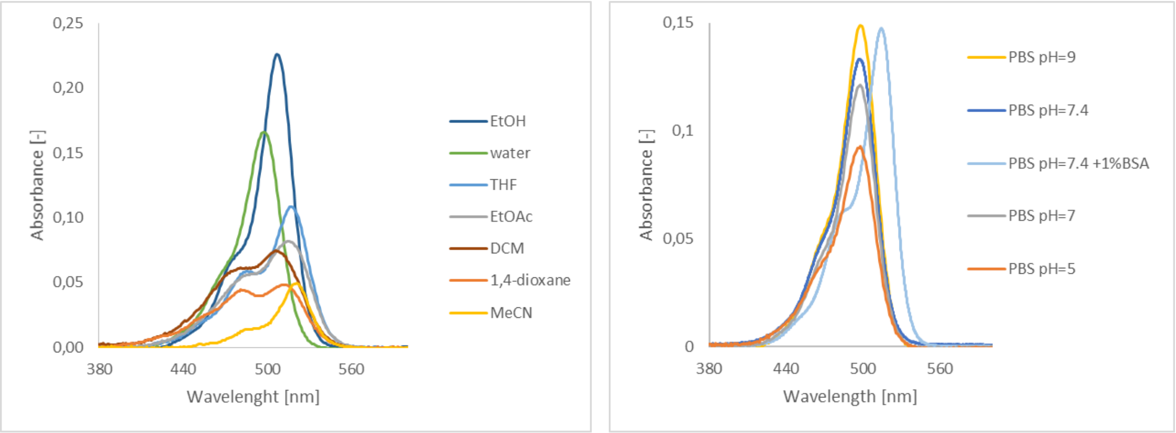

b)

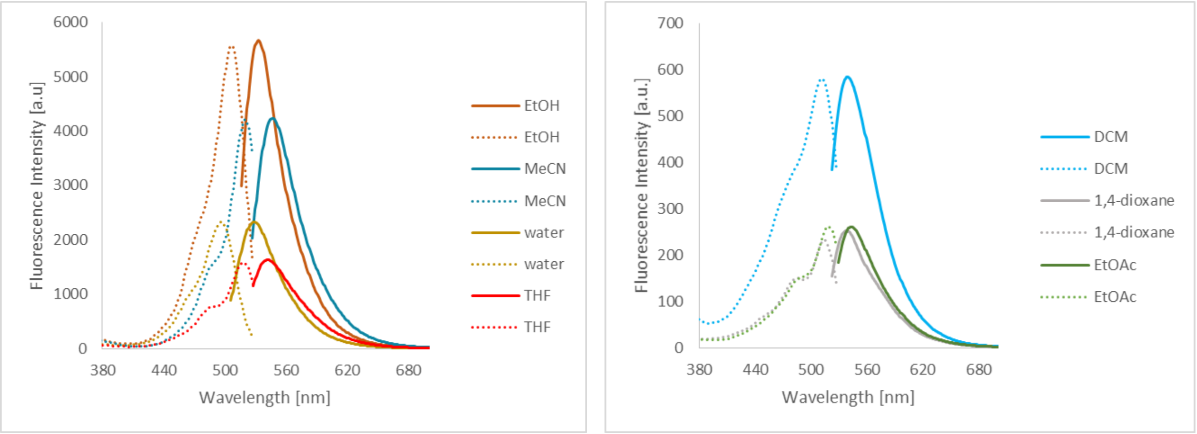

c)

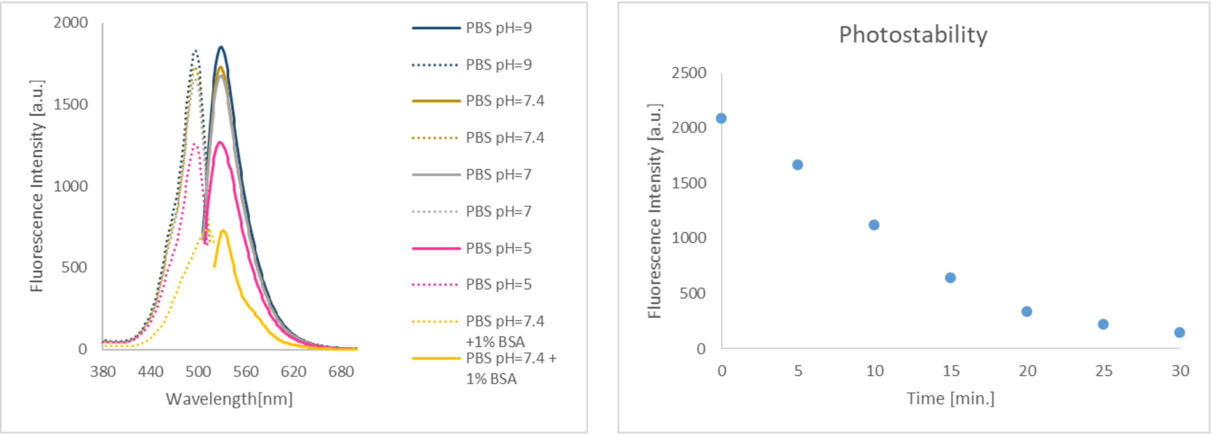

Figure S1. Photochemical properties of **33** (**a**) UV-Vis absorption spectra in different solvents at r.t. (**b**) Fluorescence spectra in different solvents (**c**) (left) Fluorescence spectra in PBS buffers of different pH; (right) Photostability of **33**

Radioligand Competition Binding Studies

The ligands affinities were measured at the INNOPHARMA Drug Screening and Pharmacogenomics Platform.

Competition Binding assays in muscarinic receptors: The assays were run in 96-well polypropylene plates in a total volume of 250 µL by employing membranes overexpressing each of the muscarinic receptors. The conditions for each of the muscarinic subtypes are summarized in **Table S1**. Cell membranes were incubated with [^{3}H]-Scopolamine (*N*-methyl) (78.0 C_i/mmol, 1 mCi/ml, Perkin Elmer NET636250UC) in binding buffer (PBS, pH=7.4) at 27 ^{o}C for 120 min. After this time, 200 µL of the mixture was transferred to a Multiscreen FC 96-well plate (Millipore, Madrid, Spain) that was previously pretreated with 0.5%PEI in binding buffer. The cell suspension was filtered and washed six times with 250 µl of wash buffer (TrisHCl 50 mM, NaCl 154 mM, pH=7.4). Plates were dried and 50 µL Universol was added to each well. Radiactivity was detected n a microplate beta scintillation counter (Microbeta Trilux, PerkinElmer, Madrid, Spain). Non-specific binding was detected in the presence of 5 µM atropine (Sigma).

Data was fitted to 4-parameter logistic equation using Prism v10.2 (Graphpad) and Ki values were derived from the equation:

K_i=IC_{50}/(1+(F/K_D));

where IC_{50} is the value derived from the non-linear fitting, F is the radioligand concentration employed in the assay and K_D is the dissociation constant determined for the radioligand at each of the receptors studied.

Table S1: Conditions employed in radioligand binding assays at each of the receptors studied

Receptor	M_1	M_2	M_3	M_4	M_5
Membrane quantity per well (µg)	15	40	40	20	40
Radioligand concentration (nM)	0.8	0.8	1	0.35	0.8
Radioligand K_D (nM)	0.83	0.81	1.14	0.35	0.77

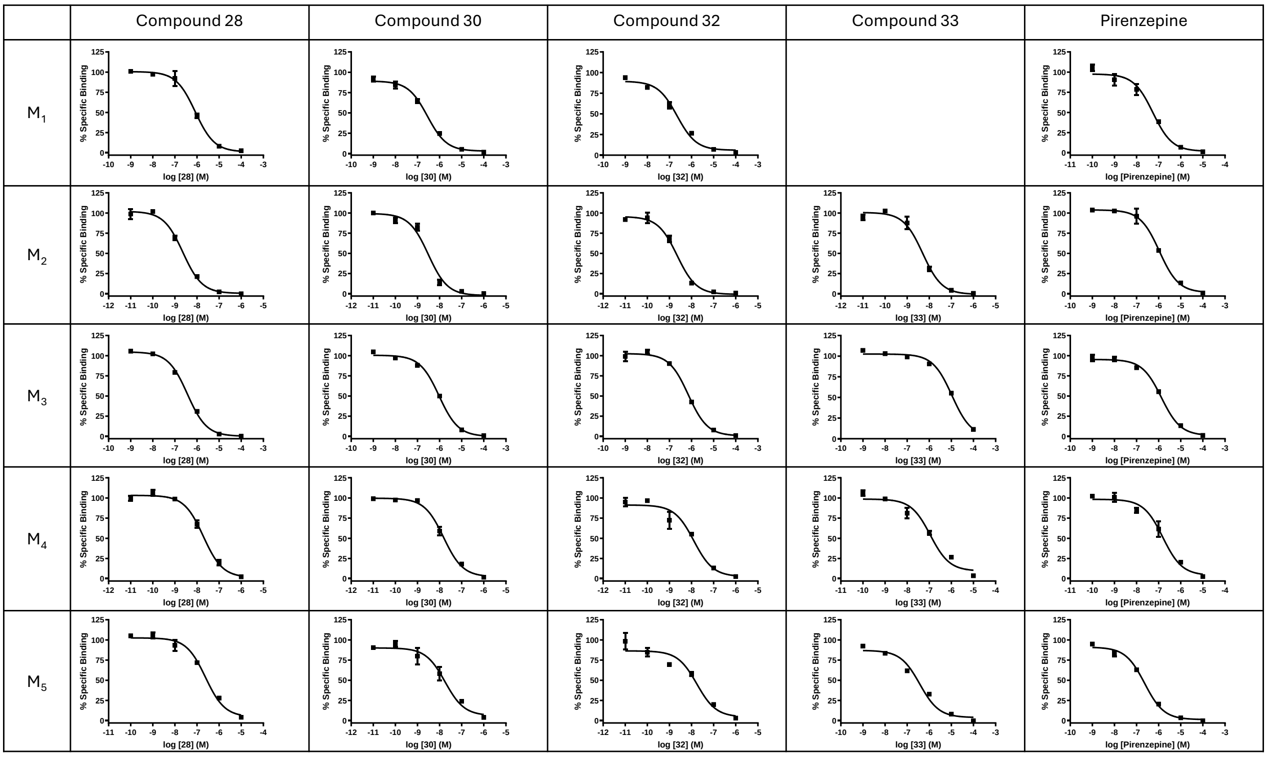

Figure S2. Concentration-percentage specific binding curves of the different compounds studied at the five muscarinic receptor subtypes. The mean ± SD (vertical bars) of each measure determined in duplicate. A representative experiment from a total of three (n=3) is shown.

In vitro Pharmacology

Materials

Prolume Purple and native coelenterazine were purchased from Nanolight (catalog numbers: #369 and #303). Carbachol was obtained from Merck (catalog number: C4382). Atropine was purchased from MedChemExpress (catalog number: HY-B1205A).

Cell line and transfection

HEK 293T cells were obtained from American Type Culture Collection (CRL-3216). Cells were cultured in DMEM supplemented with 10% FBS and 1% penicillin/streptomycin. For BRET measurements, cells were transfected in suspension using the calcium phosphate precipitation method and were seeded onto poly-L-lysine-coated white 96-well plates.[8] For confocal microscopy and stimulated emission depletion (STED) microscopy, cells were seeded onto poly- L-lysine-coated IBIDI µ-Slide 8 well plates. For two-photon microscopy, cells were

seeded onto 6-well plates. The next day, the calcium phosphate precipitation method was applied to transfect the adherent cells.[8]

Plasmid constructs

The PRESTO-Tango and TRUPATH plasmid kits were gifts from Bryan Roth (Addgene kits #1000000068 and #1000000163).[9,10] The coding sequence of the human M_2 muscarinic acetylcholine receptor was PCR amplified from the CHRM2-Tango plasmid (Addgene plasmid # 66249) using a forward primer containing the hemagglutinin (HA) coding sequence and a reverse primer with a stop codon. It was then inserted into pcDNA3.1 vector using EcoRI/XhoI restriction digestion to generate HA–M_2R. To generate GLuc–M_2R (M_2R N-terminally tagged with *Gaussia* luciferase), the sequence encoding the transmembrane domain of the platelet-derived growth factor receptor beta in Gluc–PM construct (Addgene plasmid #164783)[8] was replaced with the PCR-amplified M_2R sequence using BamHI and XhoI restriction sites. To generate M_2R–Cerulean (M_2R C-terminally fused to Cerulean), the sequence encoding M_2R was PCR-amplified and inserted between XhoI and KpnI restriction sites in the Cerulean N1 vector.

Bioluminescence resonance energy transfer (BRET) measurements

BRET and TRUPATH $BRET^2$ measurements were performed 24–28 hours after transfection, as previously described.[10,11] Briefly, HEK 293T cells were washed twice with a modified Krebs–Ringer solution (120 mM NaCl, 10 mM Na-HEPES, 10 mM glucose, 4.7 mM KCl, 1.2 mM $CaCl_2$, 0.7 mM $MgSO_4$, pH 7.4). For TRUPATH measurements, the expression of the GFP2-tagged construct was verified by fluorescence measurement (excitation: 400 nm, emission: 515 nm) using a CLARIOstar Plus multimode plate reader (BMG Labtech).

The TRUPATH G_{oA} assay was used to assess the G protein activation of M_2R in cells coexpressing HA–M_2R, $G\alpha_{oA}$–Rluc8, $G\beta_3$, and $G\gamma_8$–GFP2. For $BRET^2$ measurement, Prolume Purple (1 μM) was used as a substrate. Luminescence intensities of BRET donor and acceptor emission peaks were measured using a CLARIOstar Plus multimode plate reader with 410-80 and 515-30 emission filters. After the addition of the $BRET^2$ substrate, baseline BRET ratios were recorded for 6 minutes. Subsequently, cells were treated with vehicle or increasing concentrations of the test compounds for 20 minutes. This was followed by the addition of 10 μM of the orthosteric agonist carbachol, and the response was monitored for an additional 15 minutes. The BRET response was calculated as the change in BRET ratio before and after carbachol stimulation, normalized to the change in BRET ratio observed in the vehicle-treated

condition and expressed as a percentage. The half-maximal inhibitory concentration (IC_{50}) values for the effects of the compounds on the carbachol response were determined using GraphPad Prism 9 software, applying the log(inhibitor) vs. response (three-parameter) equation with a fixed Hill slope of 1.

BRET-based ligand binding measurements were performed on cells expressing GLuc–M_2R. After the addition of 15 µM native coelenterazine, luminescence intensities were measured using 475-30 nm and 535-30 nm emission filters. Non-specific BRET signals were determined by pretreating cells with 100 µM atropine, a competitive orthosteric M_2R ligand. Increasing concentrations of **33** were used to assess association binding kinetics. Equilibrium ligand binding data were assessed based on the BRET ratios after a 1-hour treatment. For competitive ligand binding measurements, cells were simultaneously treated with increasing concentrations of unlabeled M_2R ligands (atropine or **28**) and 300 nM **33**.After a 1-hour treatment with **33**, dissociation kinetics of **33** were monitored by displacing fluorescent compound with 100 µM atropine treatment.

BRET ratios were calculated by dividing the luminescence intensities of the BRET acceptor and donor emission peaks. The values were normalized to vehicle-treated controls. Additionally, the kinetic data were normalized to baseline BRET ratios. To calculate carbachol-induced TRUPATH GoA activation, the BRET ratios after compound or vehicle pretreatments were subtracted from the carbachol-induced BRET ratios (average of BRET ratios measured after carbachol treatment for 16 minutes). The resulting values were expressed as a percentage of the vehicle-pretreated values. GraphPad Prism 9 software was used for curve fitting. Half-inhibitory concentration (IC_{50}) values were determined using the "log(agonist) vs. response" equation. The "One phase decay" equation was applied to calculate the dissociation rate constant (k_{off}). The "Association kinetics – Two or more conc. of hot." equation was used to determine the association rate constant (k_{on}). For equilibrium ligand binding data, simple linear regression was fitted to the non-specific binding values. The "One site -- Specific binding" and "One site -- Total" equations were fitted to the specific and total binding values, respectively.

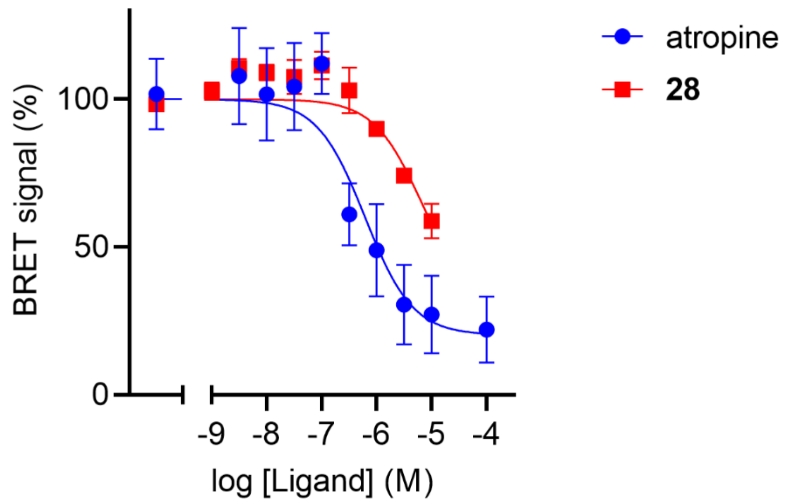

Figure S3. Competitive ligand binding measurements using BRET. HEK 293T cells expressing GLuc–M_2R were treated with 300 nM **33** and the indicated unlabelled M_2R ligand at increasing concentrations. Data are mean ± SEM, with results shown from a representative measurement of three independent experiments. IC_{50} values were 6.1 x 10^{-7} M for atropine and 6.4 x 10^{-6} M for **28**.

Microscopy Imaging

Confocal microscopy

Live HEK 293T cells, transiently transfected and cultured in 8-well IBIDI chamber slides, were investigated using a Zeiss LSM 710 confocal microscope (Zeiss, Jena, Germany). Cerulean fluorescence was excited using a 458 nm laser, with emission detected in the 465–513 nm range. **33** was excited using a 514 nm laser, and its emission was detected in the 519–797 nm range. Prior the imaging, the cell culture medium was removed and the cells were washed twice with modified Krebs–Ringer solution. A baseline image (zero timepoint) was captured before the addition of 300 nM **33**. Subsequent images were captured every minute for 3 minutes in the Cerulean, **33,** and phase-contrast channels. After 3 minutes, the cells were washed with modified Krebs–Ringer solution to remove unbound **33** molecules, followed by repeated image acquisition. The experiment was performed with atropine pretreatment as well. Atropine was added before the compound **33** in 100 µM concentration. After 5 minutes incubation **33** was added in 300 nm and the imaging was performed as described above.

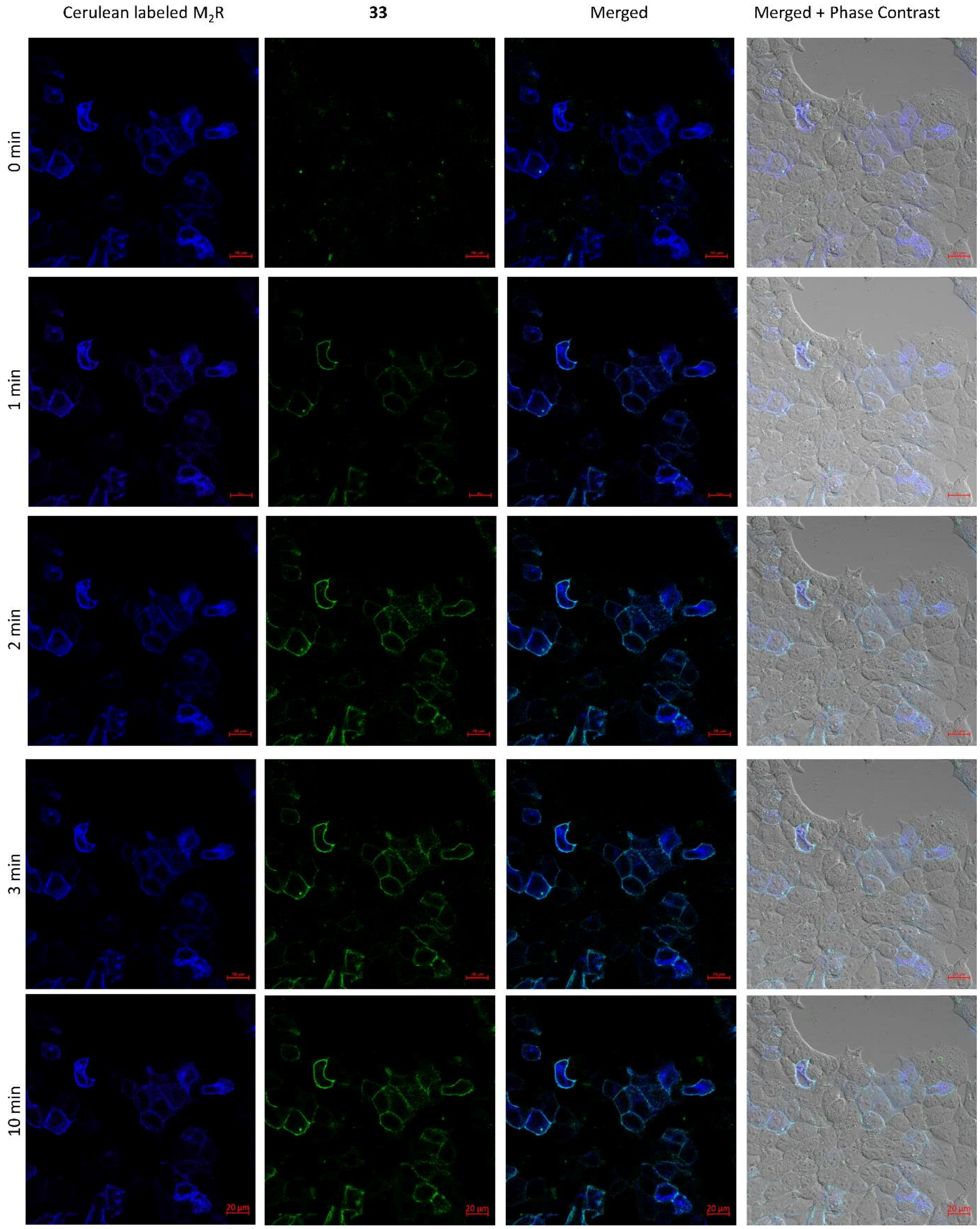

Figure S4. Confocal images of **33**-labelled live, M_2R–Cerulean-transfected HEK 293T cells, captured at 0, 1, 2, 3, and 10 minutes after treatment. Scale bars: 20 µm. Representative images of two independent experiments are shown.

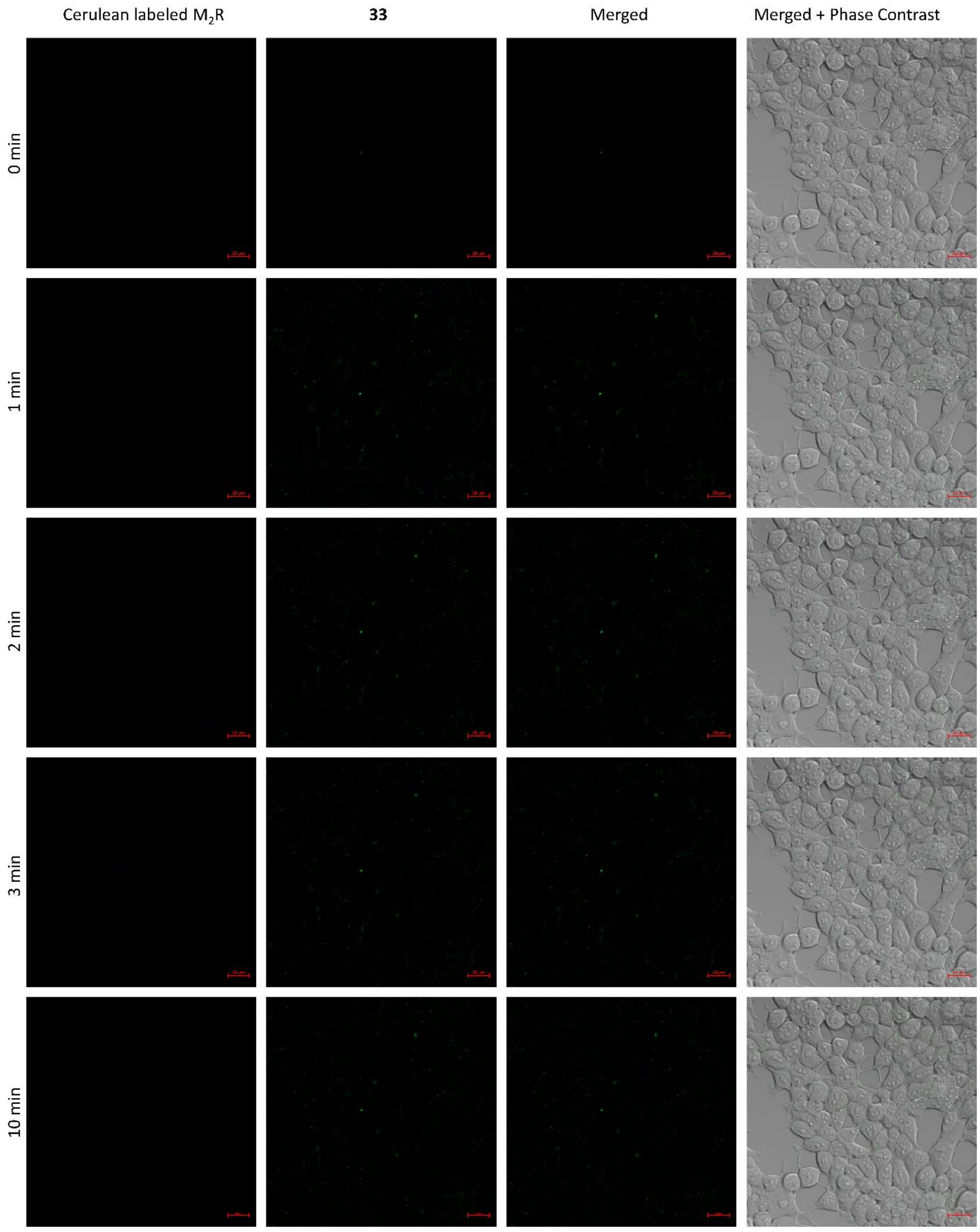

Figure S5. Confocal images of **33**-labelled live, non-transfected HEK 293T cells captured at 0, 1, 2, 3, and 10 minutes. Scale bars: 20 µm. Representative images of two independent experiments are shown.

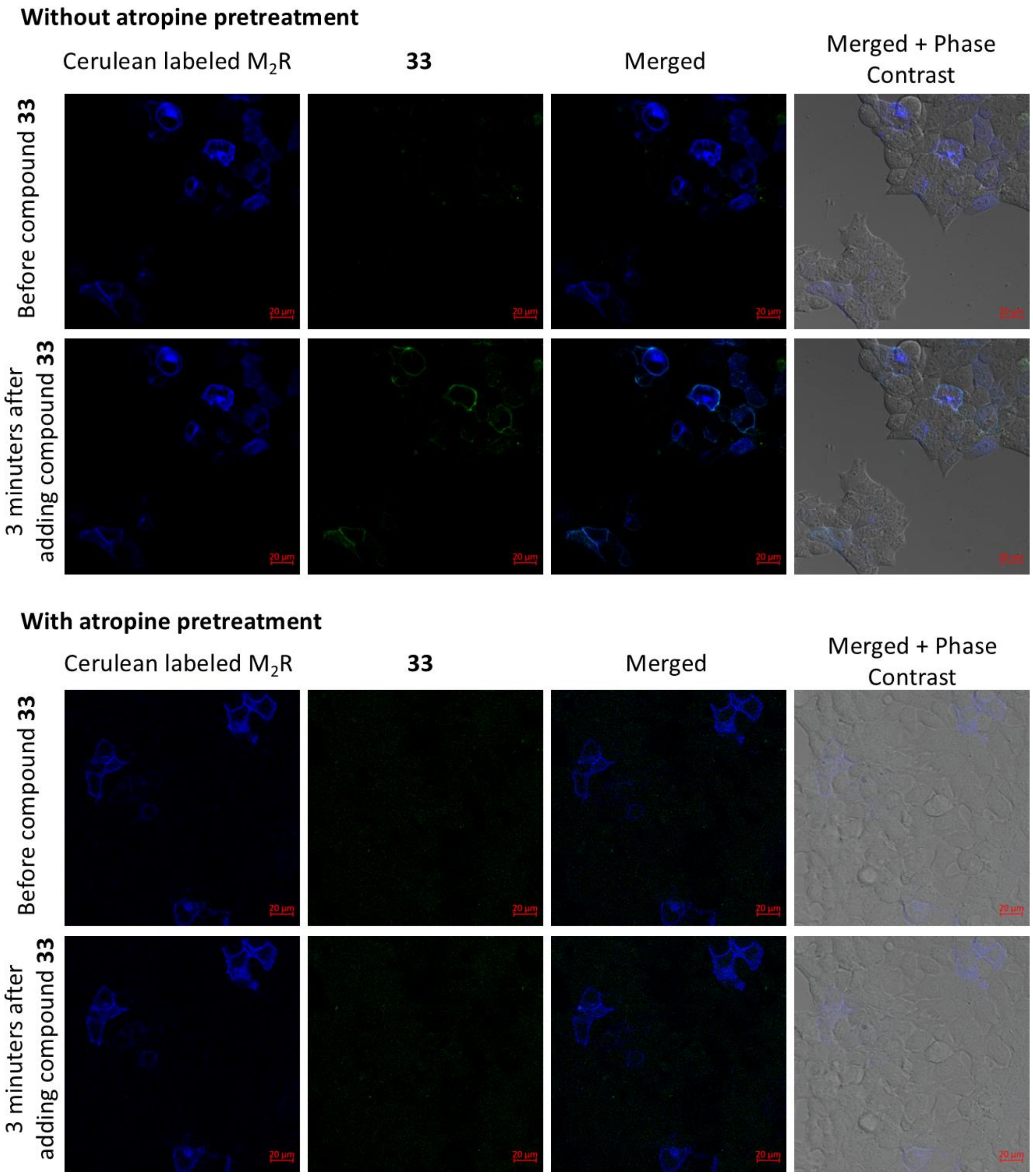

Figure S6 Confocal images of **33**-labelled live M_2R-transfected HEK 293T cells with and without atropine pretreatment (100 µM), captured at 0 and 3 minutes. Scale bars: 20 µm. Representative images from two independent experiments are shown.

Two-photon microscopy

HEK 293T cells, cultured in 6-well plates and transiently transfected with HA–M_2R plasmid, were examined using a two-photon laser scanning system (Femto2D-uncage, Femtonics Ltd., Budapest, Hungary). The imaging laser (Chameleon Ultra II, Coherent Ltd., Santa Clara, CA, USA) was set to 820 nm and to 2-3% intensity. The excitation was delivered to the slices and the fluorescent signal was collected using an XLUMPlanFI 20x lens (Olympus, Tokyo, Japan). Before image acquisition, cells were washed twice, and the medium was replaced to modified Krebs–Ringer solution. After capturing the zero-point image, we added 300 nM **33**. After two minutes we captured images in **33** and infrared transmission channels. After 3 minutes, unbound **33** was removed by washing the cells with modified Krebs–Ringer solution, and the image capture was repeated.

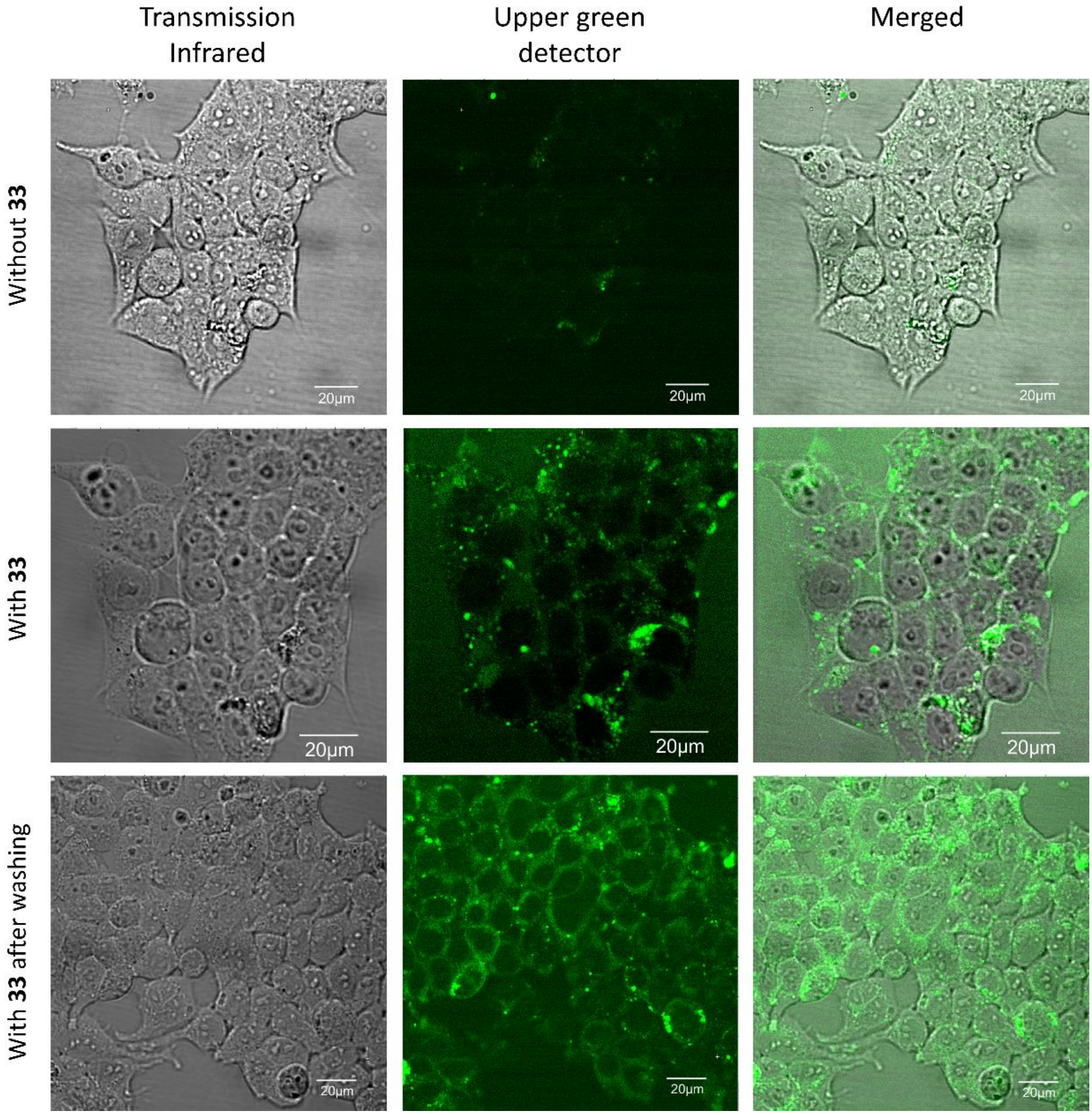

Figure S7. Two-photon images of **33** in live, M_2R transfected HEK 293T live cells. Scale bar: 20 µm. Representative images of two independent experiments are shown.

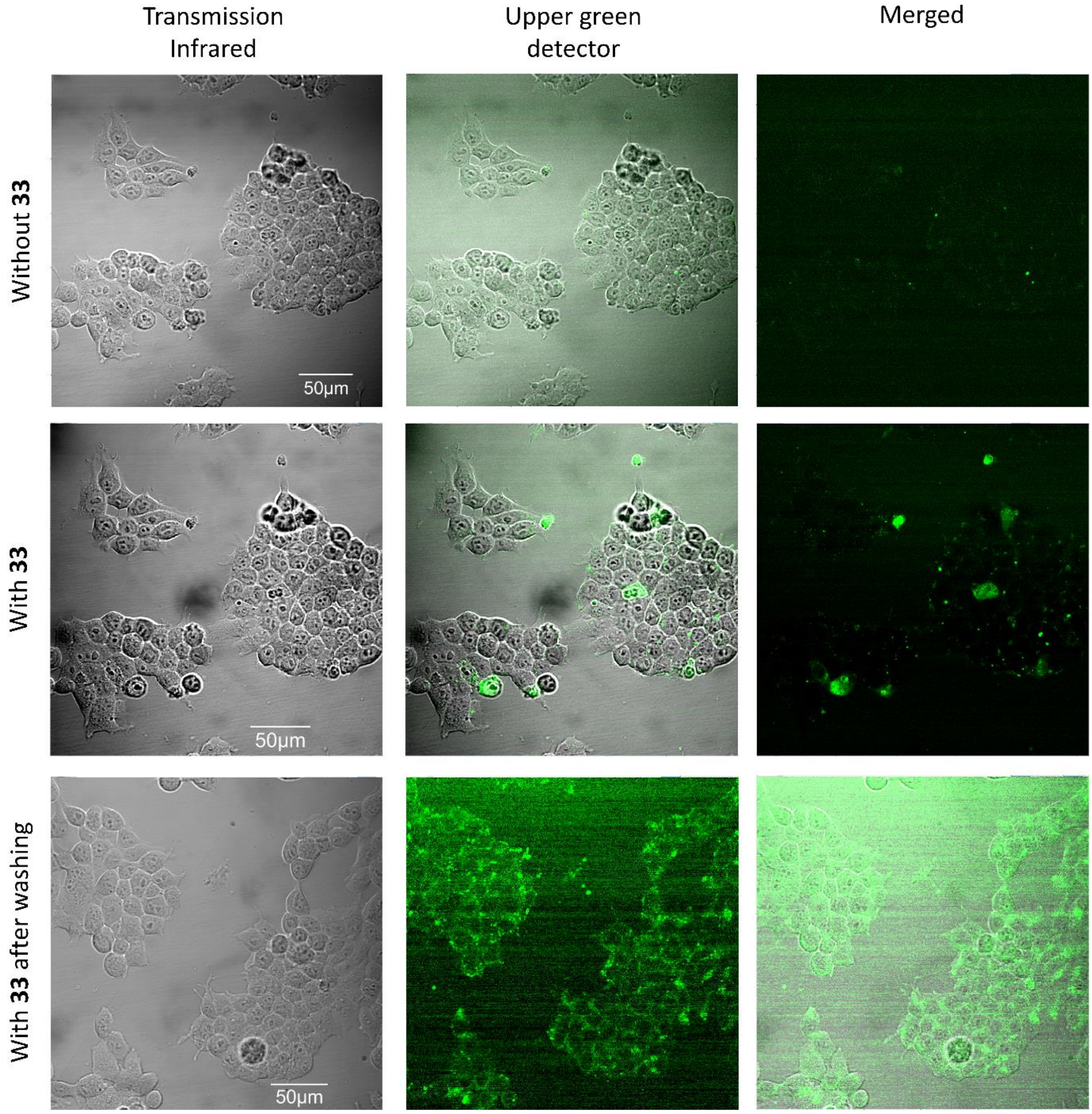

Figure S8. Two-photon images of **33** in live, non-transfected HEK 293T live cells. Scale bar: 50 µm. Representative images of two independent experiments are shown.

Confocal and STED microscopy

Confocal and STED images were acquired using a Leica TCS SP8 STED 3× inverse microscope. HEK 293T cells were transfected with HA-M_2R plasmid and were cultured in 8-well IBIDI chamber slides. The samples were scanned with a Leica HC PL Fluotar 20x/0.55 objective and a PMT detector. A 488 nm laser was used for the excitation of the fluorescent dye OG488 for the confocal imaging, and the spectral detection parameters were 500-550 nm.

Simultaneous transmission light imaging was used to detect the cells. For STED imaging, we used the same excitation laser and a Leica HC PL APO 100×/1.40 oil immersion objective and a Leica HyD detector, which was completed with a 660 nm STED (1.5 W, continuous wave) laser for depletion (at 1.17–2.48 × 10^9 W mm^{-2} nominal intensity with an output laser power of 1.2 W/32 nm × 32 nm pixel size). Deconvolution was performed on the STED images using the Huygens Professional software (SVI), which was based on the theoretical point spread function (PSF).

Prior to image acquisition, the cell culture medium was replaced to modified Krebs–Ringer solution, including two washing steps. After taking a baseline image, 300 nM **SzR-M2-61** was added, and subsequent images were made after two minutes. After 3 minutes, unbound **33** was removed by washing with modified Krebs–Ringer solution, and additional images were captured.

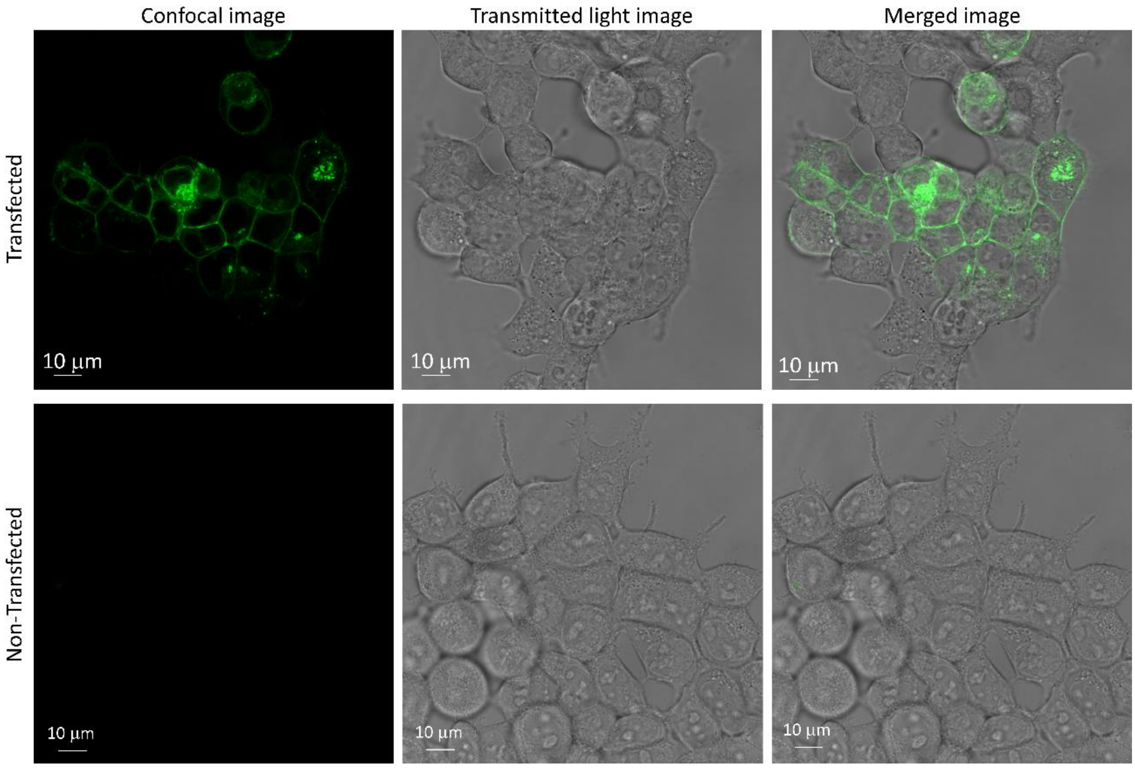

Figure S9. Confocal images of **33** labelling of live, HA–M_2R transfected and non-transfected HEK 293T cells acquired using a Leica TCS SP8 STED 3× inverse microscope. Scale bar: 10 μm. Representative images of two independent experiments are shown.

Images were also taken from control HEK293 cells with STED microscopy. As these cells do not express M_2R on their cell membrane, derivative **33** was not bound, and consequently fluorescence did not appear. All these images are totally black, and therefore were not shown.

By definition, the STED function of the microscope cannot be used together with transmitted light imaging, and therefore we confirmed with confocal microscopy completed with transmitted light imaging that the living cells were present.

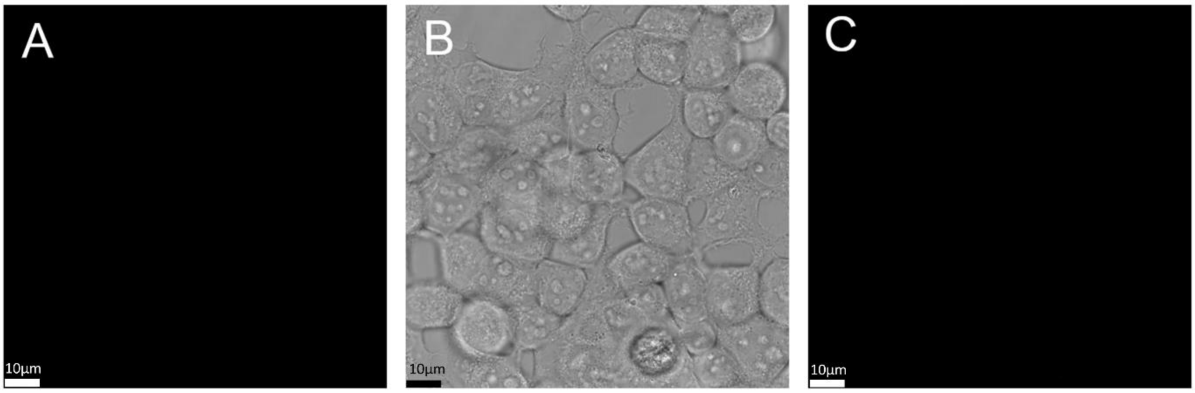

Figure S10. Images with STED function of non-transfected HEK 293T live cells stained with **33** (300 nM). Scale bar: 10 µm. Representative images of two independent experiments are shown.

A) STED microscopy image of control (M_2R-negative) HEK293 cells. The cells do not bind the compound **33**, and therefore fluorescence is lacking.
B) Transmitted light image of the same region of interest. Living cells are present.
C) Confocal image of the same region of interest taken together with the transmitted light image. No fluorescence appears.

Synthetic Procedures

1-((4-Bromophenyl)sulfonyl)-3-chlorobenzene (**14**)

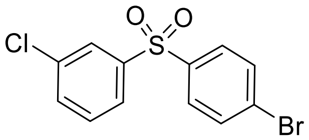

In a round bottom flask 3-chlorobenzenesulfonyl chloride (**13**, 20.00 mmol, 1.0 equiv., 4.22 g, 2.82 mL), bromobenzene (40.00 mmol, 2.0 equiv., 6.28 g, 4.21 mL) and $AlCl_3$ (20.00 mmol, 1.0 equiv., 2.67 g) were combined, and the mixture was stirred at 80 °C for 30 min. After completion of the reaction (monitored by HPLC-MS) the unreacted bromobenzene was removed by evaporation. Then, water (200 mL) was added and the mixture was cooled in ice water for 1 h. The desired product was collected on a filter, washed with water (2 × 50 mL) and air dried. Yield: 5.52 g (81%), white powder, m. p. 95-98 °C (water). ^1H NMR ($CDCl_3$, 300 MHz) δ 7.90 (s, 1H), 7.80 (d, *J* = 8.5 Hz, 3H), 7.66 (d, *J* = 8.6 Hz, 2H), 7.55 (d, *J* = 8.1 Hz, 1H), 7.45 (t, *J* = 7.9 Hz, 1H). ^{13}C NMR ($CDCl_3$, 75 MHz) δ 143.0, 140.1, 135.7, 133.7, 132.9, 130.9, 129.4, 129.1, 127.8, 125.9. HRMS ($APCI^+$) m/z$[M+H]^+$, calcd. for $C_{12}H_9ClO_2S$: 330.9190 found: 330.9190. Purity by HPLC: 100%

***tert*-Butyl-4-(4-((3-chlorophenyl)sulfonyl)benzyl)piperidine-1-carboxylate** (**17**)

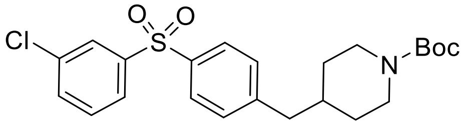

Compound **17** was synthetized using the method described by *Vice S.* et al with some modifications.[12] To tert-butyl 4-methylenepiperidine-1-carboxylate (**15**, 3.35 mmol, 1 equiv., 660.9 mg, 661 µL) was added 9-BBN (6.70 mL of a 0.5 M solution in THF, 3.35 mmol, 1 equiv.) and was heated for 1 h at 65 °C under argon atmosphere. Then to the resulting solution were added the **14** (2.68 mmol, 0.8 equiv., 888.7 mg), Pd(dppf)Cl_2 · CH_2Cl_2 (0.10 mmol, 0.03 equiv., 73.5 mg), DMF (7 mL), water (660 µL), and K_2CO_3 (4.02 mmol, 1.2 equiv., 555.6 mg) and the mixture was allowed to stir at 60 °C for 3 h. After cooling the mixture to room temperature and pouring it into water (5 mL), the pH was adjusted to 11 using 10% aqueous NaOH. The mixture was then extracted with DCM (3 × 15 mL), and the combined organic layers were dried over $MgSO_4$, filtered and evaporated *in vacuo*. The crude residue was purified by flash chromatography on silica gel using hexane/ethyl acetate 4:1. Yield: 1.17 g (83%), yellow solid, m. p. 125 °C (hexane/ethyl acetate). ^1H NMR ($CDCl_3$, 300 MHz) δ 7.91 (t, *J* = 1.8 Hz, 1H), 7.86 – 7.79 (m, 3H), 7.54 – 7.49 (m, 1H), 7.43 (t, *J* = 7.8 Hz, 1H), 7.26 (d, *J* = 8.8

Hz, 2H), 4.12 – 3.96 (m, 1H), 2.63 (d, *J* = 12.6 Hz, 1H), 2.58 (d, *J* = 7.1 Hz, 2H), 1.91 – 1.77 (m, 1H), 1.70 – 1.50 (m, 4H), 1.44 (s, 9H), 1.27 – 1.04 (m, 2H). ^{13}C NMR ($CDCl_3$, 75 MHz) δ 154.9, 147.0, 143.7, 138.7, 135.6, 133.4, 130.7, 130.2, 128.0, 127.8, 125.8, 79.5, 43.1, 38.0, 32.2, 32.0, 28.6, 26.3, 22.1. HRMS (APCI$^+$,-Boc) m/z[M+H]$^+$, calcd. for $C_{18}H_{21}ClO_2S$: 350,0976 found: 350,0987. Purity by HPLC: 100%

4-(4-((3-Chlorophenyl)sulfonyl)benzyl)piperidine TFA salt (18)

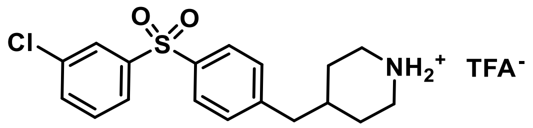

To a solution of compound **17** (2.5 mmol, 1 equiv., 1.12 g) in dichloromethane (10 mL) water (120 µL) was added and the mixture was cooled at 0 °C. Trifluoroacetic acid (37.5 mmol, 15 equiv., 2.87 mL, 4.28 g) was added dropwise over 10 min. The cooling bath was removed and the mixture was allowed to stir for 2 h at room temperature. Then, the solution was evaporated and diethyl ether (50 mL) was added. The mixture was cooled in ice bath for 1 h, the desired product (**18**) was collected on a filter and washed with diethyl ether (2 × 50 mL) and air dried. Yield: 1.08 g (93%), yellow powder, m. p. 199 °C (diethyl ether). ^{1}H NMR (DMSO-d_6, 300 MHz) δ 8.68 (s, 1H), 8.38 (s, 1H), 8.01 (s, 1H), 7.94 (t, *J* = 6.4 Hz, 3H), 7.77 (d, *J* = 7.9 Hz, 1H), 7.65 (t, *J* = 7.9 Hz, 1H), 7.46 (d, *J* = 8.0 Hz, 2H), 3.22 (d, *J* = 12.0 Hz, 2H), 2.78 (d, *J* = 10.6 Hz, 2H), 2.62 (d, *J* = 6.8 Hz, 2H), 1.82 (s, 1H), 1.65 (d, *J* = 13.4 Hz, 2H), 1.30 (dd, *J* = 23.4, 11.2 Hz, 2H). ^{13}C NMR (DMSO-d_6, 75 MHz) δ 146.5, 143.3, 138.1, 134.3, 133.7, 131.8, 130.5, 127.7, 126.8, 126.1, 43.0, 41.1, 34.5, 28.0. HRMS (ESI$^+$) m/z[M+H]$^+$, calcd. for $C_{18}H_{21}ClNO_2S$: 350.0982, found: 350.0970. Purity by HPLC: > 99.9%

tert-Butyl 4-(4-((3-chlorophenyl)sulfonyl)benzyl)-[1,4'-bipiperidine]-1'-carboxylate (**19**)

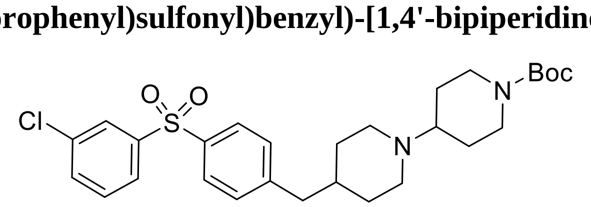

A round-bottom flask was charged with the aforementioned TFA salt (**18**, 2.20 mmol, 1 equiv., 1.02 g), 1-Boc-4-bromopiperidine (2.42 mmol, 1.1 equiv., 639.3 mg), K_2CO_3 (4.40 mmol, 2 equiv., 608.1 mg) and KI (1.10 mmol, 0.5 equiv., 182.6 mg) and MeCN (15 mL) was added. The resulting suspension was stirred for 24 h at 80 °C. The reaction mixture was diluted with

DCM (10 mL) and 1N NaOH (20 mL). The two layers were separated and the aqueous layer was extracted with DCM (3 × 15 mL). The organic phase was washed with water (10 mL), brine (10 mL), dried over MgSO4, filtered and evaporated *in vacuo*. The crude solid was purified by silica gel chromatography using hexane/ethyl acetate 1:1. Yield: 910 mg (78%), beige solid, m. p. 135 °C (hexane/ethyl acetate). ^{1}H NMR ($CDCl_3$, 300 MHz) δ 8.45 (s, 1H), 7.87 (s, 1H), 7.80 (t, *J* = 7.7 Hz, 3H), 7.50 (d, *J* = 8.2 Hz, 1H), 7.42 (t, *J* = 7.8 Hz, 1H), 7.26 (d, *J* = 8.5 Hz, 1H), 4.17 (d, *J* = 10.4 Hz, 2H), 3.22 (d, *J* = 11.1 Hz, 2H), 3.05 – 2.89 (m, 1H), 2.74 – 2.56 (m, 4H), 2.52 – 2.37 (m, 2H), 1.91 (d, *J* = 11.8 Hz, 2H), 1.74 – 1.58 (m, 5H), 1.48 (dt, *J* = 8.5, 6.3 Hz, 2H), 1.40 (s, 8H). ^{13}C NMR ($CDCl_3$, 75 MHz) δ 167.9, 154.4, 146.4, 143.5, 138.9, 135.5, 133.4, 130.7, 130.2, 128.0, 127.7, 125.8, 80.1, 62.7, 48.6, 42.9, 42.2, 36.8, 29.8, 28.4, 26.5. HRMS (ESI$^+$) m/z[M+H]$^+$, calcd. for $C_{28}H_{38}ClN_2O_4S$: 533.2241, found: 533.2242. Purity by HPLC: 95%

2-((2,2-Dimethyl-4-oxo-3,8,11-trioxa-5-azatridecan-13-yl)amino)benzoic acid (23)

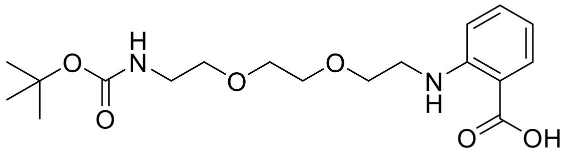

Compound **23** was prepared following the methods described by *Suzuki T.* et al.[13] and *Zeng L.* et al. with some modification.[1]

To a solution of diamine **22** (28.0 mmol, 7 equiv., 4.11 mL, 4.15 g) in DCM (60 mL) was added Boc_2O (4.0 mmol, 1 equiv., 873.0 mg) at 0 °C and the solution was allowed to stir for 8 h at 0 °C and 16 h at room temperature. The organic phase was washed five times with water (25 mL), dried over $MgSO_4$ and concentrated under vacuum.

A round-bottom flask was charged with a 2-iodobenzoic acid (1.0 mmol, 1 equiv., 248.0 mg), the freshly prepared Boc-protected amine (1.5 mmol, 0.5 equiv., 372.5 mg), CuI (0.1 mmol, 0.1 equiv., 19.0 mg), racemic 1,1’-binaphthyl-2,2’-diol (0.2 mmol, 0.2 equiv., 57.3 mg), K_3PO_4 (3.0 mmol, 3.0 equiv., 636.8 mg) and DMF (1 mL) under argon atmosphere. The mixture was allowed to stir at room temperature for 3 h. To the reaction mixture 2N HCl (10 mL) was added and extracted with ethyl acetate (3 × 20 mL). The combined organic layer was washed with brine, dried over $MgSO_4$ and evaporated *in vacuo*. The crude residue was purified by silica gel chromatography using hexane/ethyl acetate 1:1. Yield: 381.3 mg (69%), yellow oil. ^{1}H NMR ($CDCl_3$, 300 MHz) δ 8.65 (s, 1H), 7.95 (dd, *J* = 8.0, 1.5 Hz, 1H), 7.90 (s, 1H), 7.37 – 7.29 (m, 1H), 6.58 (dd, *J* = 15.1, 7.7 Hz, 2H), 3.79 – 3.69 (m, 4H), 3.69 – 3.63 (m, 2H), 3.60 – 3.52 (m,

2H), 3.36 (dt, *J* = 14.8, 4.9 Hz, 4H), 1.49 (s, 9H). ^{13}C NMR ($CDCl_3$, 75 MHz) δ 172.7, 158.4, 151.9, 134.6, 132.3, 114.5, 111.2, 110.5, 80.8, 70.8, 70.2, 68.9, 58.1, 42.3, 42.2, 28.5. HRMS (ESI^+) m/z$[M+H]^+$, calcd. for $C_{18}H_{29}N_2O_6$: 369.2026, found: 369.2022. Purity by HPLC: 99%

2-((2-(2-(2-Aminoethoxy)ethoxy)ethyl)amino)benzoic acid TFA salt (**24**)

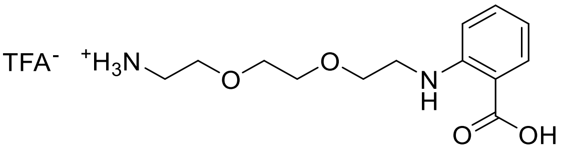

A solution of compound **23** (0.41 mmol, 1 equiv., 150.0 mg) in dichloromethane (3 mL) was cooled at 0 °C and trifluoroacetic acid (4.10 mmol, 10 equiv., 311.0 µL, 464.1 mg) was added dropwise over 10 min. The cooling bath was removed and the mixture was allowed to stir for 2 h at room temperature. Then, the solution was evaporated to give the TFA salt (145.0 mg) as a yellow oil in 93% yield. It was used in the next step without further purification. ^{1}H NMR (300 MHz, $CDCl_3$) δ 10.15 (s, 3H), 7.98 (d, *J* = 7.1 Hz, 1H), 7.72 (s, 2H), 7.46 (t, *J* = 7.2 Hz, 1H), 7.01 (d, *J* = 8.3 Hz, 1H), 6.92 (t, *J* = 7.6 Hz, 1H), 3.77 – 3.68 (m, 4H), 3.64 (s, 4H), 3.43 – 3.37 (m, 2H), 3.28 – 3.19 (m, 2H) ^{13}C NMR (75 MHz, $CDCl_3$) δ 171.3, 145.9, 135.4, 132.8, 121.1, 116.6, 114.8, 70.3, 70.0, 67.6, 66.3, 46.3, 40.1. HRMS (ESI^+) m/z$[M+H]^+$, calcd. for $C_{13}H_{20}N_2O_4$: 269.1501, found: 269.1490. Purity by HPLC: 100%

2-((2,2-Dimethyl-4,14-dioxo-3,8,11,18,21-pentaoxa-5,15-diazatricosan-23-yl)amino)-benzoic acid (**25**)

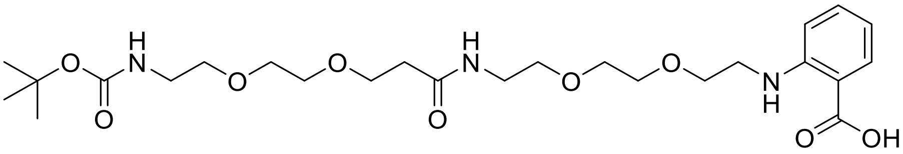

To a stirred solution of *N*-Boc-3-[2-(2-aminoethoxy)ethoxy]propionic acid (**20**, 0.17 mmol, 1 equiv., 47.1 mg) in anhydrous THF (1 mL) EDC · HCl (0.17 mmol, 1 equiv., 35.1 mg) and *N*-hydroxy succinimide (0.17 mmol, 1 equiv., 19.6 mg) were added and the solution was allowed to stir for 2 h. After completion of the reaction (monitored by HPLC-MS) the reaction mixture was filtered.

In another vial, the TFA salt **24** (0.17 mmol, 1 equiv., 65.5 mg) and DIPEA (3.51 mmol, 3 equiv., 71.0 µL, 51.6 mg) were dissolved in anhydrous THF and the solution of above-described NHS-ester was added dropwise. After that, the resulting mixture was stirred for 30 min. at room temperature. The mixture was purified by reversed-phase flash column chromatography using

eluents A (0.1% HCOOH in MeCN) and B (0.1% HCOOH in H_2O). Yield: 54.0 mg (60%), yellow oil. ^1H NMR ($CDCl_3$, 300 MHz) δ 7.93 (d, J = 7.8 Hz, 1H), 7.38 – 7.30 (m, 1H), 6.65 (d, J = 8.5 Hz, 1H), 6.61 – 6.51 (m, 1H), 5.28 (s, 1H), 3.88 – 3.70 (m, 4H), 3.69 – 3.42 (m, 14H), 3.42 – 3.19 (m, 4H), 2.50 (dd, J = 14.4, 9.1 Hz, 2H), 1.41 (d, J = 5.7 Hz, 9H). ^{13}C NMR ($CDCl_3$, 75 MHz) δ 171.8, 151., 134.9, 132.5, 114.7, 111.1, 111.4, 70.41, 70.38, 70.3, 70.12, 70.08, 69.8, 69.1, 68.0, 42.4, 40.4, 40.0, 37.2, 28.4. HRMS (ESI^+) m/z$[M+H]^+$, calcd. for $C_{25}H_{41}N_3O_9$: 528.2921, found: 528.2925. Purity by HPLC: 100%

4-(4-((3-Chlorophenyl)sulfonyl)benzyl)-1,4'-bipiperidine TFA salt (**26**)

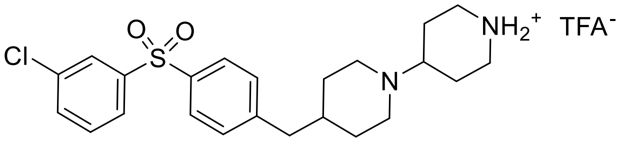

A solution of compound **19** (0.09 mmol, 1 equiv., 50.1 mg) in dichloromethane (1 mL) and water (10 µL) was cooled at 0 °C and dropped trifluoroacetic acid (0.90 mmol, 10 equiv., 72.0 µL, 107.2 mg) over 10 min. The cooling bath was removed and the mixture was allowed to stir for 2 h at room temperature. Then, the solution was evaporated to give the TFA salt (46.8 mg) as a yellow oil in 95% yield. It was used in the next step without further purification. Purity by HPLC: 100%

General method of *N*-acylation:

To a stirred solution of compound Boc-2-Abz-OH (0.02 mmol, 1 equiv., 4.7 mg) or **23** (0.02 mmol, 1 equiv., 7.4 mg) or **25** (0.02 mmol, 1 equiv., 10.5 mg), HOBt · H_2O (0.03 mmol, 1.5 equiv., 4.6 mg), EDC · HCl (0.03 mmol, 1.5 equiv., 5.8 mg), DIPEA (0.06 mmol, 3 equiv., 10 µL, 7.8 mg) in DMF (0.5 mL), **26** (0.02 mmol, 1 equiv., 10.9 mg) was added and the mixture was allowed to stir at 25 °C for 16 h. The mixture was purified by reversed-phase flash column chromatography using eluents A (0.1% HCOOH in MeCN) and B (0.1% HCOOH in H_2O).

***tert*-Butyl-(2-(4-(4-((3-chlorophenyl)sulfonyl)benzyl)-[1,4'-bipiperidine]-1'-carbonyl)-phenyl)carbamate** (**27**)

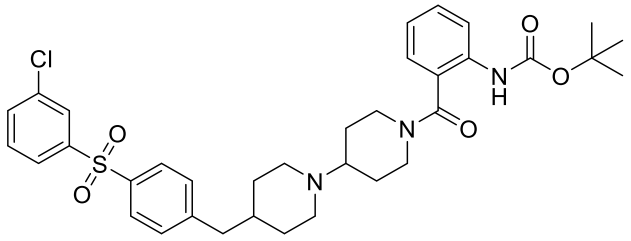

Yield: 13.0 mg (55%), colorless oil. ^{1}H NMR (DMDO-d_6, 300 MHz) δ 8.79 – 8.70 (m, 1H), 8.00 (s, 1H), 7.93 (d, *J* = 7.3 Hz, 3H), 7.74 (d, *J* = 7.8 Hz, 1H), 7.63 (t, *J* = 7.8 Hz, 1H), 7.52 (d, *J* = 8.1 Hz, 1H), 7.42 (d, *J* = 6.8 Hz, 2H), 7.34 (t, *J* = 7.6 Hz, 1H), 7.23 (d, *J* = 7.0 Hz, 1H), 7.10 (t, *J* = 7.2 Hz, 1H), 5.74 (s, 1H), 4.50 (br, 1H), 3.70 – 3.42 (m, 2H), 3.13 – 2.67 (m, 4H), 2.57 (d, *J* = 9.0 Hz, 2H), 2.46 – 2.29 (m, 2H), 1.94 – 1.45 (m, 7H), 1.40 (s, 9H), 1.33 – 1.11 (m, *J* = 12.6 Hz, 2H). ^{13}C NMR (DMSO-d_6, 75 MHz) δ 167.4, 152.8, 147.1, 143.3, 138.0, 135.9, 134.3, 133.6, 131.8, 130.4, 129.6, 127.6, 127.6, 126.7, 126.0, 123.2, 122.9, 79.2, 61.6, 54.9, 48.5, 44.5, 41.4, 36.1, 30.4, 28.0, 21.1. HRMS (APCI^{+}) m/z[M+H]$^{+}$, calcd. for $C_{35}H_{43}ClN_3O_5S$: 652.2607, found: 652.2582. Purity by HPLC: 100%

***N*-(2-(2-(2-((2-(4-(4-((3-Chlorophenyl)sulfonyl)benzyl)-[1,4'-bipiperidine]-1'-carbonyl)-phenyl)amino)ethoxy)ethoxy)ethyl)pivalamide** (**29**)

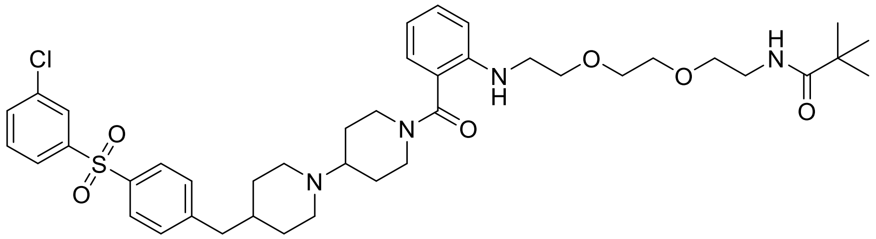

Yield: 7.8 mg (50%), yellow oil. ^{1}H NMR (DMSO-d_6, 300 MHz) δ 8.53 (br, 1H), 7.99 (t, *J* = 1.8 Hz, 1H), 7.90 (t, *J* = 6.1 Hz, 3H), 7.76 (t, *J* = 6.4 Hz, 1H), 7.68 – 7.60 (m, 1H), 7.41 (t, *J* = 6.7 Hz, 2H), 7.23 – 7.14 (m, 1H), 6.99 (t, *J* = 6.2 Hz, 1H), 6.76 – 6.66 (m, 2H), 6.64 – 6.55 (m, 1H), 5.13 (d, *J* = 10.1 Hz, 1H), 4.03 (br, 2H), 3.59 – 3.46 (m, 11H), 3.20 (d, *J* = 5.7 Hz, 2H), 3.05 (d, *J* = 5.9 Hz, 2H), 2.91 – 2.71 (m, 4H), 2.56 (d, *J* = 6.2 Hz, 2H), 2.09 – 1.96 (m, 2H), 1.67-1.64 (m, 2H), 1.47 (d, *J* = 10.6 Hz, 2H), 1.35 (s, 9H), 1.16 (t, *J* = 21.3 Hz, 4H). ^{13}C NMR (DMSO-d_6, 75 MHz) δ 168.3, 147.6, 145.5, 143.3, 137.8, 134.3, 133.6, 131.8, 130.3, 130.2, 127.6, 127.4, 126.7, 126.1, 120.5, 115.5, 111.0, 69.6, 69.5, 69.2, 68.8, 61.2, 48.8, 42.4, 42.0, 37.4, 32.0, 30.7, 28.2. HRMS (ESI^{+}) m/z[M+H]$^{+}$, calcd. for $C_{41}H_{56}ClN_4O_7S$: 783.3558, found: 783.3595. Purity by HPLC: 100%

***tert*-Butyl-(1-((2-(4-(4-((3-chlorophenyl)sulfonyl)benzyl)-[1,4'-bipiperidine]-1'-carbonyl)-phenyl)amino)-10-oxo-3,6,13,16-tetraoxa-9-azaoctadecan-18-yl)carbamate** (**31**)

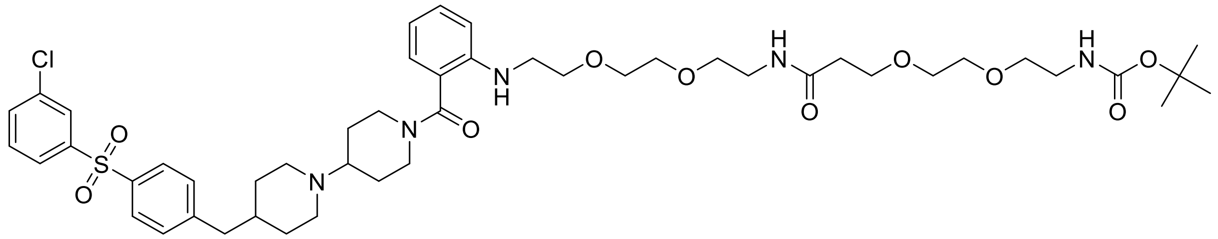

Yield: 9.0 mg (48%), colorless oil. ^{1}H NMR (CD_3CN, 300 MHz) δ 8.32 (s, 1H), 7.94 (t, *J* = 1.8 Hz, 1H), 7.90 – 7.81 (m, 3H), 7.65 – 7.59 (m, 1H), 7.54 (t, *J* = 7.9 Hz, 1H), 7.37 (d, *J* = 8.3 Hz, 2H), 7.27 – 7.18 (m, 1H), 7.07 (dd, *J* = 7.5, 1.3 Hz, 1H), 7.03 (br, 1H), 6.71 (d, J = 8.3 Hz, 1H), 6.64 (t, *J* = 7.3 Hz, 1H), 6.10 (br, 1H), 4.11 (br, 1H), 3.62 (t, *J* = 6.0 Hz, 4H), 3.58 – 3.51 (m, 5H), 3.51 – 3.38 (m, 9H), 3.33 – 3.11 (m, 10H), 2.86 (t, *J* = 12.5 Hz, 2H), 2.65 (dd, *J* = 25.5, 8.2 Hz, 4H), 2.32 (t, *J* = 6.1 Hz, 2H), 2.10 – 1.99 (m, 2H), 1.79 – 1.54 (m, 6H), 1.38 (s, 9H). 13C NMR (CD_3CN, 75 MHz) δ 171.9, 170.3, 166.1, 148.0, 147.2, 144.6, 139.6, 136.0, 134.5, 132.4, 131.8, 131.3, 128.8, 128.7, 128.1, 126.9, 121.0, 116.9, 112.4, 71.0, 70.9, 70.8, 70.7, 70.6, 70.4, 70.0, 67.9, 63.5, 49.7, 43.8, 42.3, 41.1, 39.9, 37.4, 36.5, 29.8, 28.7, 27.4. HRMS (ESI$^+$) m/z[M+H]$^+$, calcd. for $C_{48}H_{69}ClN_5O_{10}S$: 942.4454, found: 942.4437. Purity by HPLC: 96%

General method of Boc deprotection:

A solution of compound **27** (0.01 mmol, 1 equiv., 6.5 mg) or **29** (0.01 mmol, 1 equiv., 7.8 mg) or **31** (0.01 mmol, 1 equiv., 9.4 mg) in dichloromethane (1 mL) was cooled at 0 °C and dropped trifluoroacetic acid (0.2 mmol, 20 equiv., 9.0 µL, 13.7 mg). The cooling bath was removed and the mixture was allowed to stir for 2 h at room temperature. Then, the solvent was evaporated and the crude residue was purified by preparative HPLC using eluents water and MeCN. The collected fractions were lyophilized.

(2-Aminophenyl)(4-(4-((3-chlorophenyl)sulfonyl)benzyl)-[1,4'-bipiperidin]-1'-yl)methanone TFA salt (**28**)

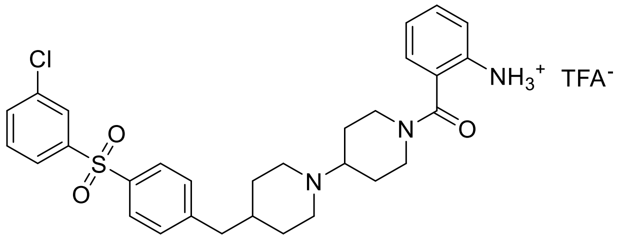

Yield: 5.2 mg (95%), colorless oil. ^{1}H NMR (DMSO-d_6, 300 MHz) δ 8.21 (s, 1H), 8.00 (t, J = 1.7 Hz, 1H), 7.93 (d, J = 8.3 Hz, 3H), 7.79 – 7.73 (m, 1H), 7.65 (t, J = 7.9 Hz, 1H), 7.43 (d, J = 8.3 Hz, 2H), 7.12 – 7.03 (m, 1H), 6.96 (dt, J = 10.2, 5.1 Hz, 1H), 6.70 (d, J = 8.1 Hz, 1H), 6.55 (t, J = 7.4 Hz, 1H), 5.15 (br, 4H), 4.04 (br, 1H), 3.00 (d, J = 11.1 Hz, 2H), 2.91 – 2.71 (m, 3H), 2.59 (d, J = 6.4 Hz, 2H), 2.35 (t, J = 11.1 Hz, 2H), 1.81 (d, J = 11.6 Hz, 2H), 1.68 – 1.12 (m, 8H). ^{13}C NMR (DMSO-d_6, 75 MHz) δ 168.4, 163.6, 147.2, 145.7, 143.3, 137.9, 134.3, 133.6, 131.8, 130.4, 129.9, 127.6, 127.4, 126.8, 126.1, 119.5, 115.5, 61.5, 48.5, 41.5, 36.2, 30.6, 27.2. HRMS (APCI$^+$) m/z[M+H]$^+$, calcd. for $C_{30}H_{35}ClN_3O_3S$: 552.2082, found: 552.2088. Purity by HPLC: 99%

(2-((2-(2-(2-Aminoethoxy)ethoxy)ethyl)amino)phenyl)(4-(4-((3-chlorophenyl)sulfonyl)-benzyl)-[1,4'-bipiperidin]-1'-yl)methanone TFA salt (30)

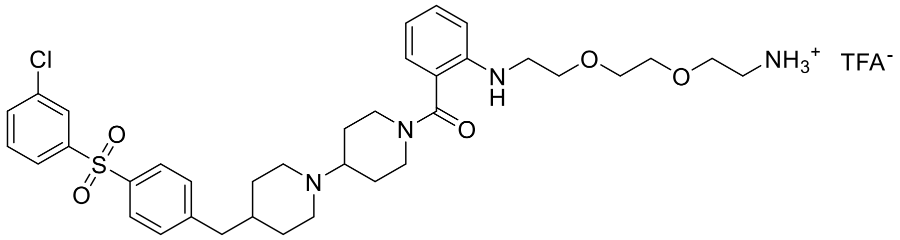

Yield: 6.3 mg (92%), yellow oil. ^{1}H NMR (DMSO-d_6, 300 MHz) δ 8.77 (br, 2H), 8.00 (s, 1H), 7.93 (t, J = 7.5 Hz, 5H), 7.76 (d, J = 8.1 Hz, 1H), 7.65 (t, J = 7.9 Hz, 1H), 7.45 (d, J = 8.2 Hz, 2H), 7.22 (t, J = 7.6 Hz, 1H), 7.05 (d, J = 7.2 Hz, 1H), 6.72 (d, J = 8.3 Hz, 1H), 6.62 (t, J = 7.3 Hz, 1H), 5.73 (s, 1H), 4.02 (br, 1H), 3.65 – 3.50 (m, 8H), 3.39 (d, J = 10.6 Hz, 3H), 3.30 – 3.16 (m, 2H), 3.05 – 2.75 (m, 5H), 2.62 (d, J = 6.1 Hz, 2H), 2.10 – 1.94 (m, 4H), 1.89 – 1.33 (m, 6H). ^{13}C NMR (DMSO-d_6, 75 MHz) δ 168.6, 159.1, 158.7, 158.2, 157.7, 146.5, 146.3, 145.9, 143.3, 138.2, 134.4, 133.7, 131.9, 130.7, 130.5, 127.8, 127.6, 126.8, 126.1, 119.5, 115.5, 111.2, 69.7, 69.5, 68.7, 66.7, 62.5, 54.9, 48.6, 42.4, 41.0, 34.6, 30.7, 28.7, 26.0. HRMS (ESI$^+$) m/z[M+H]$^+$, calcd. for $C_{36}H_{48}ClN_4O_5S$: 683.3034, found: 683.3043. Purity by HPLC: > 99%

3-(2-(2-Aminoethoxy)ethoxy)-N-(2-(2-(2-((2-(4-(4-((3-chlorophenyl)sulfonyl)benzyl)-[1,4'-bipiperidine]-1'-carbonyl)phenyl)amino)ethoxy)ethoxy)ethyl)propenamide TFA salt (32)

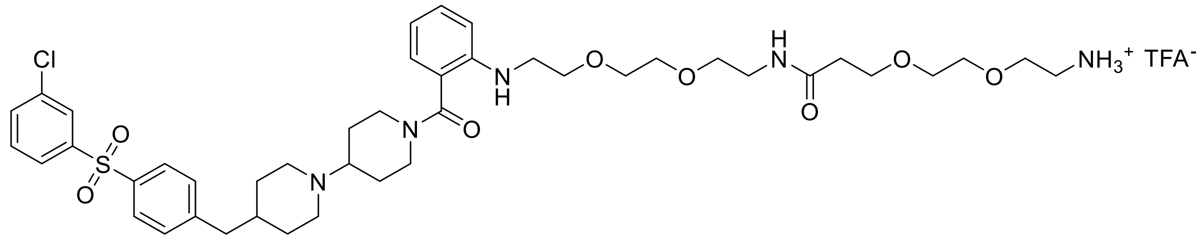

Yield: 8.2 mg (97%), colorless oil. ^{1}H NMR (DMSO-d_6, 300 MHz) δ 12.50 (br, 3H), 9.61 (br, 1H), 7.98 (s, 1H), 7.92 (t, J = 7.2 Hz, 6H), 7.73 (d, J = 7.9 Hz, 1H), 7.63 (t, J = 7.9 Hz, 1H), 7.44 (d, J = 8.1 Hz, 2H), 7.21 (t, J = 7.4 Hz, 1H), 7.06 (d, J = 7.3 Hz, 1H), 6.73 (d, J = 8.3 Hz, 1H), 6.68 – 6.59 (m, 1H), 5.69 (s, 1H), 4.25 – 3.97 (m, 1H), 3.66 – 3.46 (m, 15H), 3.41 (t, J = 10.0 Hz, 5H), 3.28 – 3.15 (m, 4H), 3.03 – 2.75 (m, 6H), 2.61 (d, J = 6.0 Hz, 2H), 2.38 – 2.24 (m, 2H), 2.14 – 1.93 (m, 2H), 1.90 – 1.36 (m, 5H), 1.28 (q, J = 7.0 Hz, 1H). ^{13}C NMR (DMSO-d_6, 75 MHz) δ 170.5, 168.7, 159.5, 159.0, 158.5, 158.0, 146.6, 145.7, 143.5, 138.4, 134.5, 133.8, 131.9, 130.8, 130.6, 128.0, 127.9, 127.8, 127.0, 126.2, 121.5, 120.0, 117.7, 115.9, 113.8, 111.6, 110.0, 69.7, 69.5, 69.3, 68.8, 66.9, 66.8, 65.0, 62.7, 56.2, 54.9, 48.8, 42.8, 41.1, 38.8, 36.1, 34.7, 30.6, 28.8, 26.1, 18.6, 13.5. HRMS (ESI$^+$) m/z[M+H]$^+$, calcd. for $C_{43}H_{61}ClN_5O_8S$: 842.3929, found: 842.3953. Purity by HPLC: > 99%

***N*-(1-((2-(4-(4-((3-Chlorophenyl)sulfonyl)benzyl)-[1,4'-bipiperidine]-1'-carbonyl)-phenyl)amino)-10-oxo-3,6,13,16-tetraoxa-9-azaoctadecan-18-yl)-2',7'-difluoro-3',6'-dihydroxy-3-oxo-3*H*-spiro[isobenzofuran-1,9'-xanthene]-5-carboxamide** (**33**)

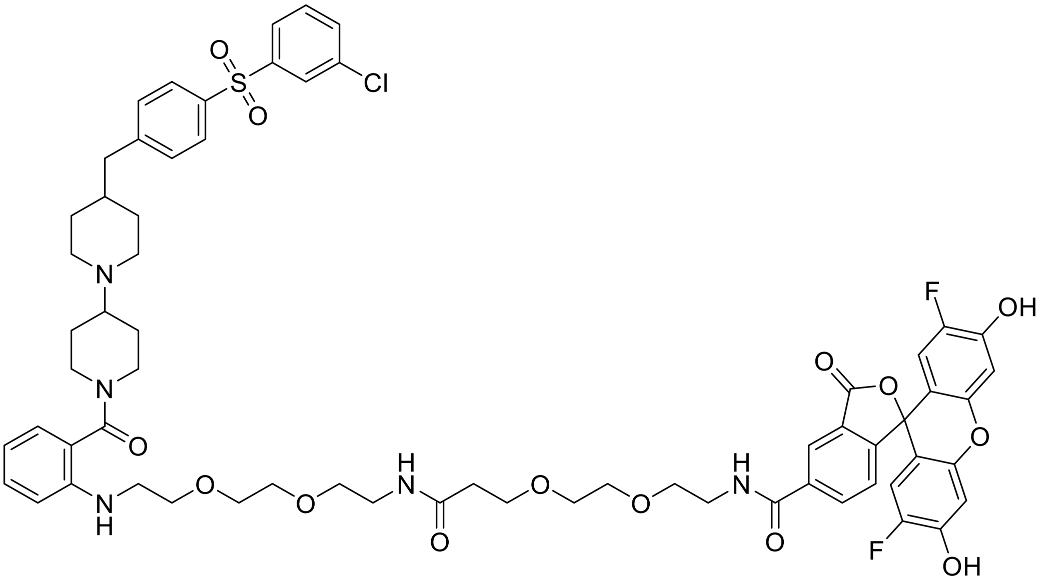

To a stirred solution of **32** (0.005 mmol, 1 equiv., 4.2 mg), DIPEA (0.01 mmol, 2 equiv., 2 µL) in DMSO (0.5 mL) Oregon Green 488 succinimidyl ester (0.005 mmol, 1 equiv., 2.5 mg) was

added under argon atmosphere and the reaction mixture was stirred for 30 min. at room temperature in darkness. It was purified by preparative HPLC using eluents A (0.1% HCOOH in MeCN) and B (0.1% HCOOH in H_2O). The collected fractions were lyophilized.

Yield: 2.4 mg (40%), orange solid. ^{1}H NMR (DMSO-d_6, 500 MHz) δ 8.59 (t, J = 5.4 Hz, 1H), 8.49 (br, 2H), 8.39 (br, J = 9.3 Hz, 1H), 8.04 – 7.97 (m, 2H), 7.93 – 7.88 (m, 3H), 7.82 (d, J = 7.8 Hz, 1H), 7.76 (d, J = 8.0 Hz, 1H), 7.67 – 7.61 (m, 1H), 7.41 (d, J = 8.3 Hz, 2H), 7.18 (t, J = 7.8 Hz, 1H), 7.12 (d, J = 7.7 Hz, 1H), 6.99 (d, J = 7.4 Hz, 1H), 6.72 (br, 1H), 6.68 (d, J = 8.3 Hz, 1H), 6.59 (t, J = 7.4 Hz, 1H), 6.27 (d, J = 12.6 Hz, 2H), 6.15 (d, J = 7.8 Hz, 2H), 5.14 (t, J = 5.5 Hz, 1H), 3.60 (t, J = 6.5 Hz, 2H), 3.55 – 3.52 (m, 4H), 3.51 – 3.47 (m, 6H), 3.45 (d, J = 5.8 Hz, 2H), 3.21 – 3.15 (m, 4H), 2.78 (d, J = 11.1 Hz, 3H), 2.64 – 2.62 (m, 2H), 2.36 (s, 2H), 2.32 (t, J = 6.3 Hz, 2H), 2.05 – 1.99 (m, 2H), 1.68 (d, J = 11.2 Hz, 2H), 1.46 (d, J = 9.6 Hz, 3H), 1.39 – 1.20 (m, 4H), 1.18 – 0.98 (m, 6H). HRMS (ESI$^+$) m/z[M+H]$^+$, calcd. for $C_{64}H_{69}ClF_2N_5O_{14}S$: 1236.4218, found: 1236.4220. Purity by HPLC: 97%

HPLC-MS, ^{1}H and ^{13}C NMR Spectra

1-((4-Bromophenyl)sulfonyl)-3-chlorobenzene (14)

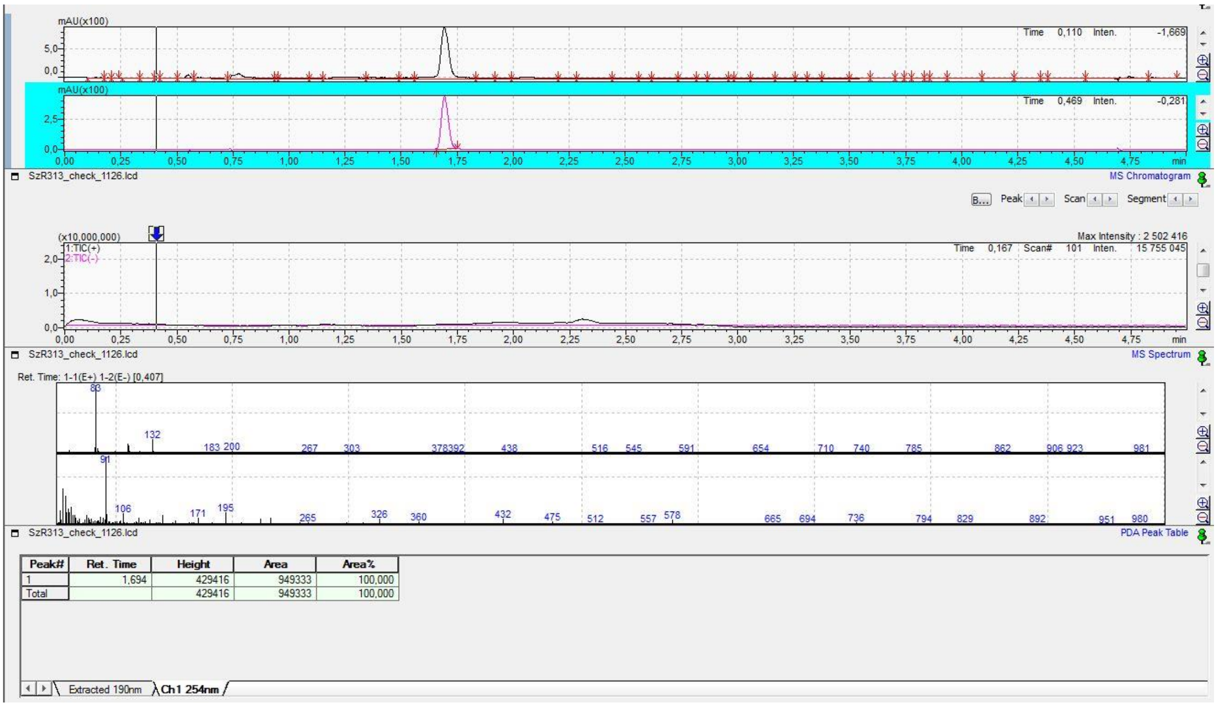

Peak#	Ret. Time	Height	Area	Area%
1	1,694	429416	949333	100,000
Total		429416	949333	100,000

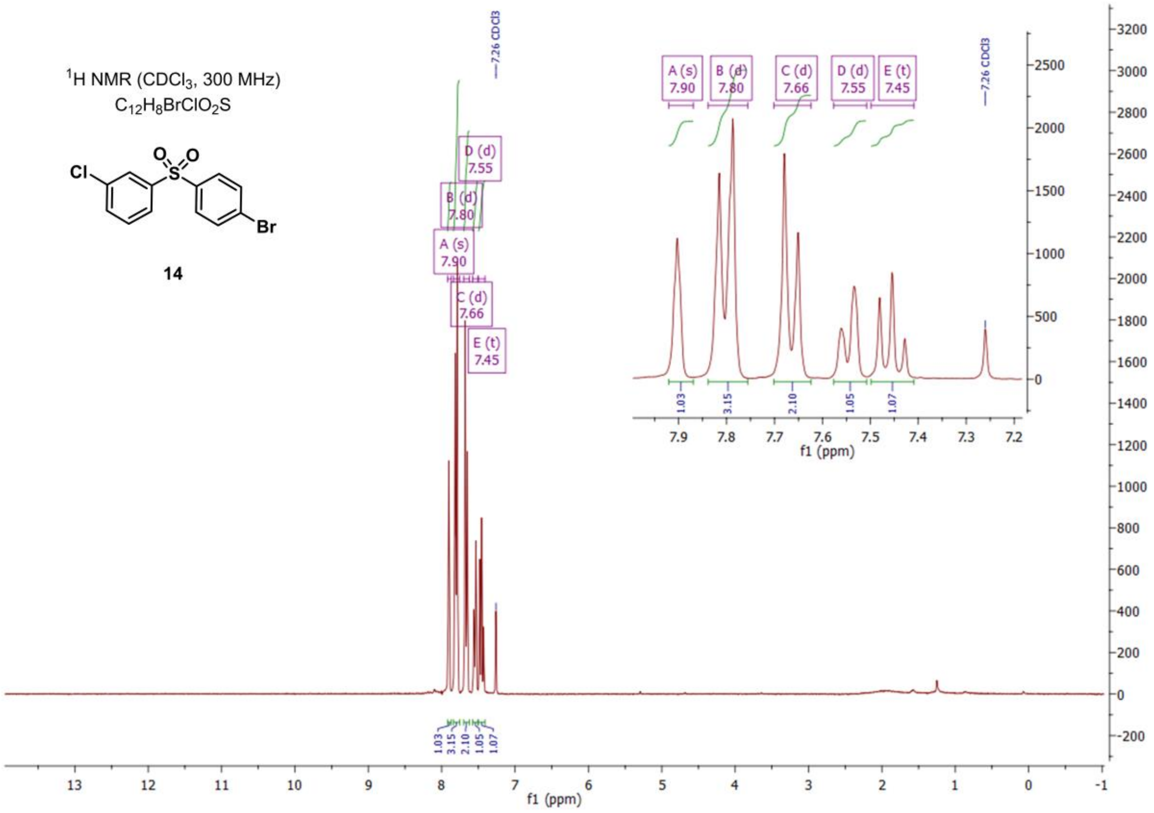

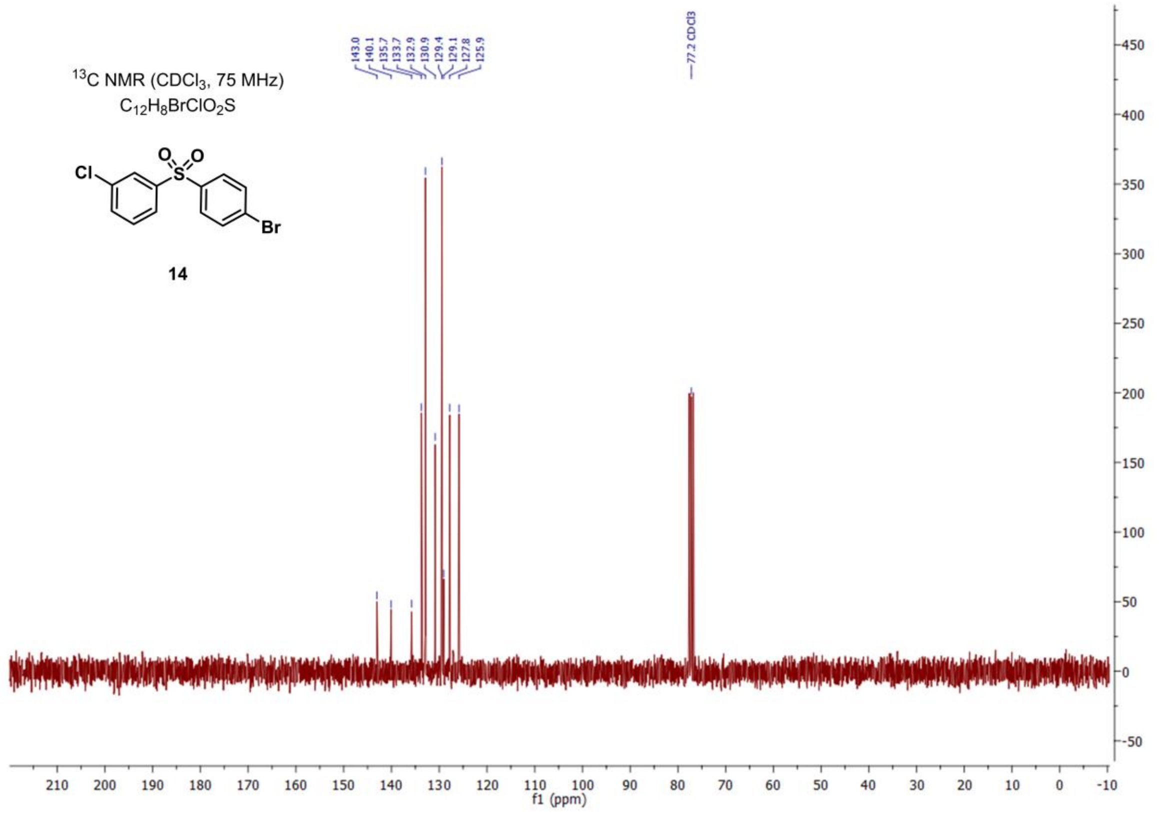

tert-Butyl 4-(4-((3-chlorophenyl)sulfonyl)benzyl)piperidine-1-carboxylate (17)

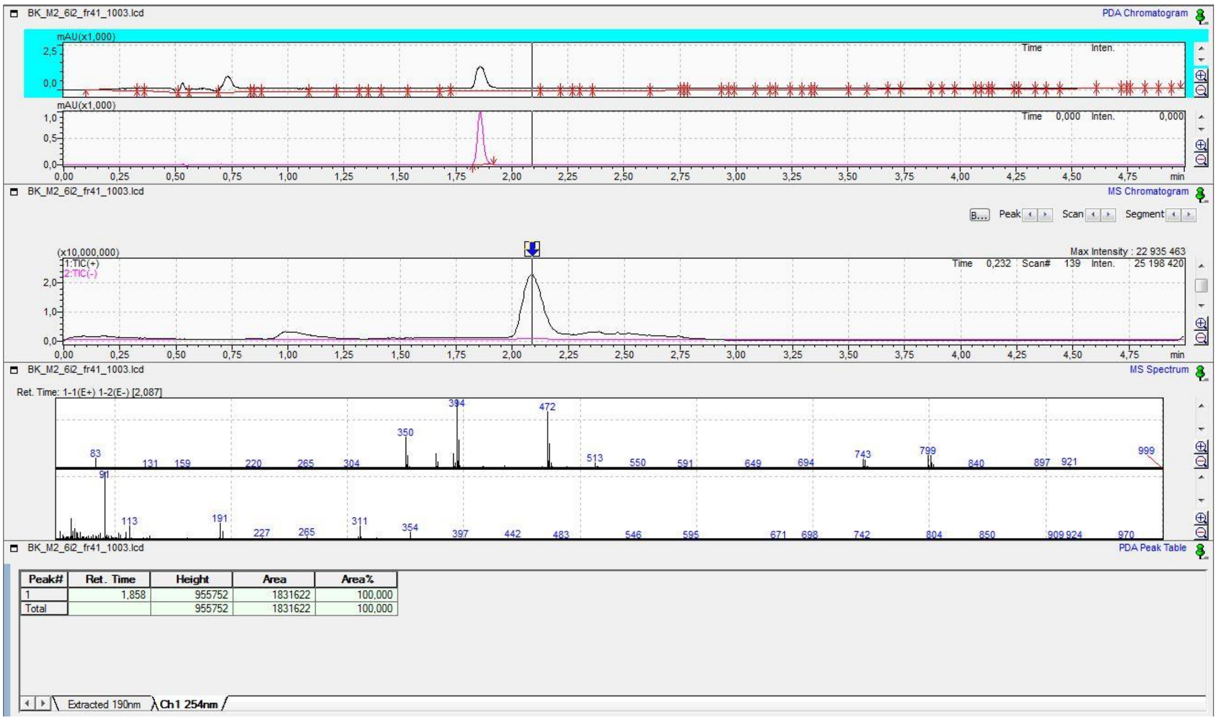
BK_M2_6i2_fr41_1003.lcd
PDA Chromatogram
mAU(x1,000)
MS Chromatogram
Max Intensity : 22 935 463
1:TIC(+)
2:TIC(-)
MS Spectrum
Ret. Time: 1-1(E+) 1-2(E-) [2,087]
PDA Peak Table
Peak#	Ret. Time	Height	Area	Area%
1	1,858	955752	1831622	100,000
Total		955752	1831622	100,000
Extracted 190nm
Ch1 254nm

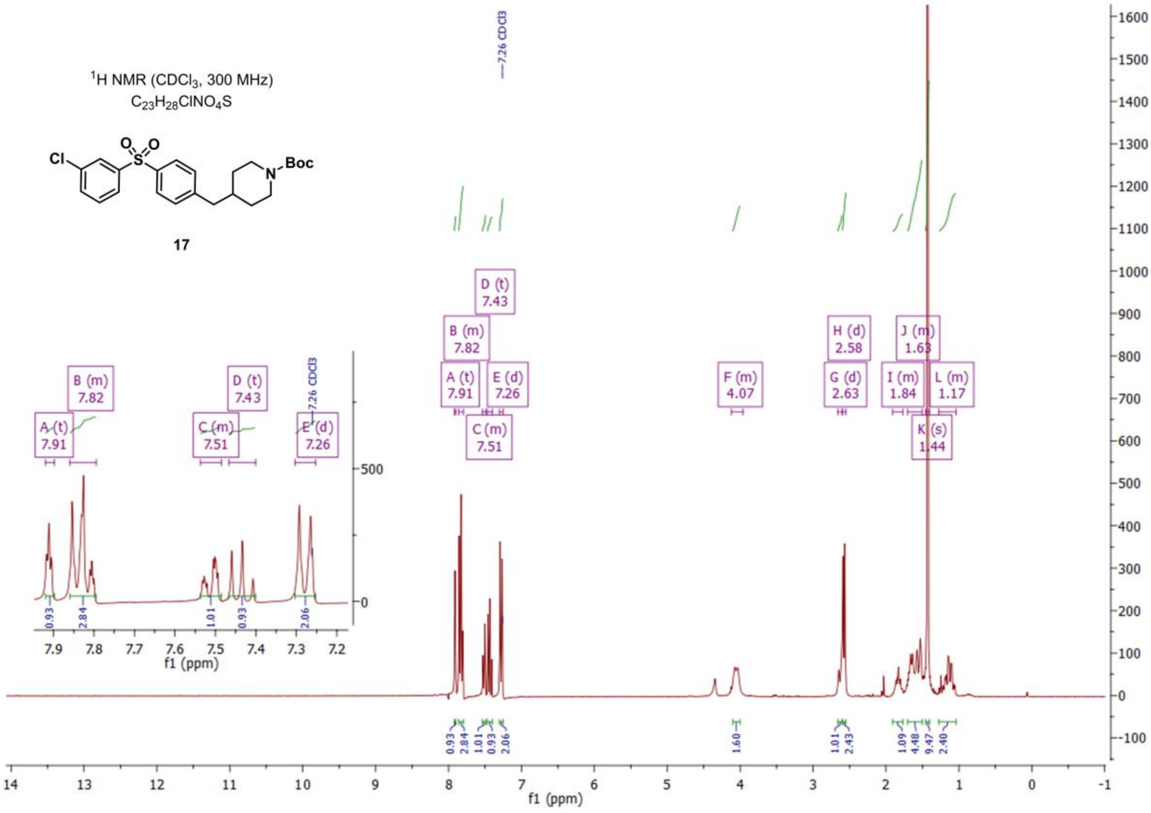
^{1}H NMR ($CDCl_3$, 300 MHz)
$C_{23}H_{28}ClNO_4S$
Cl
Boc
17
7.26 CDCl3
A (t) 7.91
B (m) 7.82
C (m) 7.51
D (t) 7.43
E (d) 7.26
F (m) 4.07
G (d) 2.63
H (d) 2.58
I (m) 1.84
J (m) 1.63
K (s) 1.44
L (m) 1.17
f1 (ppm)

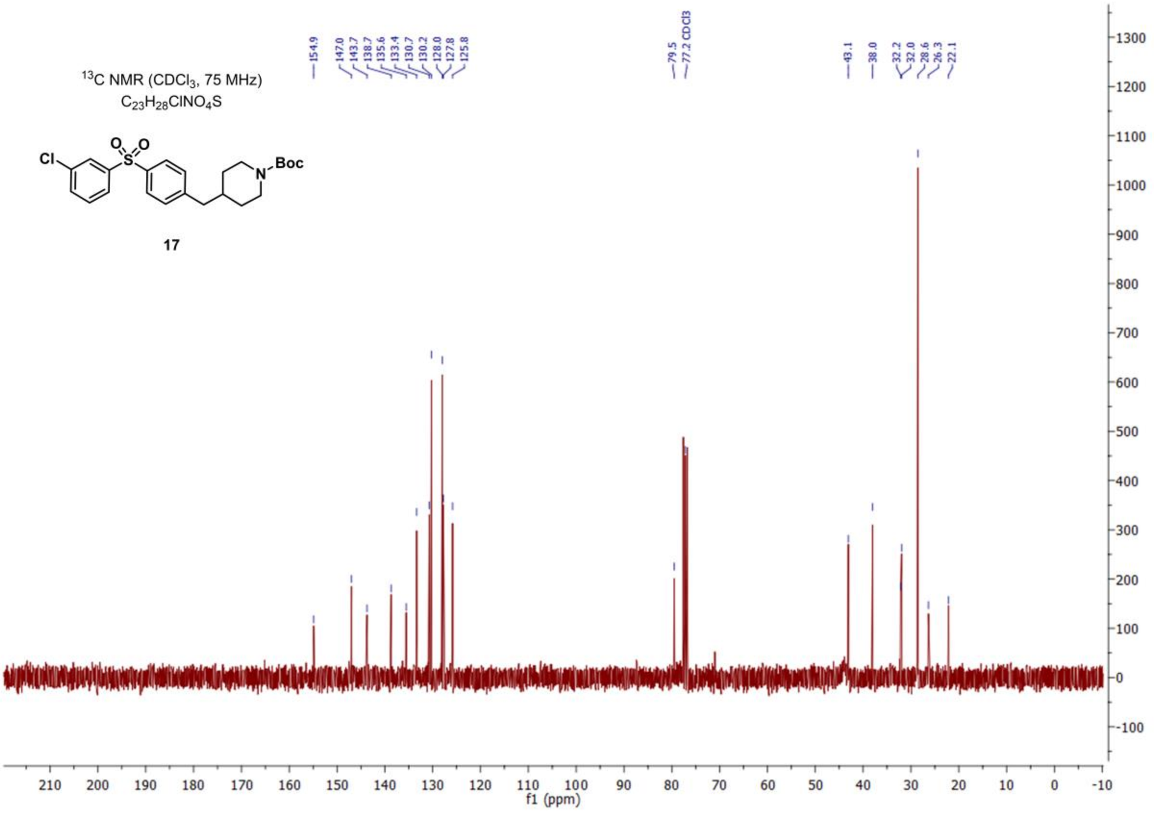

4-(4-((3-Chlorophenyl)sulfonyl)benzyl)piperidine TFA salt (18)

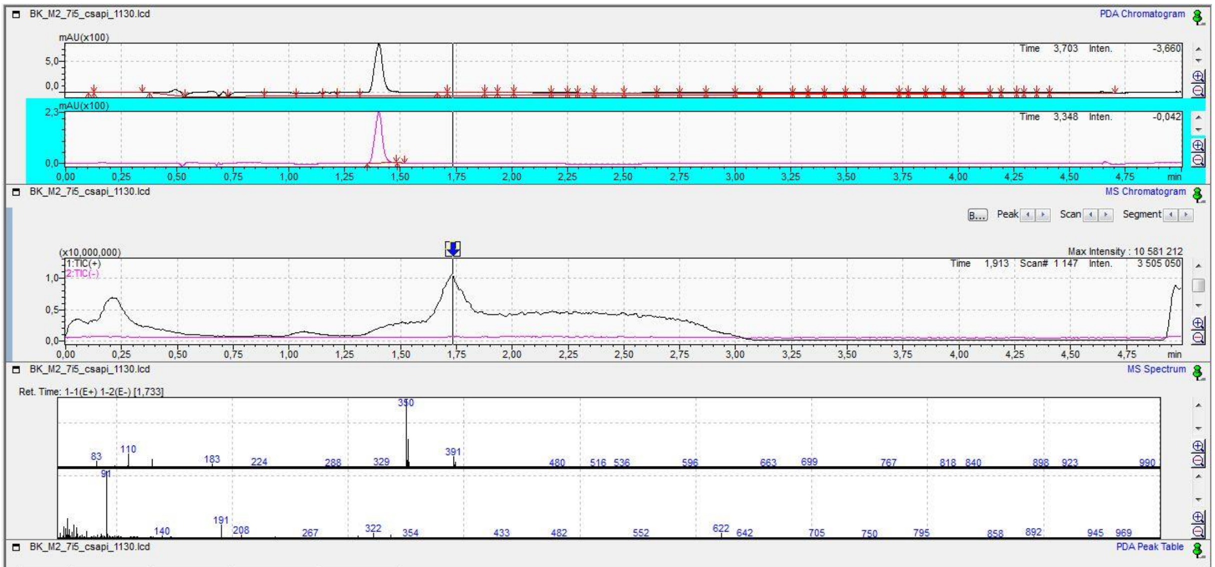

Peak#	Ret. Time	Height	Area	Area%
1	1,403	226017	537227	99,977
2	1,489	23	124	0,023
Total		226040	537350	100,000

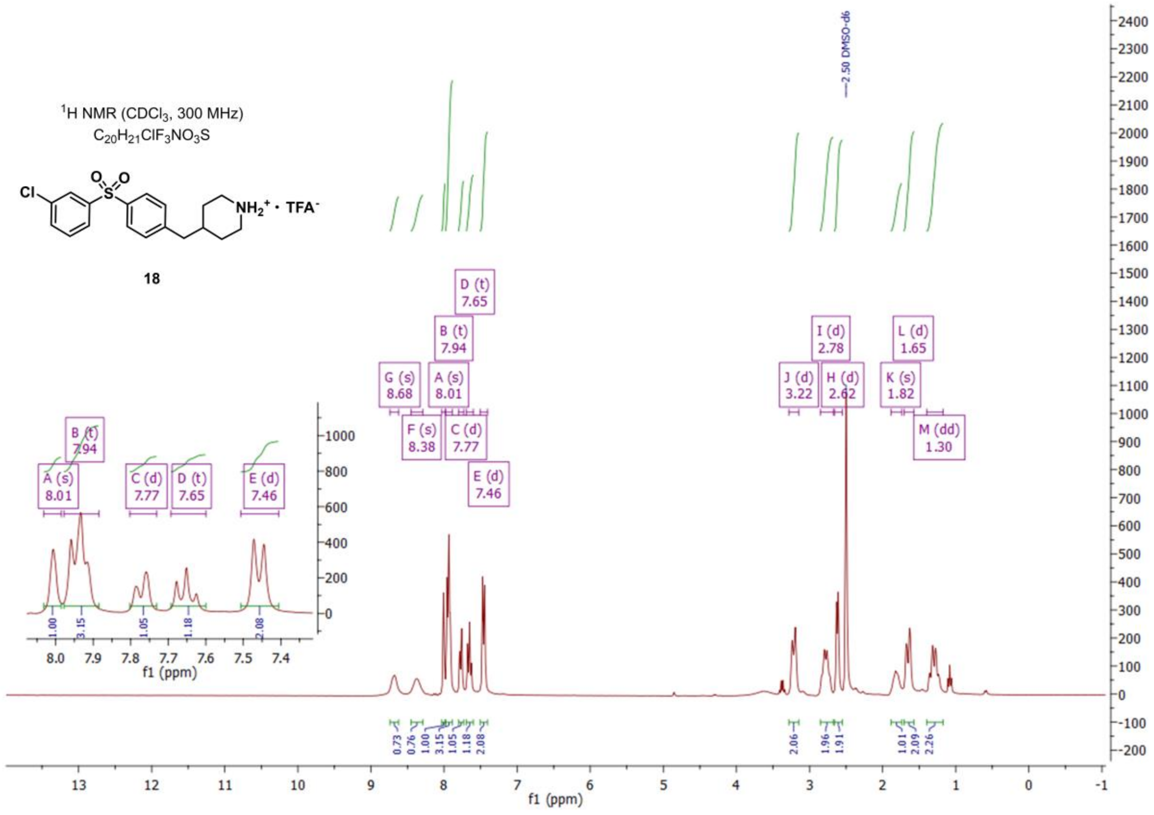
^{1}H NMR (CDCl$_3$, 300 MHz)
$C_{20}H_{21}ClF_3NO_3S$
18
2.50 DMSO-d6
f1 (ppm)

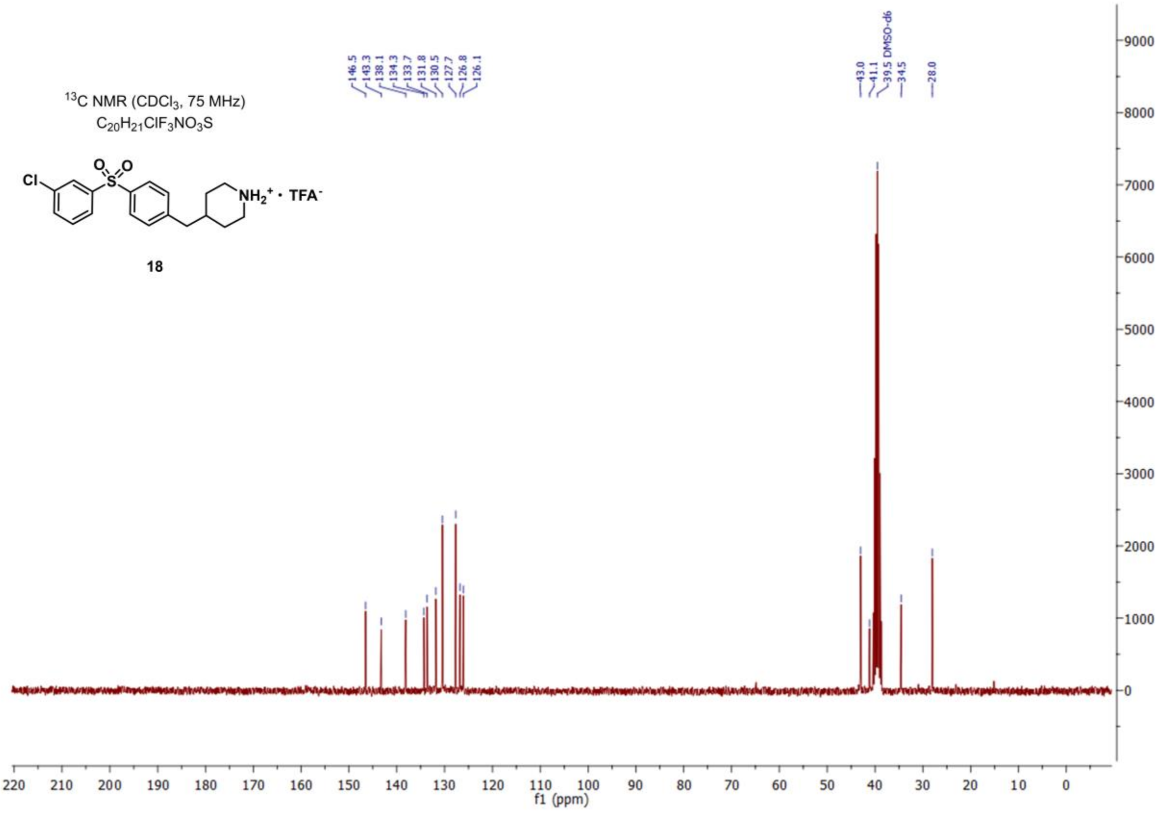
^{13}C NMR (CDCl$_3$, 75 MHz)
$C_{20}H_{21}ClF_3NO_3S$
18
146.5
143.3
138.1
134.3
133.7
131.8
130.5
127.7
126.8
126.1
43.0
41.1
39.5 DMSO-d6
34.5
28.0
f1 (ppm)

tert-Butyl 4-(4-((3-chlorophenyl)sulfonyl)benzyl)-[1,4'-bipiperidine]-1'-carboxylate (19)

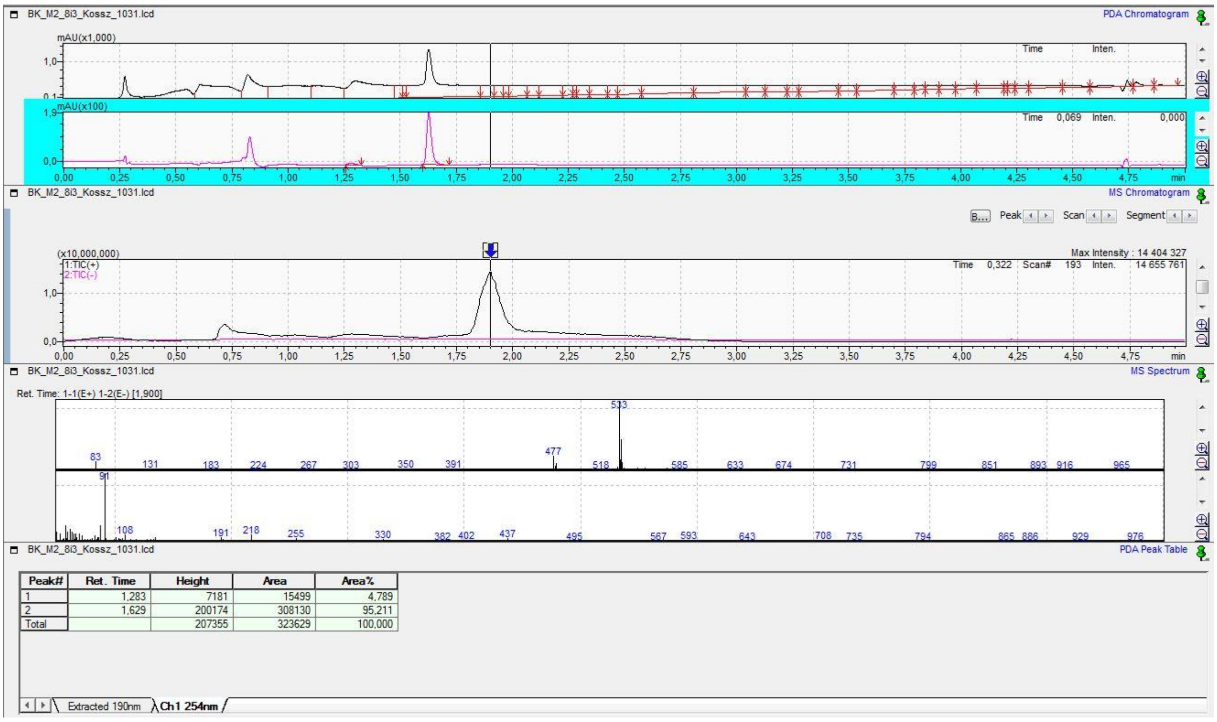

Peak#	Ret. Time	Height	Area	Area%
1	1,283	7181	15499	4,789
2	1,629	200174	308130	95,211
Total		207355	323629	100,000

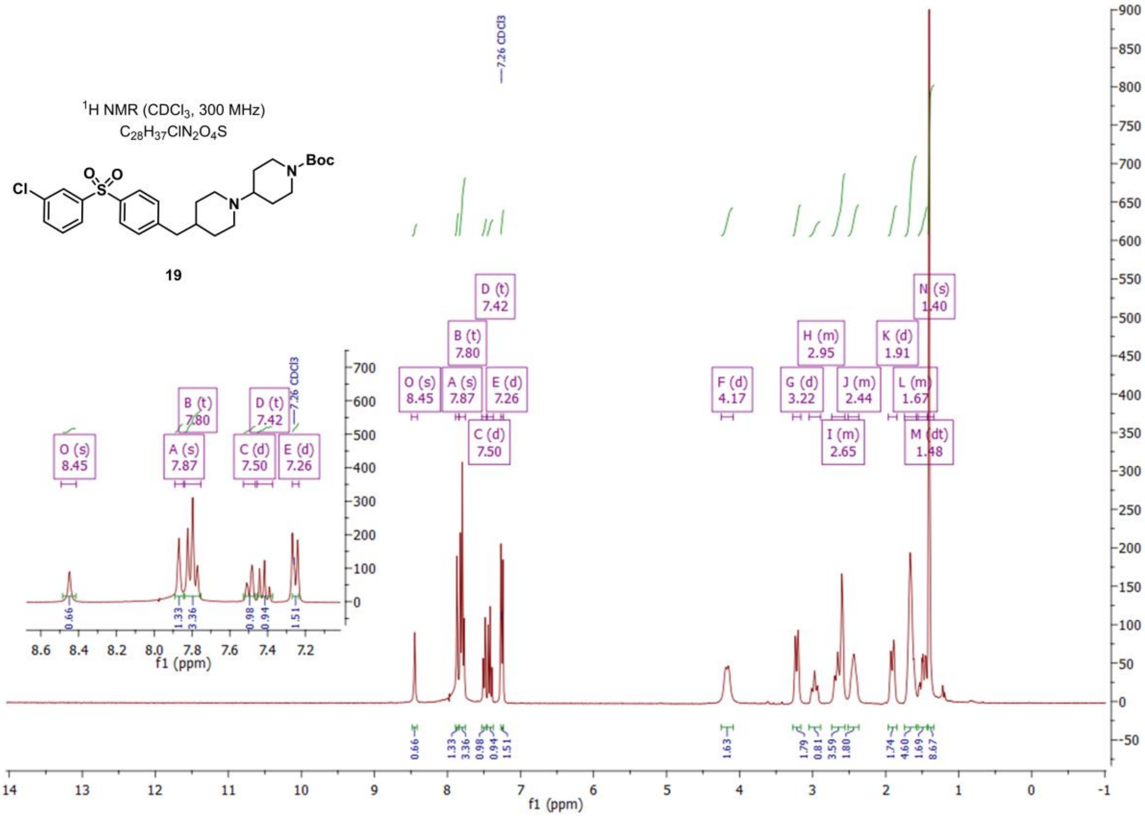

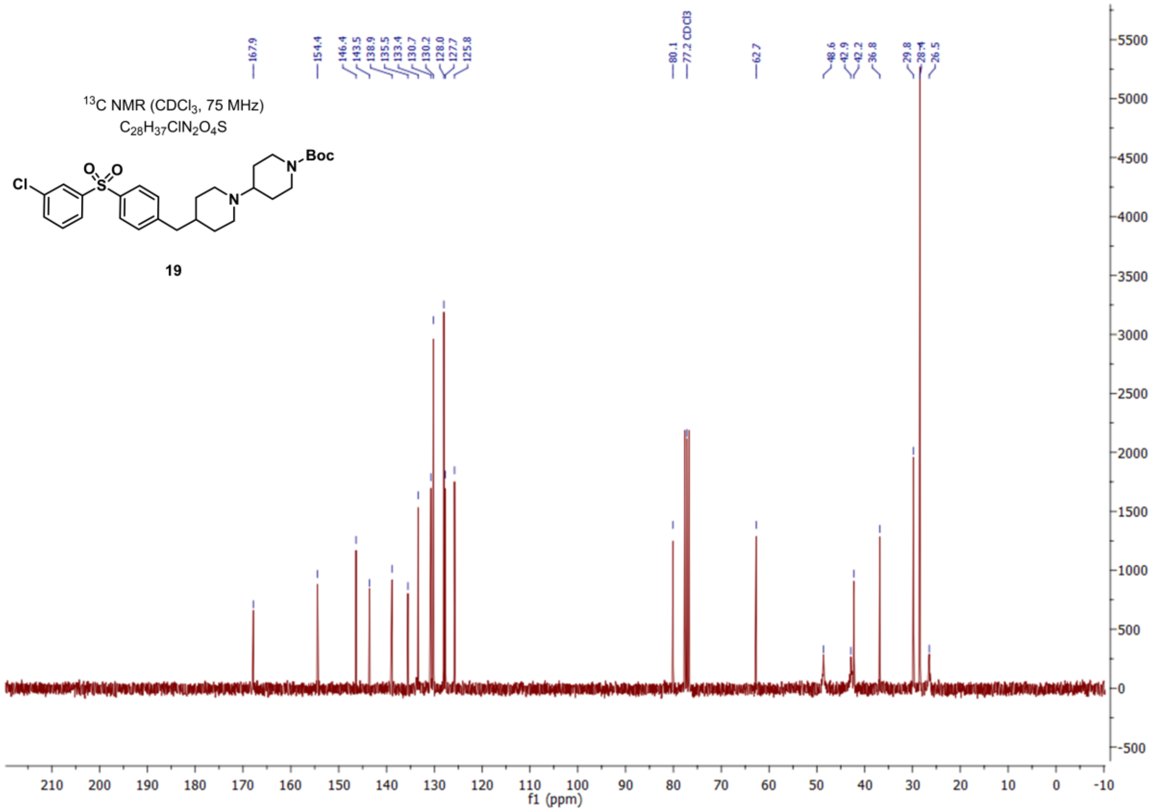

2-((2,2-Dimethyl-4-oxo-3,8,11-trioxa-5-azatridecan-13-yl)amino)benzoic acid (23)

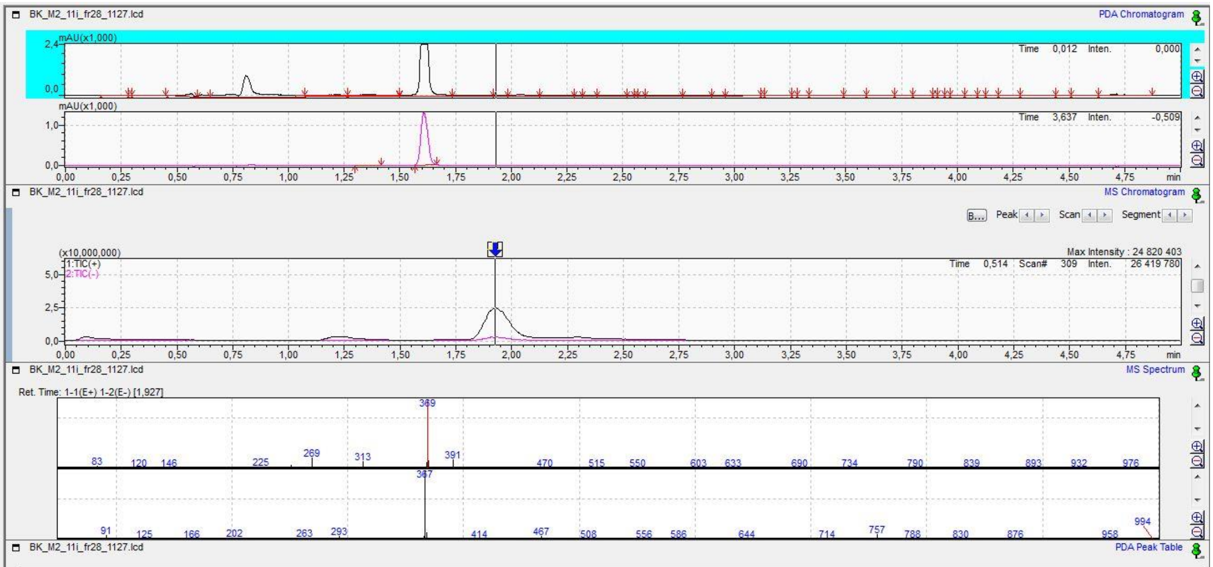

Peak#	Ret. Time	Height	Area	Area%
1	1,363	6508	22867	0,821
2	1,608	1249813	2763151	99,179
Total		1256321	2786017	100,000

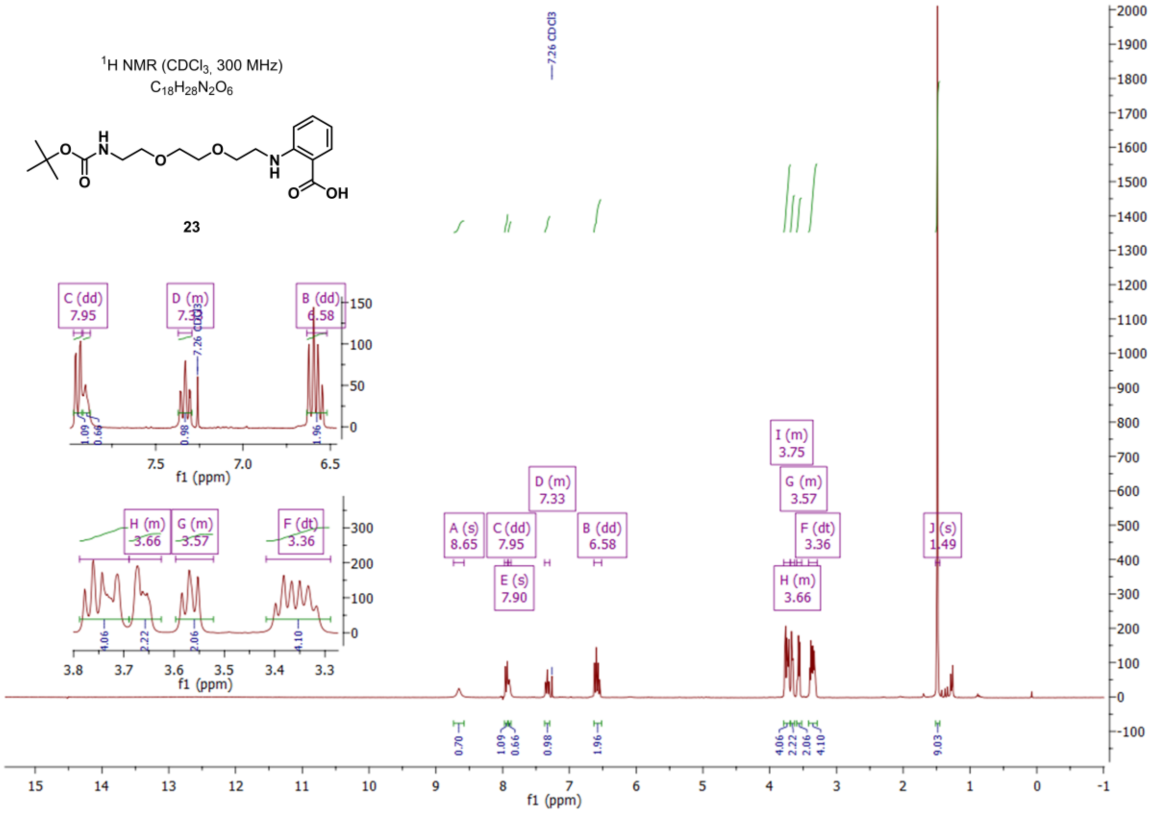

1H NMR (CDCl3, 300 MHz)
C18H28N2O6
23
7.26 CDCl3
A (s) 8.65
C (dd) 7.95
E (s) 7.90
D (m) 7.33
B (dd) 6.58
I (m) 3.75
H (m) 3.66
G (m) 3.57
F (dt) 3.36
J (s) 1.49
f1 (ppm)

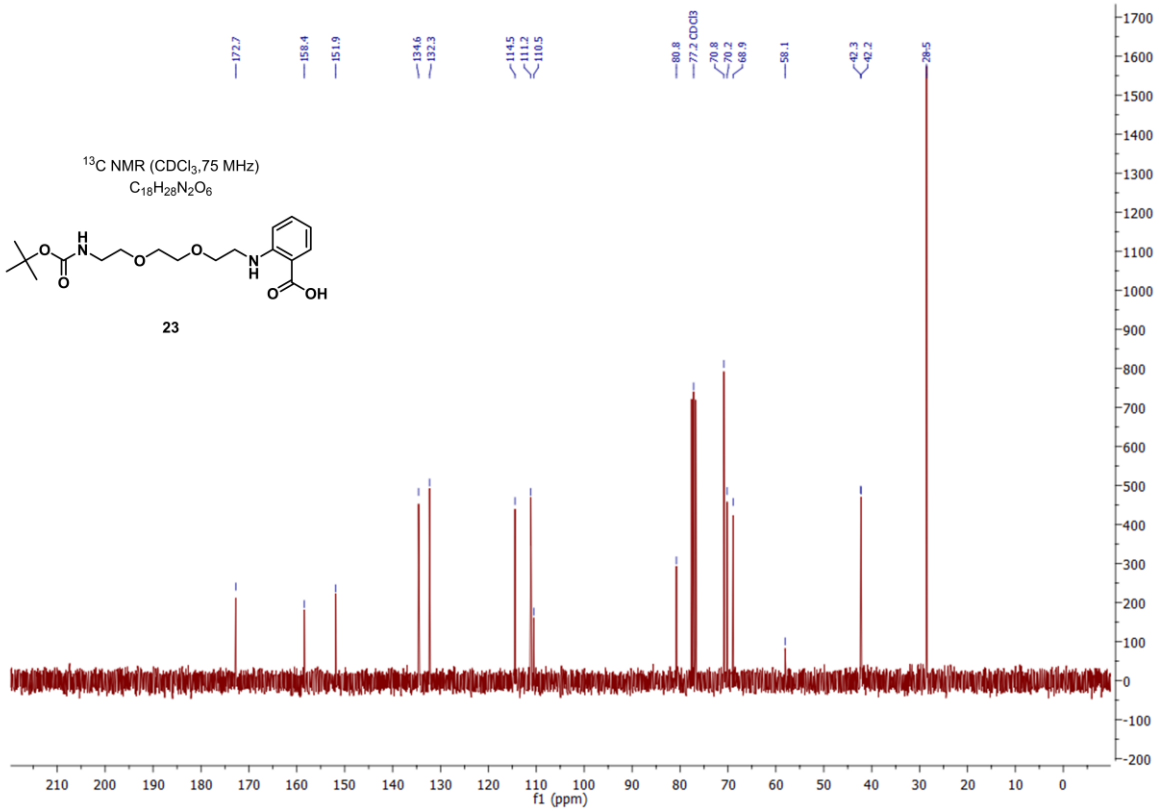

13C NMR (CDCl3,75 MHz)
C18H28N2O6
23
172.7
158.4
151.9
134.6
132.3
114.5
111.2
110.5
80.8
77.2 CDCl3
70.8
70.2
68.9
58.1
42.3
42.2
28.5
f1 (ppm)

2-((2-(2-(2-Aminoethoxy)ethoxy)ethyl)amino)benzoic acid TFA salt (24)

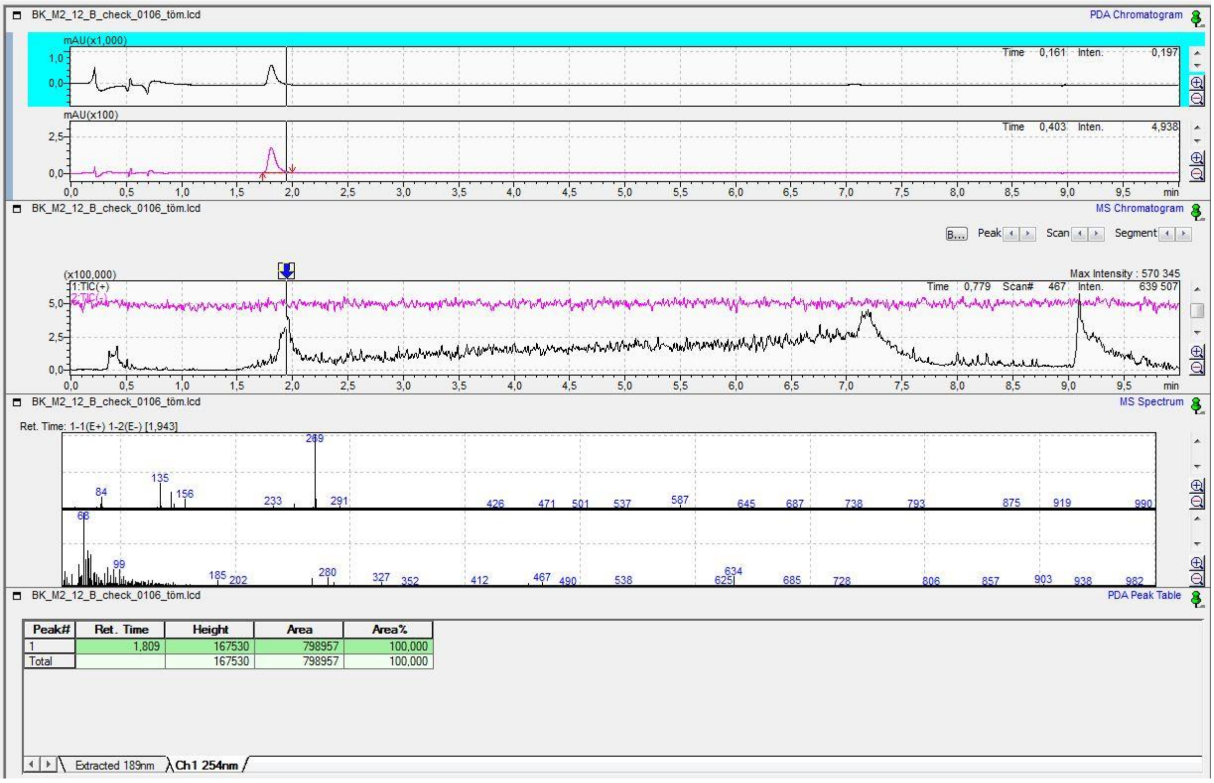

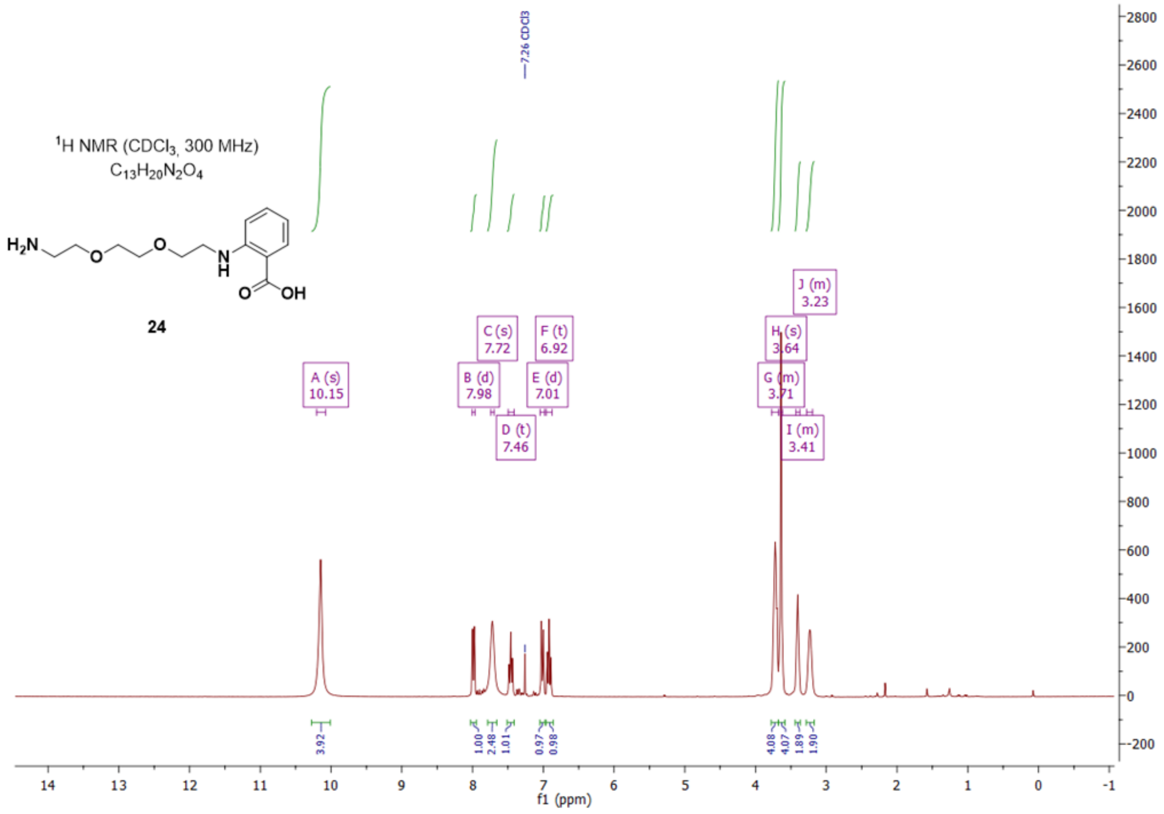

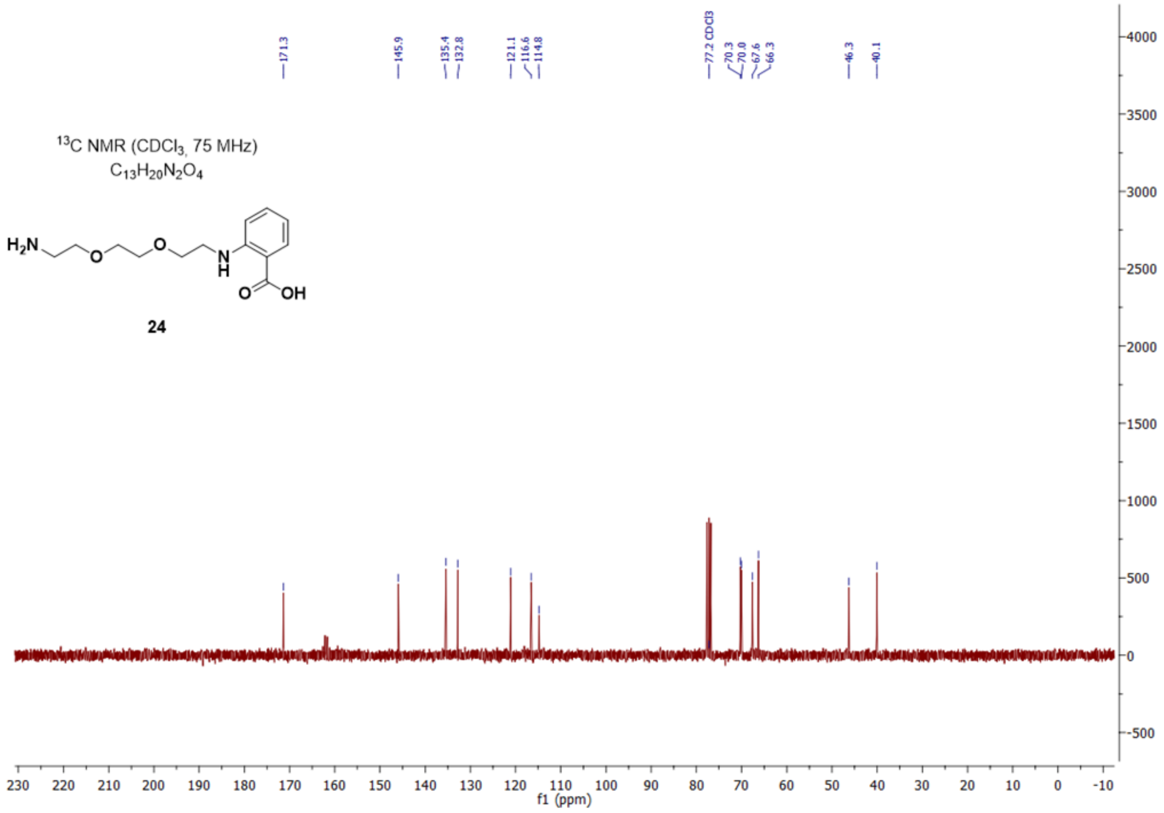

2-((2,2-Dimethyl-4,14-dioxo-3,8,11,18,21-pentaoxa-5,15-diazatricosan-23-yl)amino)-benzoic acid (25)

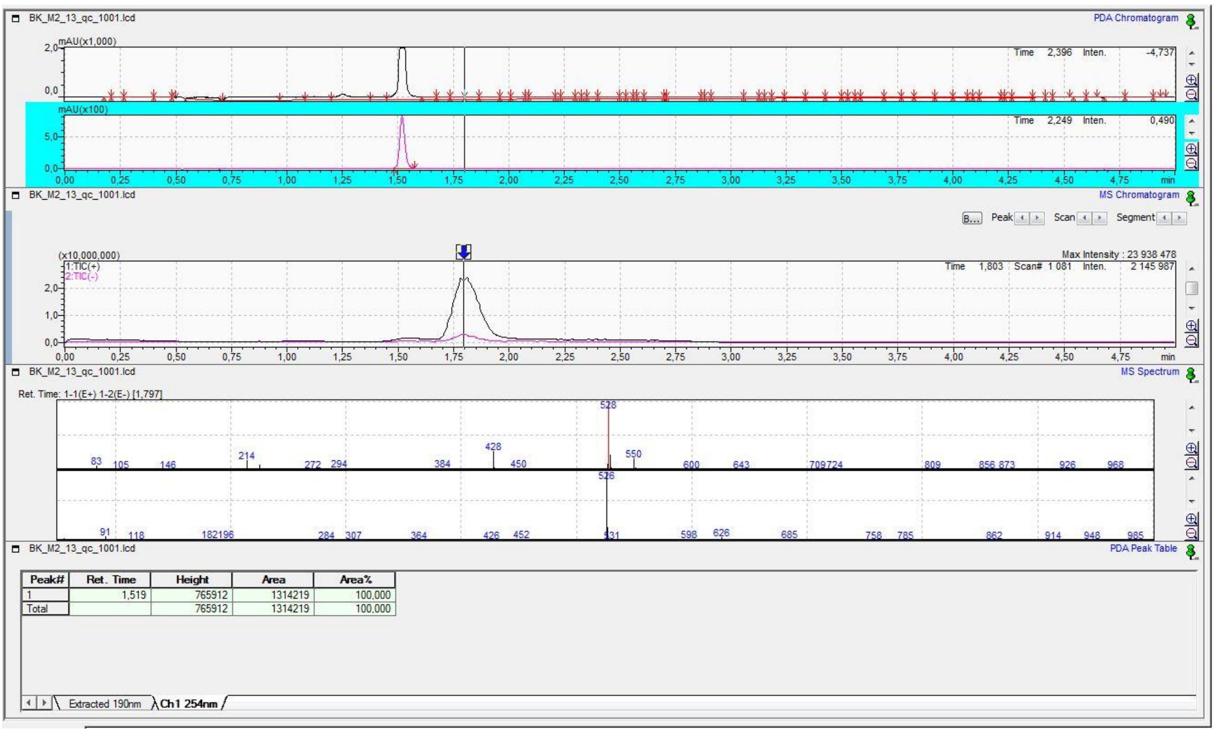

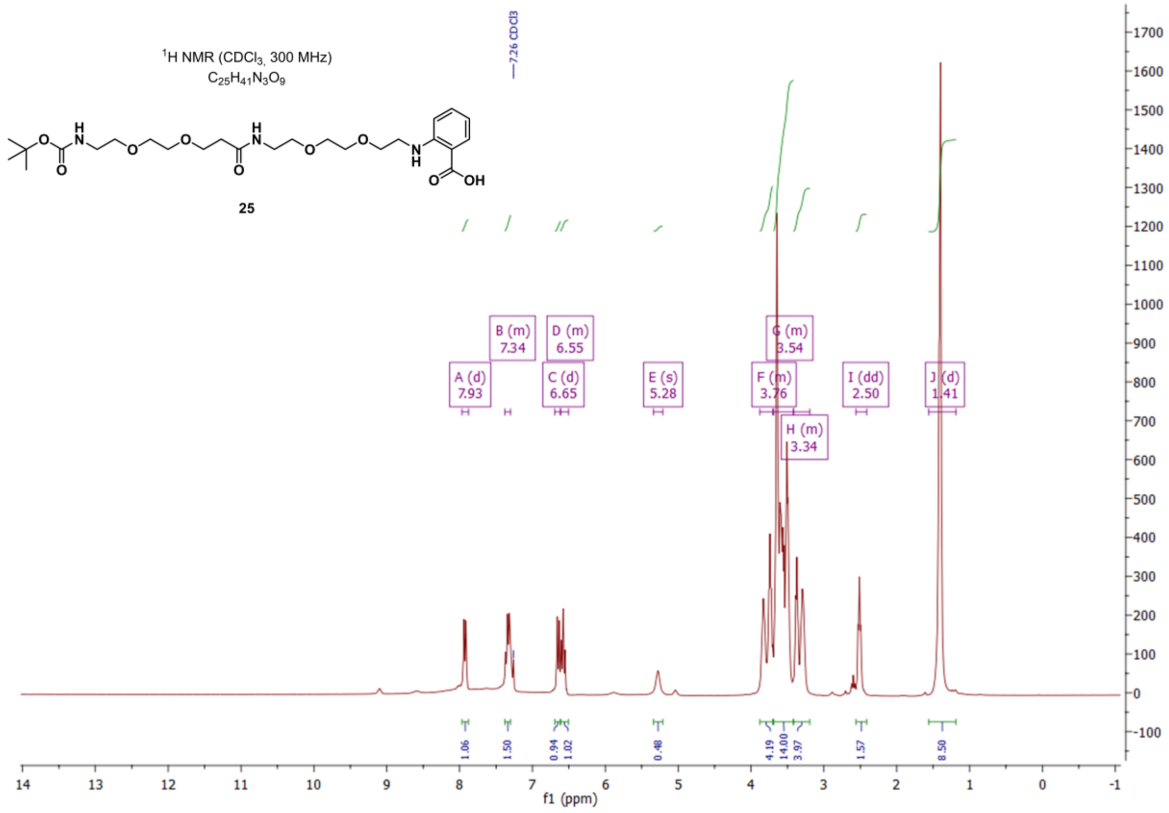
1H NMR (CDCl3, 300 MHz)
C25H41N3O9
25
f1 (ppm)

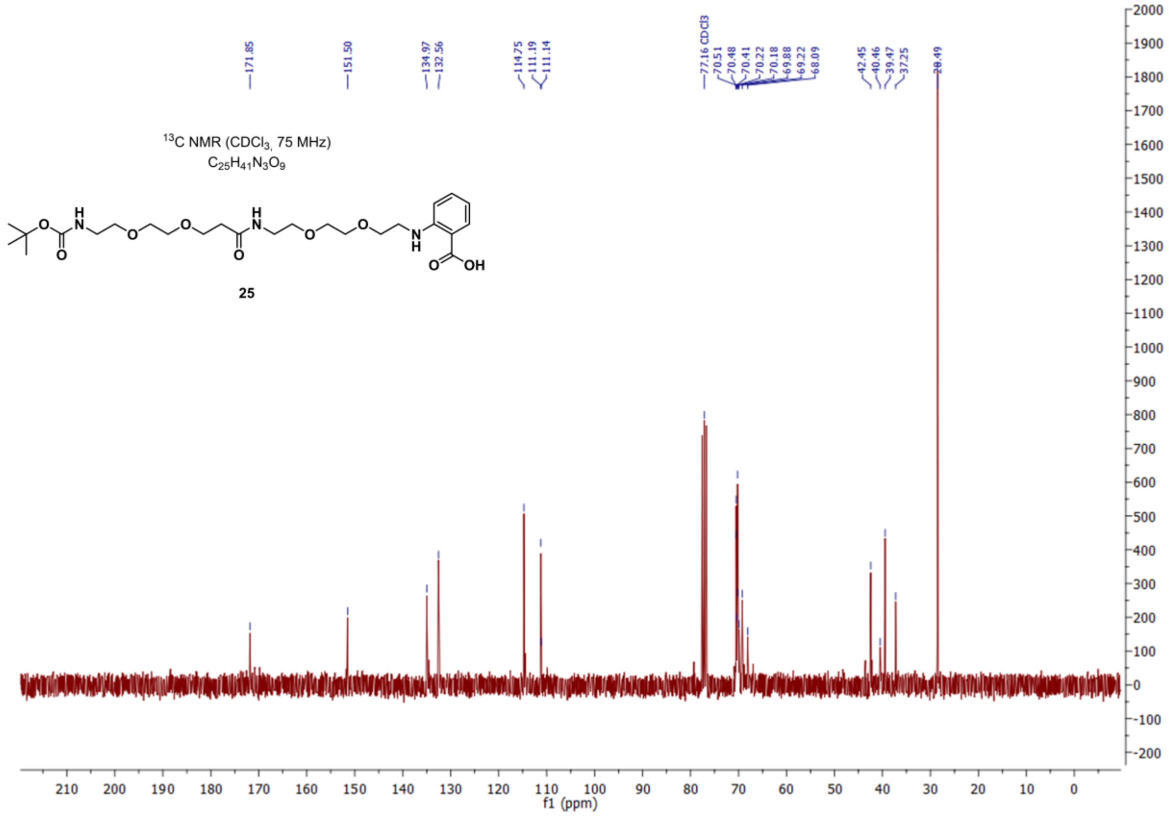
13C NMR (CDCl3, 75 MHz)
C25H41N3O9
25
f1 (ppm)

tert-Butyl (2-(4-(4-((3-chlorophenyl)sulfonyl)benzyl)-[1,4'-bipiperidine]-1'-carbonyl)-phenyl)carbamate (27)

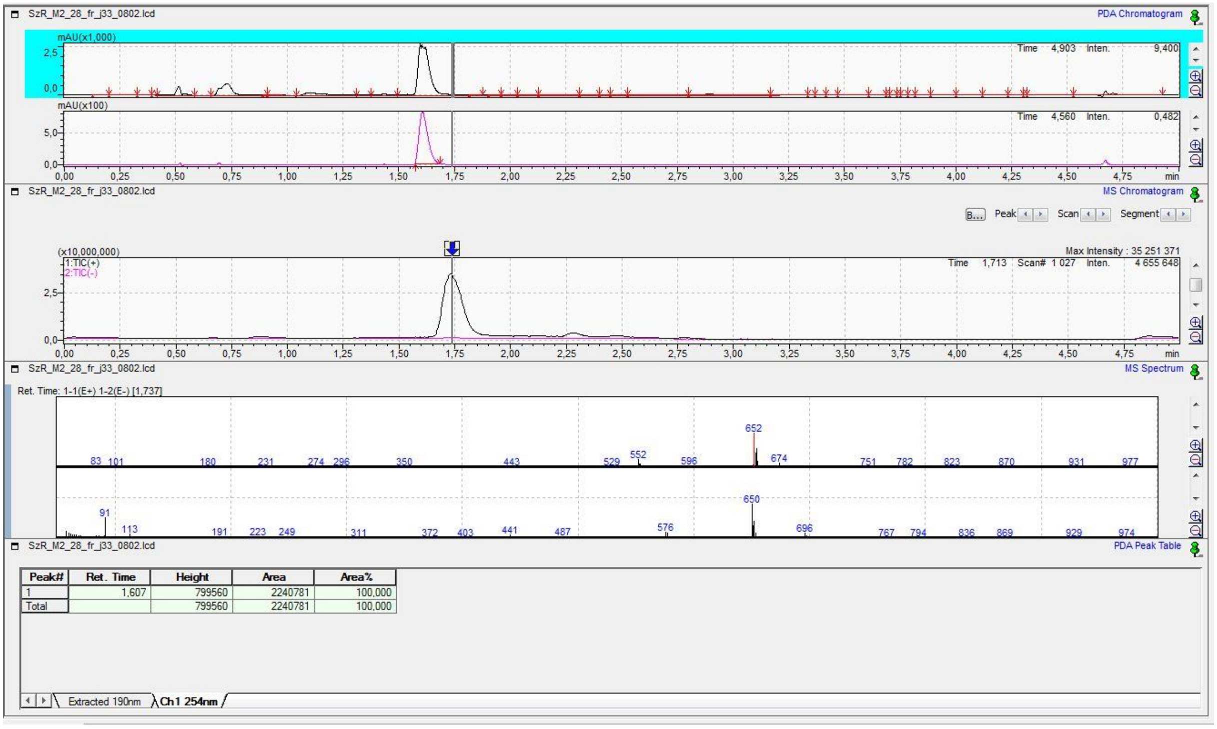

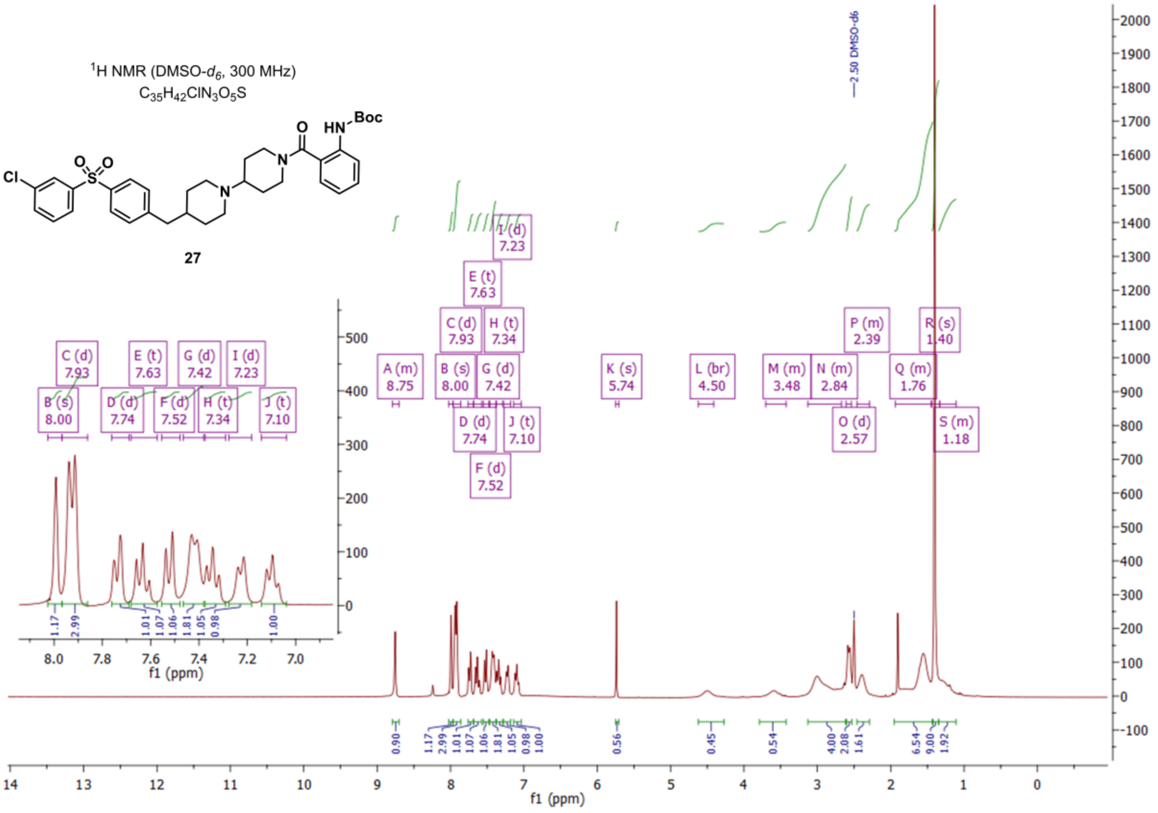

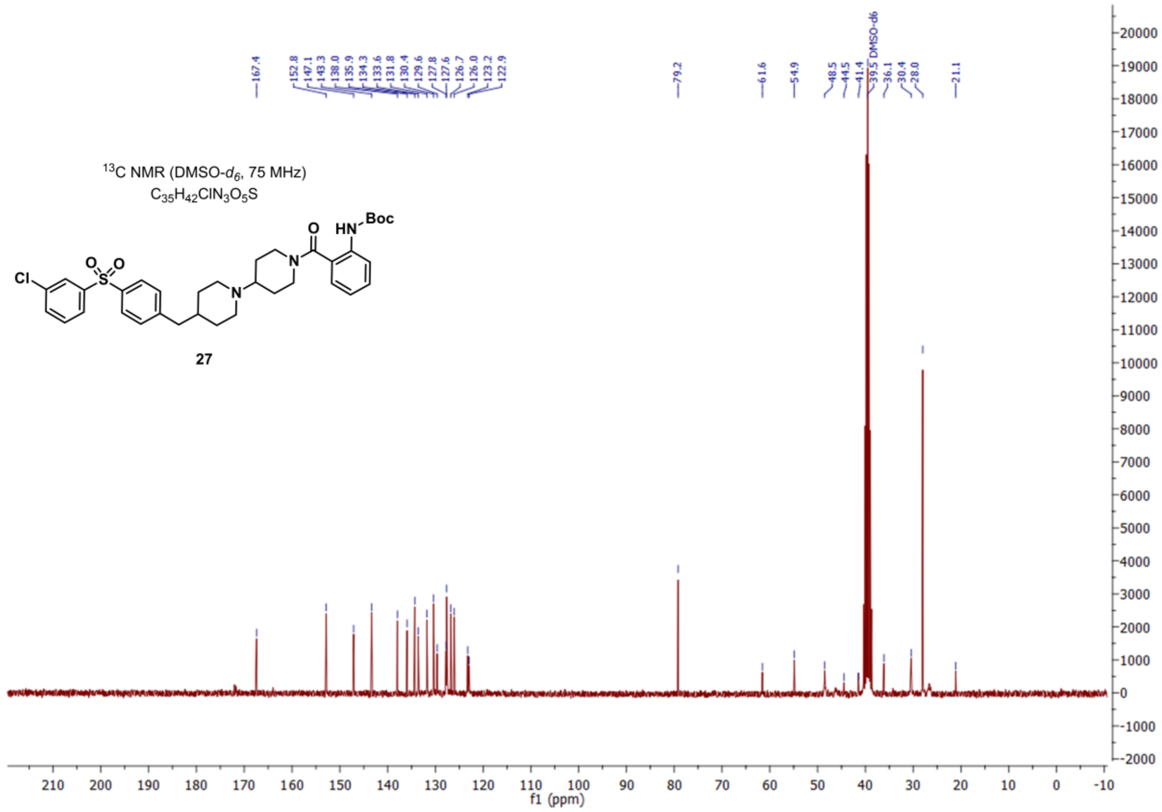

(2-Aminophenyl)(4-(4-((3-chlorophenyl)sulfonyl)benzyl)-[1,4'-bipiperidin]-1'-yl)methanone TFA salt (28)

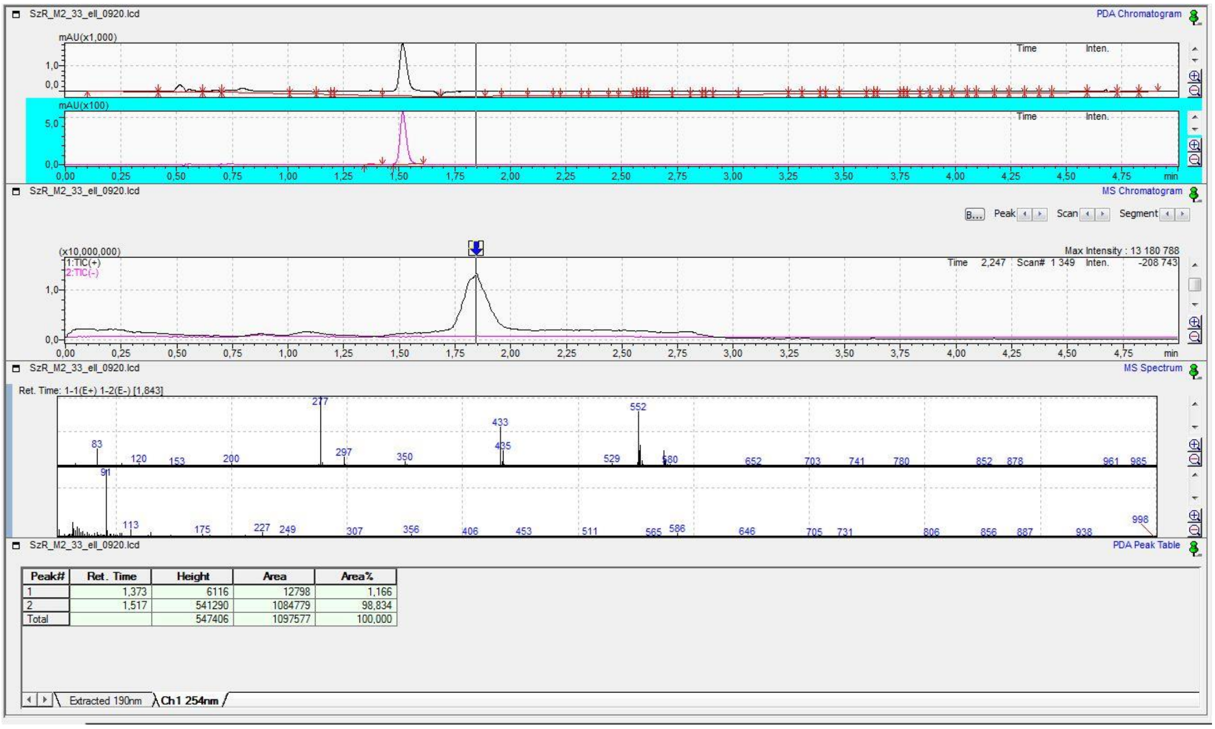

Peak#	Ret. Time	Height	Area	Area%
1	1,373	6116	12798	1,166
2	1,517	541290	1084779	98,834
Total		547406	1097577	100,000

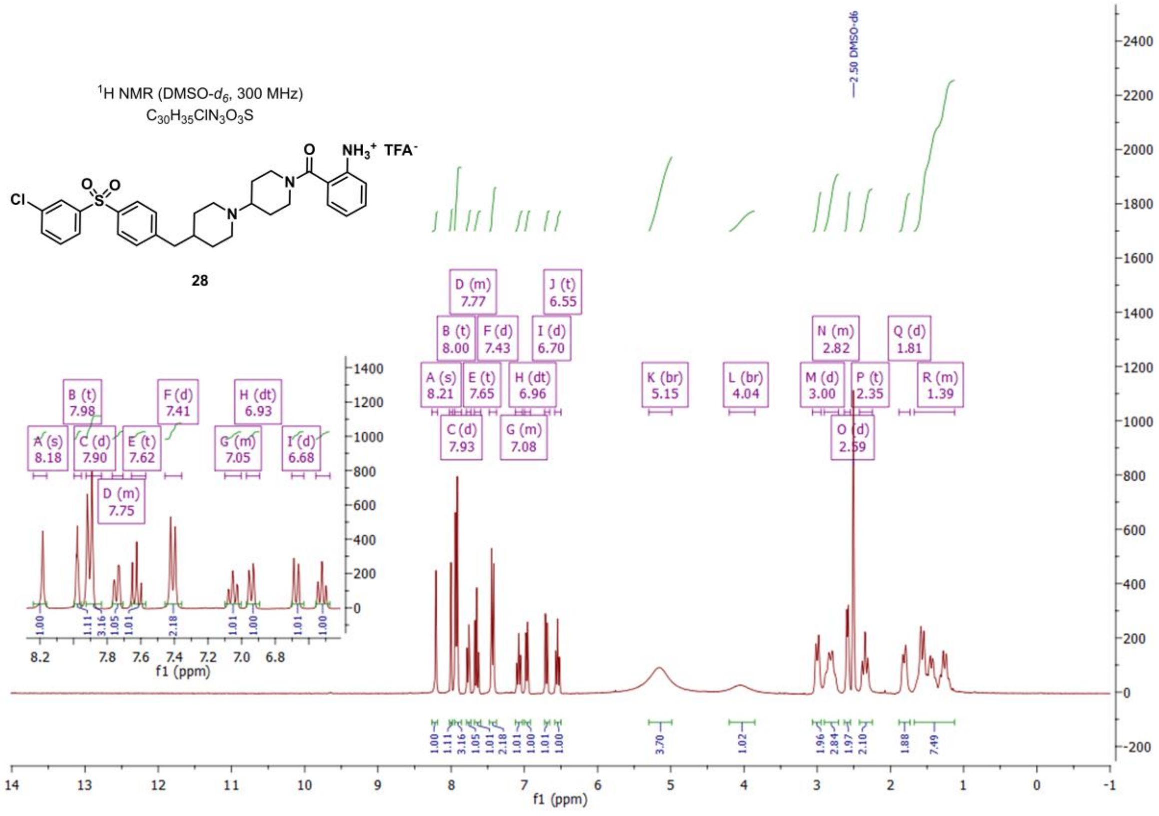

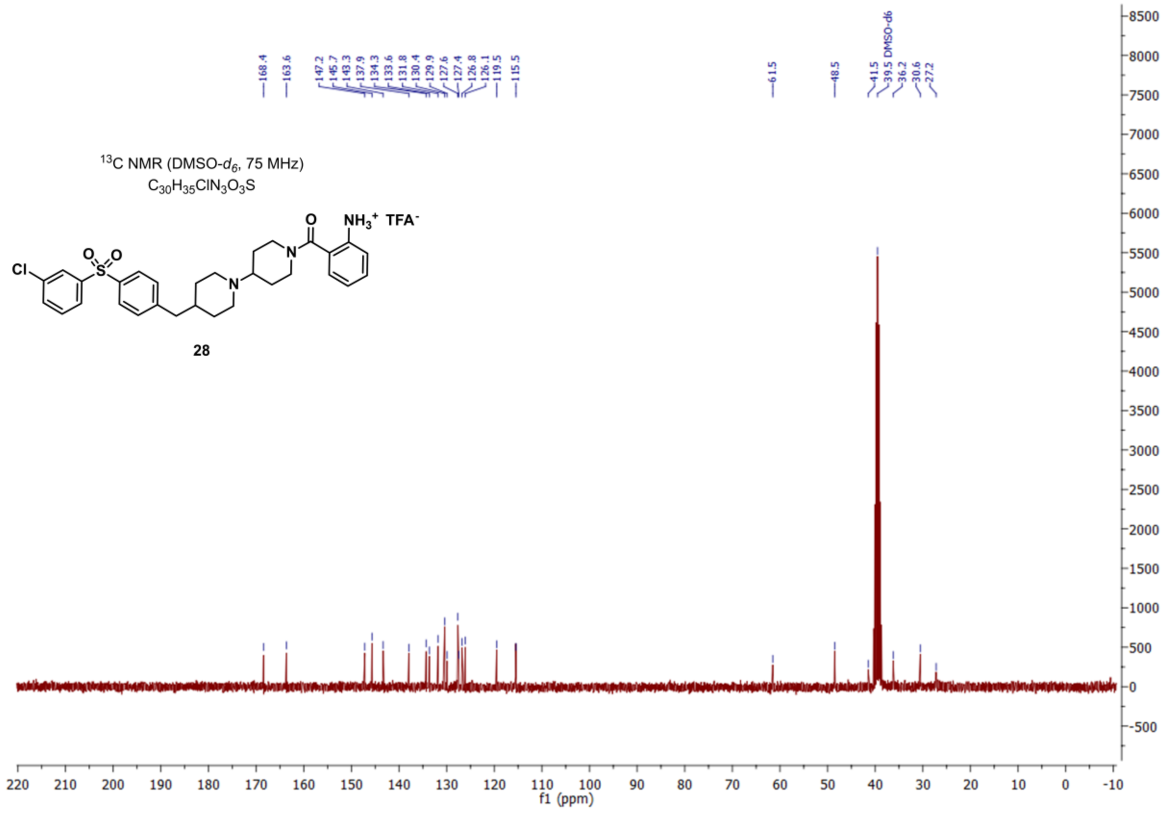

***N*-(2-(2-(2-((2-(4-(4-((3-Chlorophenyl)sulfonyl)benzyl)-[1,4'-bipiperidine]-1'-carbonyl)-phenyl)amino)ethoxy)ethoxy)ethyl)pivalamide (29)**

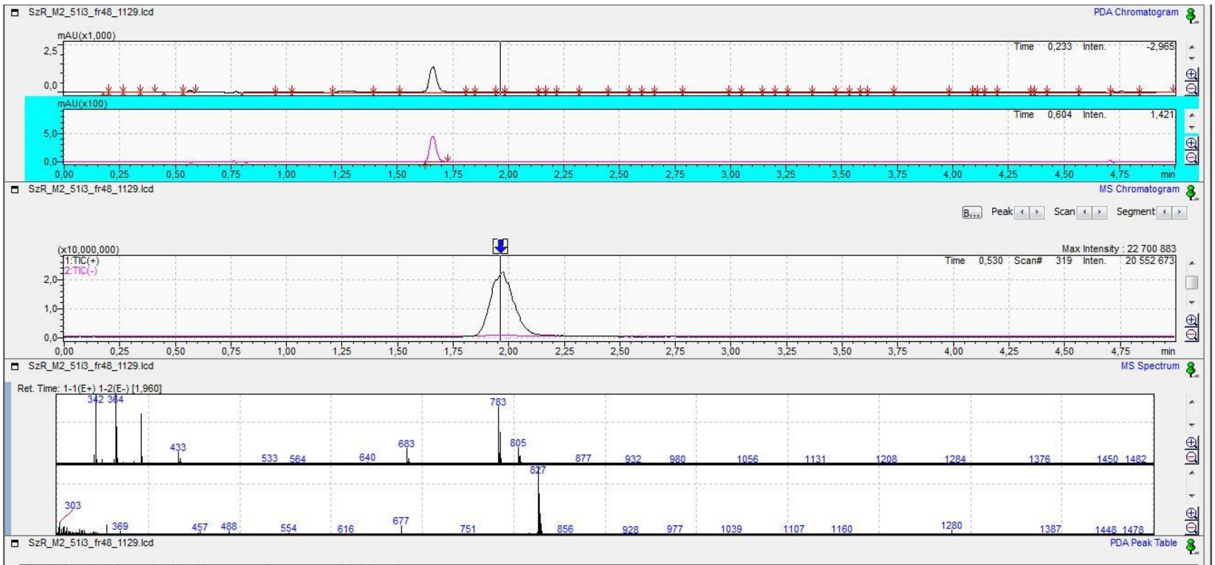

Peak#	Ret. Time	Height	Area	Area%
1	1,660	442700	907427	100,000
Total		442700	907427	100,000

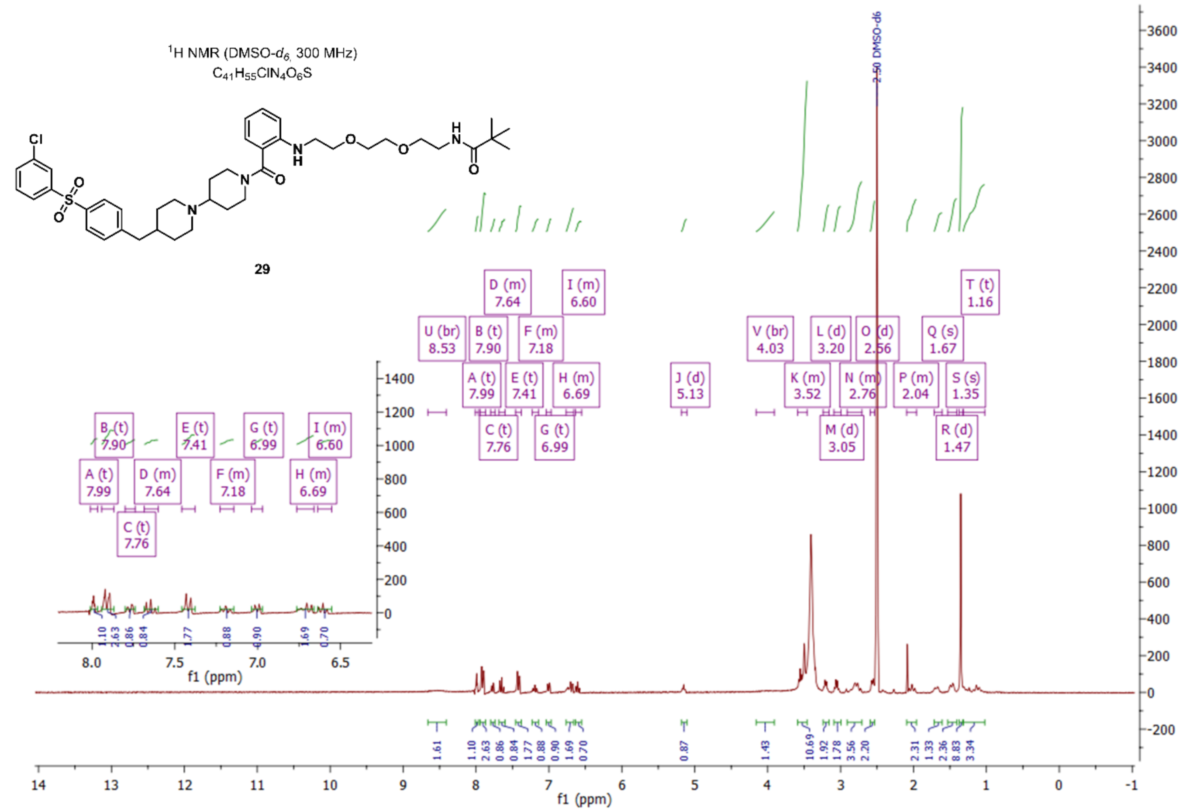

1H NMR (DMSO-d6, 300 MHz)
C41H55ClN4O6S
29
2.50 DMSO-d6
f1 (ppm)

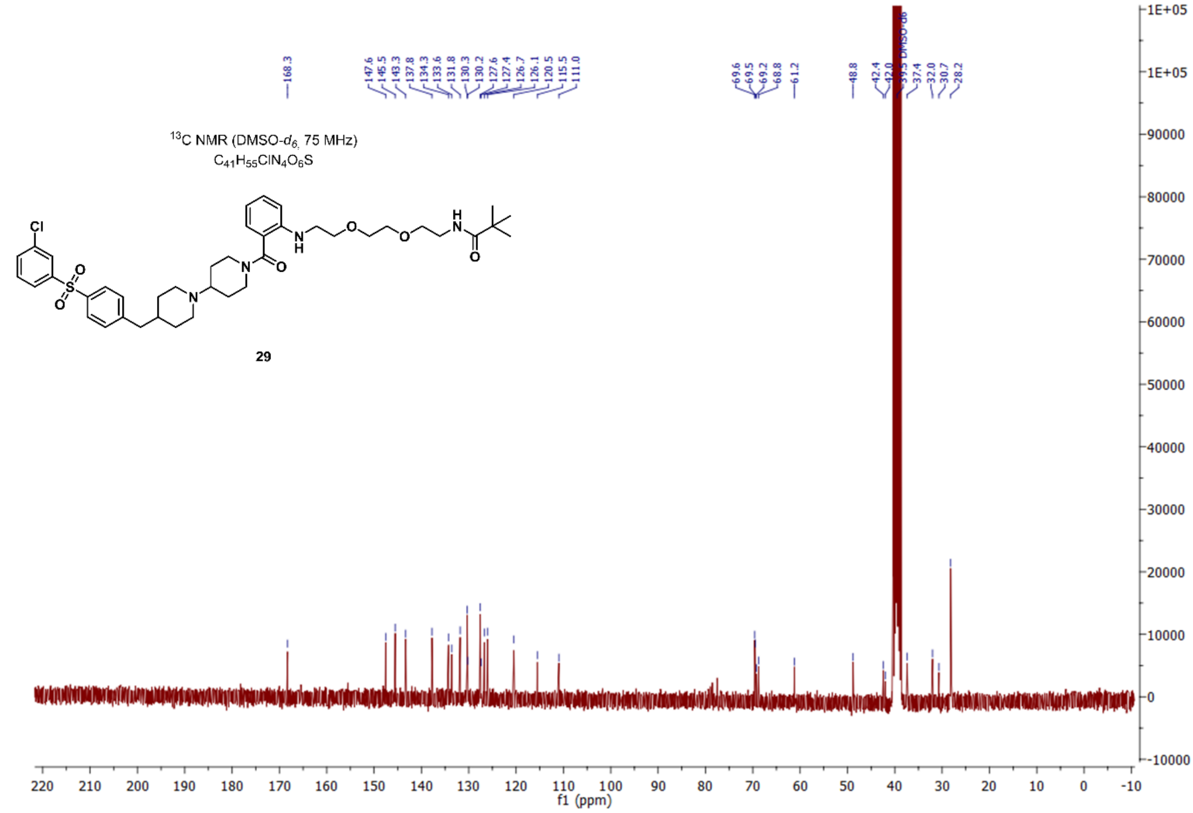

13C NMR (DMSO-d6, 75 MHz)
C41H55ClN4O6S
29
f1 (ppm)

(2-((2-(2-(2-Aminoethoxy)ethoxy)ethyl)amino)phenyl)(4-(4-((3-chlorophenyl)sulfonyl)-benzyl)-[1,4'-bipiperidin]-1'-yl)methanone TFA salt (30)

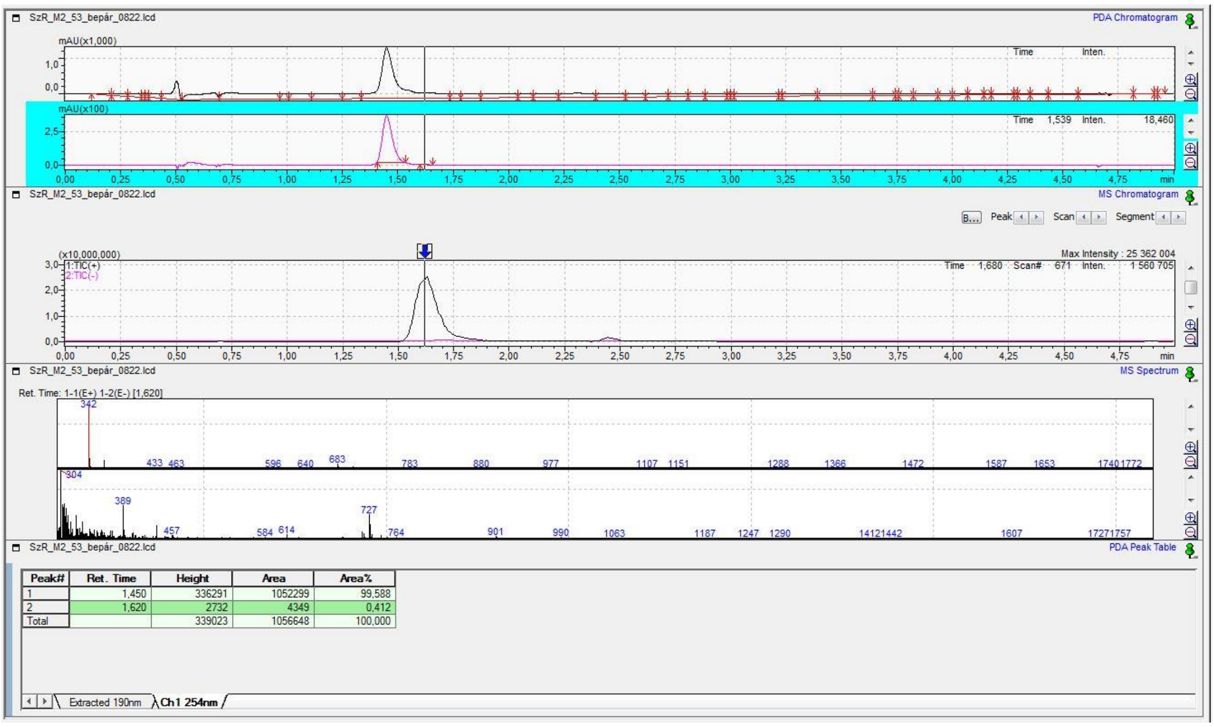

Peak#	Ret. Time	Height	Area	Area%
1	1,450	336291	1052299	99,588
2	1,620	2732	4349	0,412
Total		339023	1056648	100,000

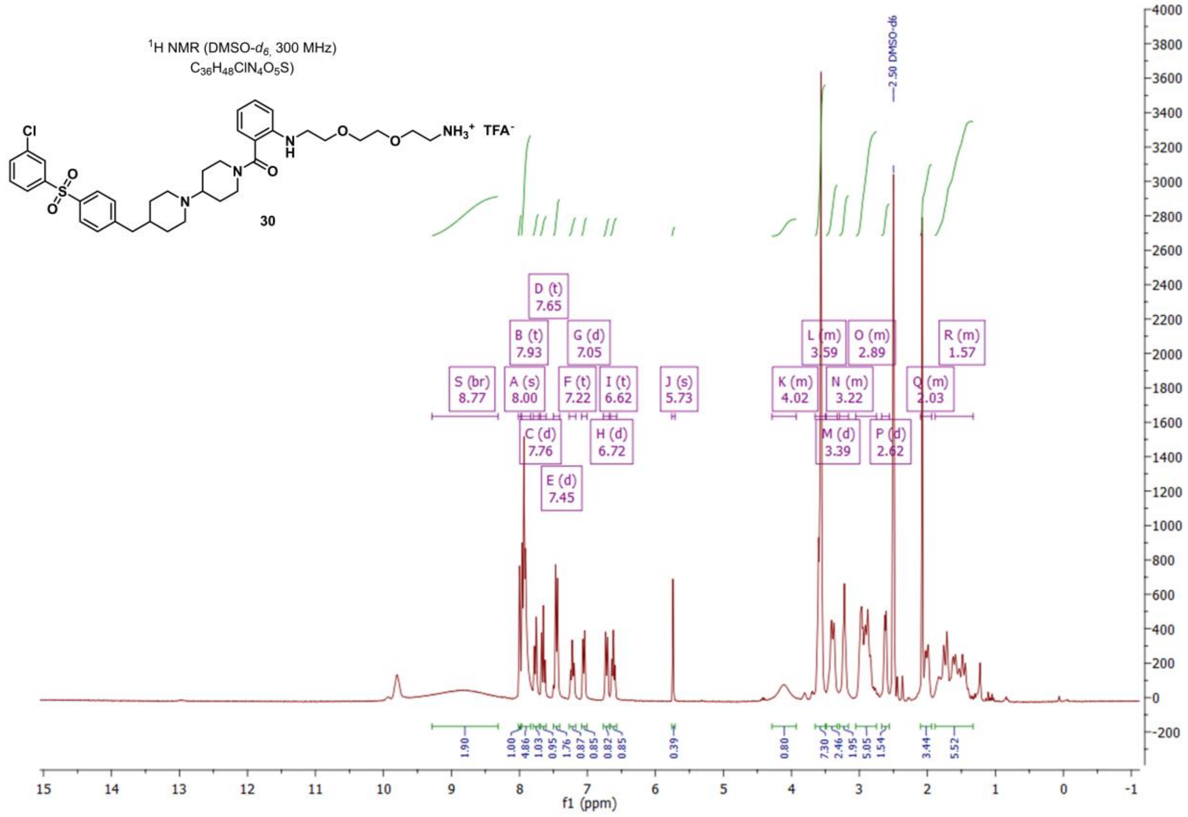

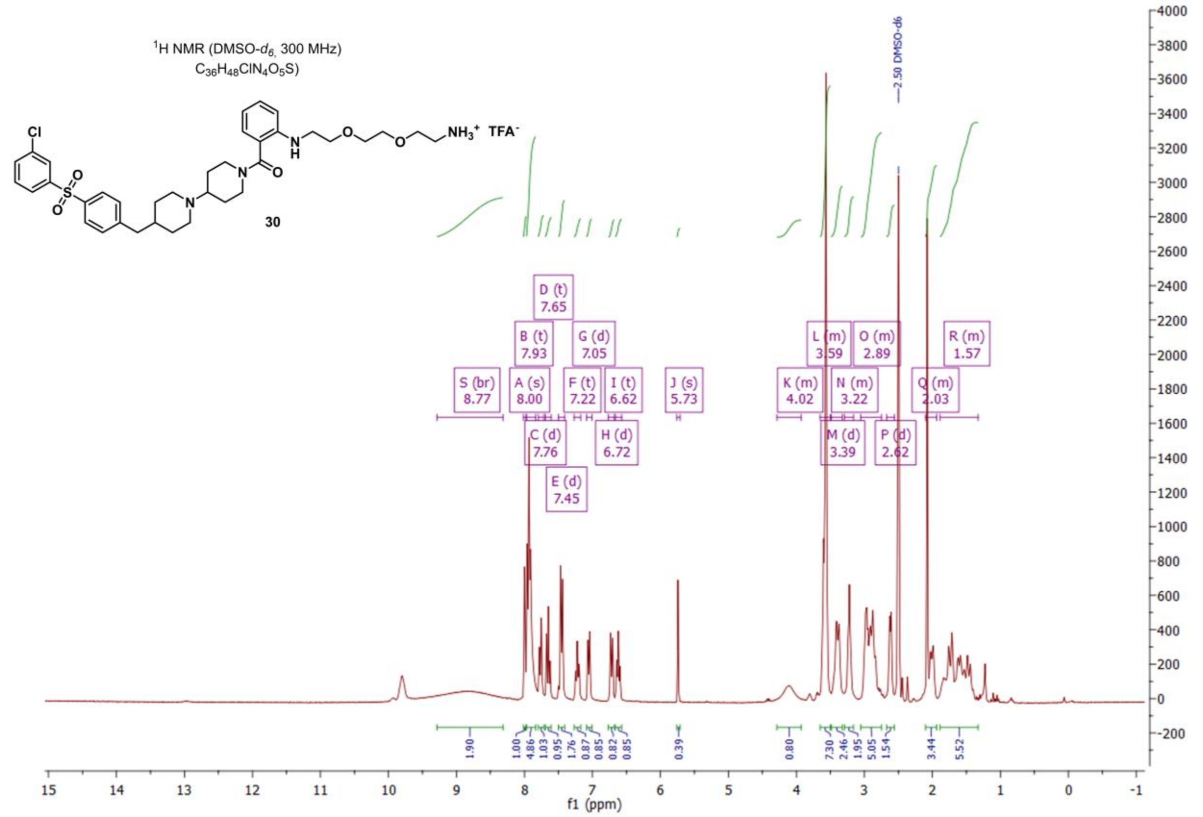

***tert*-Butyl-(1-((2-(4-(4-((3-chlorophenyl)sulfonyl)benzyl)-[1,4'-bipiperidine]-1'-carbonyl)-phenyl)amino)-10-oxo-3,6,13,16-tetraoxa-9-azaoctadecan-18-yl)carbamate** (31)

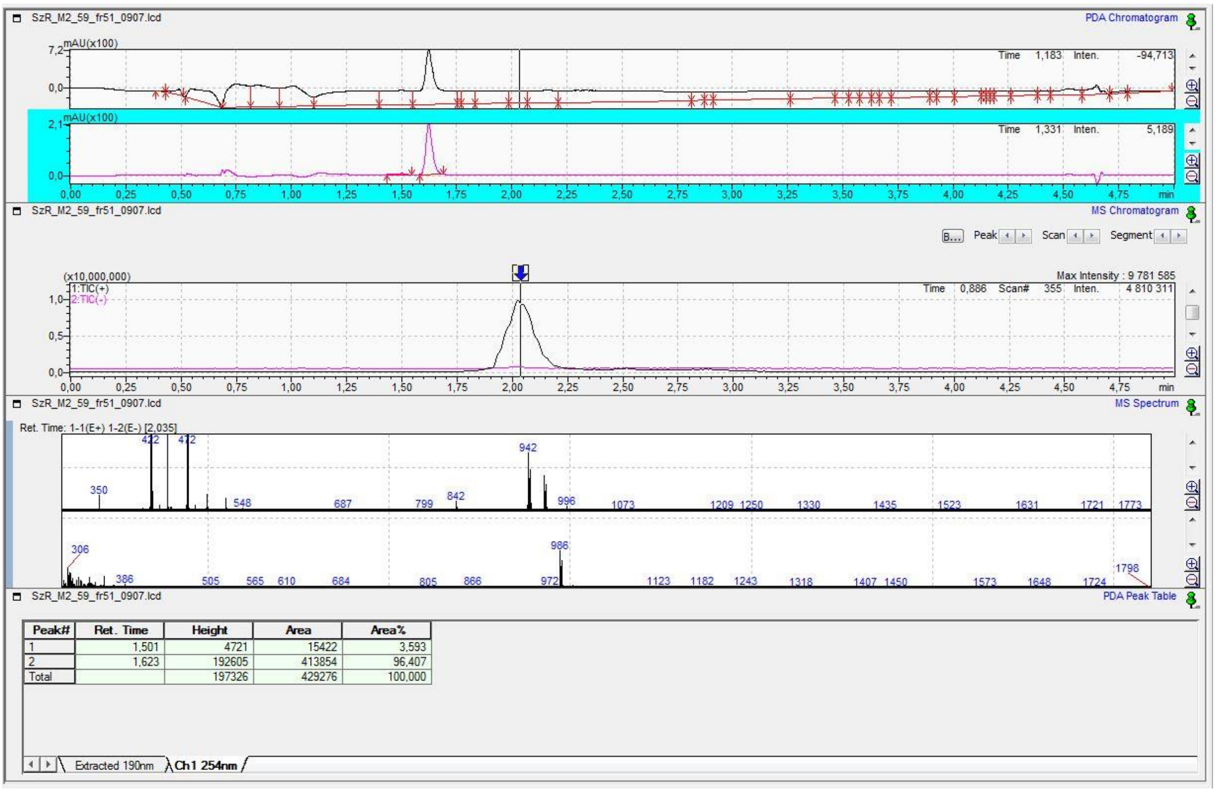

Peak#	Ret. Time	Height	Area	Area%
1	1,501	4721	15422	3,593
2	1,623	192605	413854	96,407
Total		197326	429276	100,000

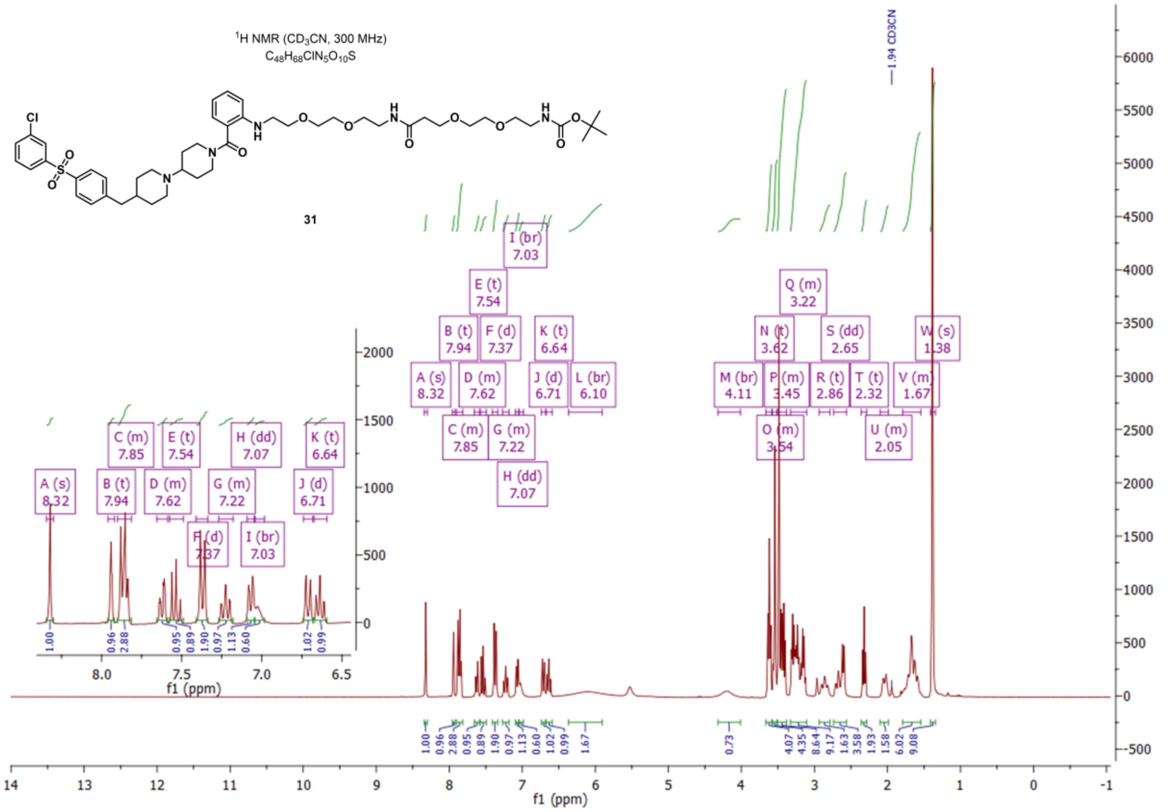

^1H NMR (CD_3CN, 300 MHz)
$C_{48}H_{68}ClN_5O_{10}S$
31
f1 (ppm)

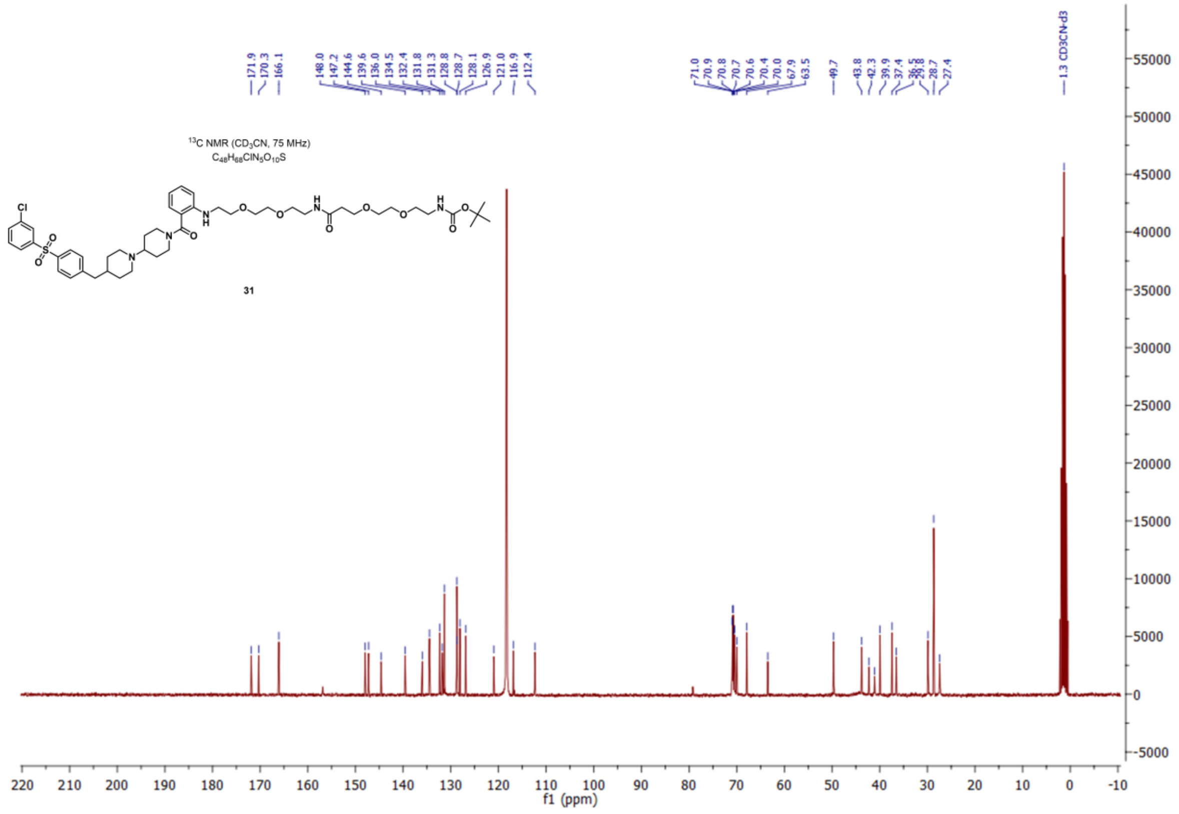

^{13}C NMR (CD_3CN, 75 MHz)
$C_{48}H_{68}ClN_5O_{10}S$
31
f1 (ppm)

3-(2-(2-Aminoethoxy)ethoxy)-N-(2-(2-(2-((2-(4-(4-((3-chlorophenyl)sulfonyl)benzyl)-[1,4'-bipiperidine]-1'-carbonyl)phenyl)amino)ethoxy)ethoxy)ethyl)propenamide TFA salt (32)

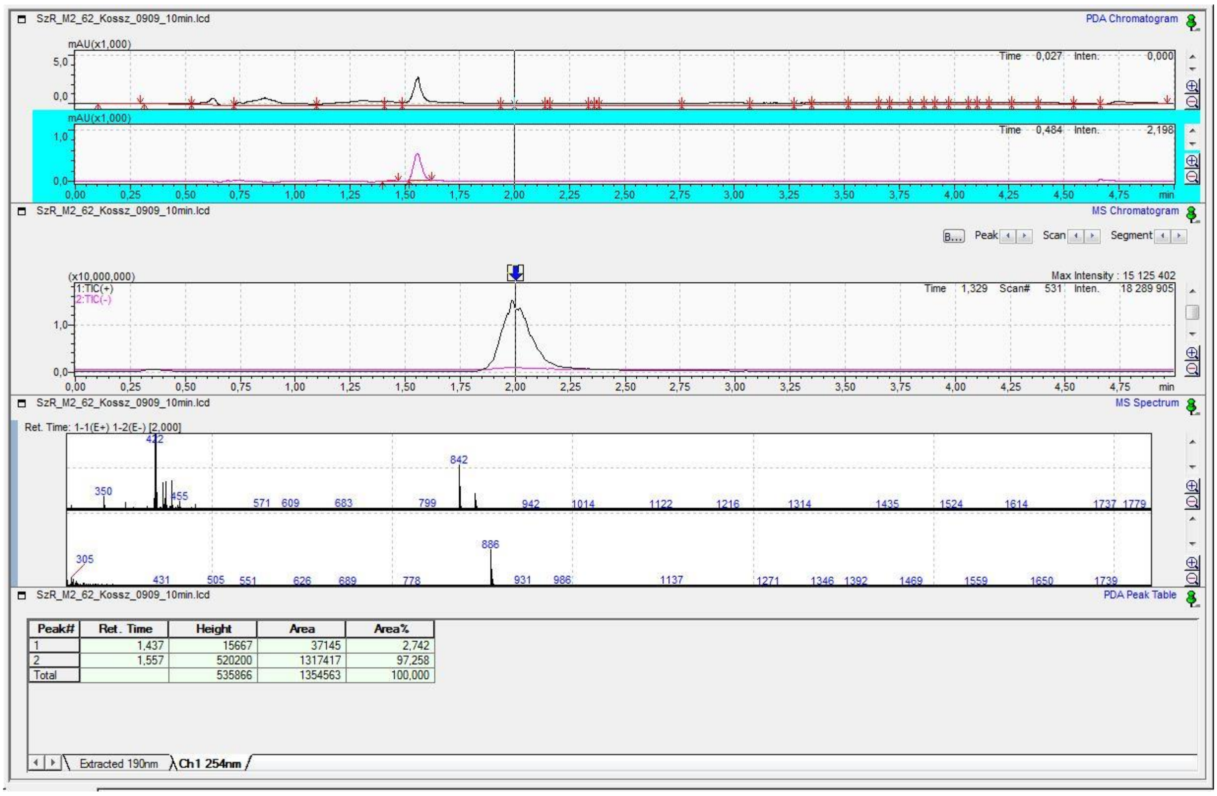

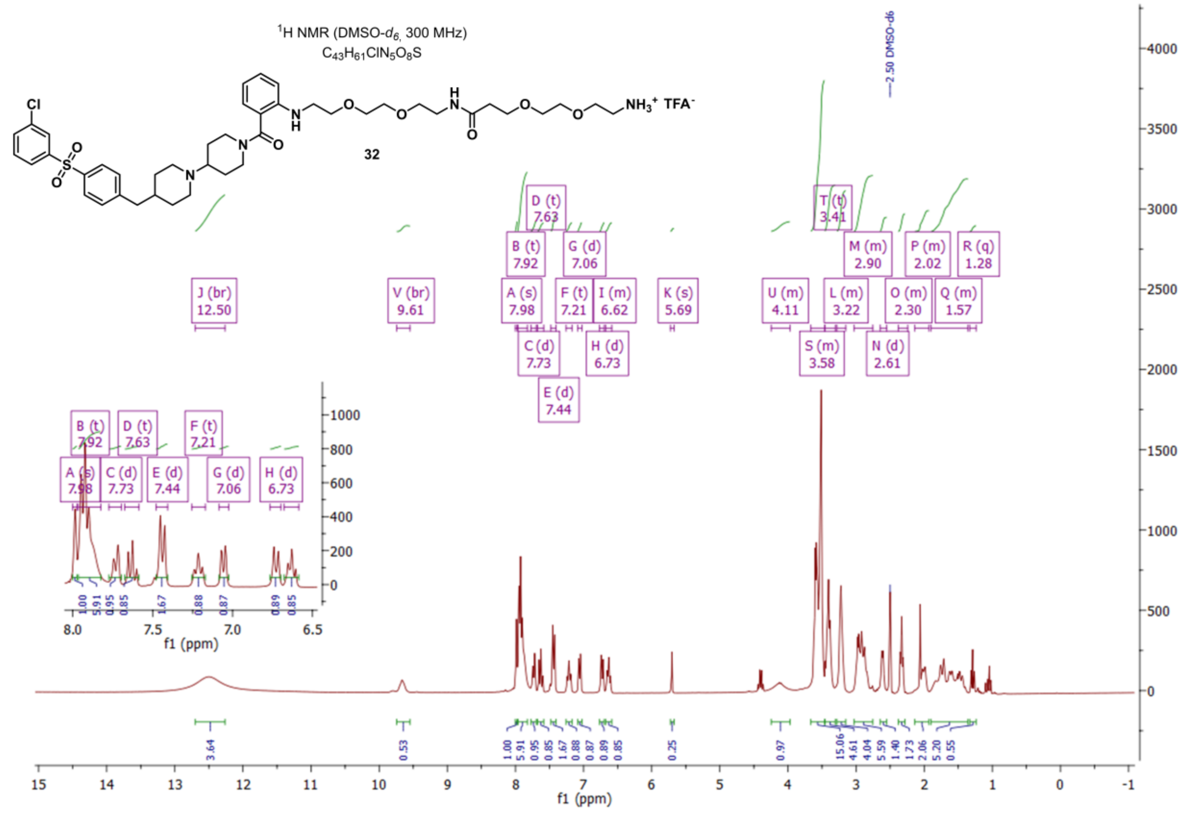

^{1}H NMR (DMSO-d_6, 300 MHz)
$C_{43}H_{61}ClN_5O_8S$
32
2.50 DMSO-d6
J (br) 12.50
V (br) 9.61
A (s) 7.98
B (t) 7.92
C (d) 7.73
D (t) 7.63
E (d) 7.44
F (t) 7.21
G (d) 7.06
H (d) 6.73
I (m) 6.62
K (s) 5.69
U (m) 4.11
S (m) 3.58
T (t) 3.41
L (m) 3.22
M (m) 2.90
N (d) 2.61
O (m) 2.30
P (m) 2.02
Q (m) 1.57
R (q) 1.28
f1 (ppm)

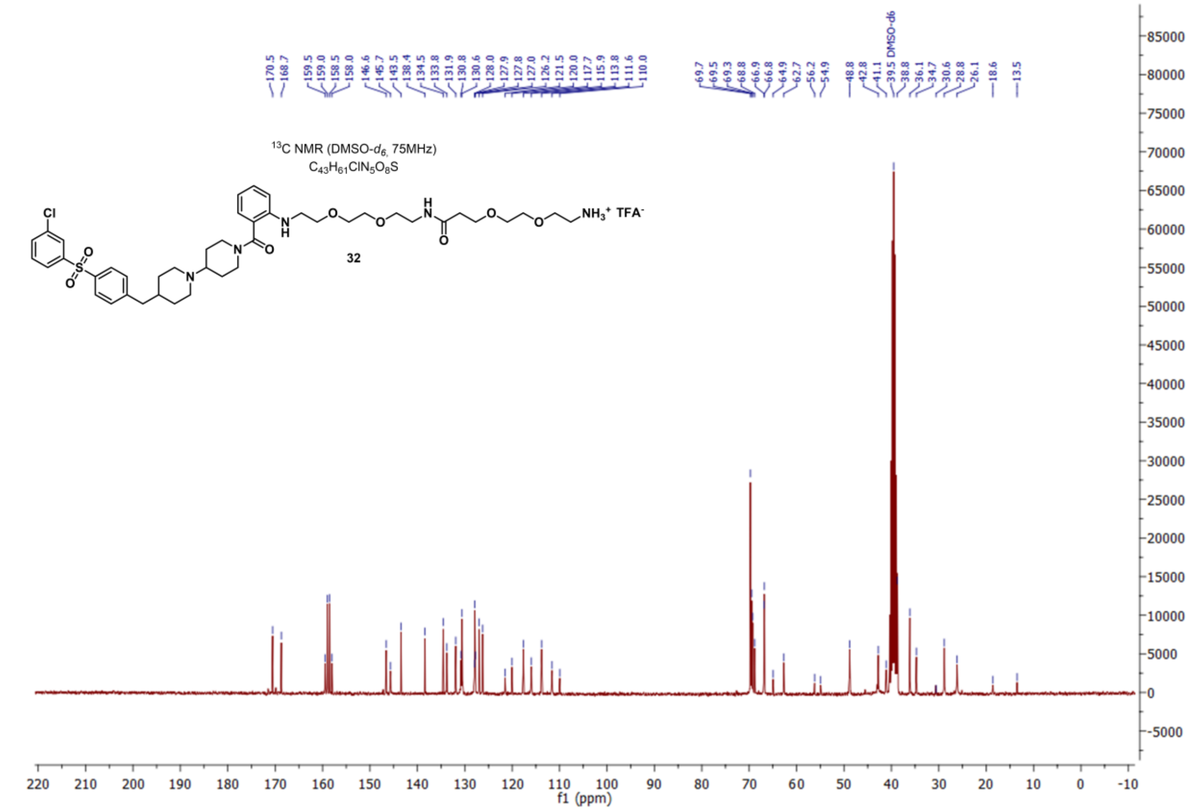

^{13}C NMR (DMSO-d_6, 75MHz)
$C_{43}H_{61}ClN_5O_8S$
32
170.5 168.7 159.5 159.0 158.5 158.0 146.6 145.7 143.5 138.4 134.5 133.8 131.9 130.8 130.6 128.0 127.9 127.8 127.0 126.2 121.5 120.0 117.7 115.9 113.8 111.6 110.0
69.7 69.5 69.3 68.8 66.9 66.8 64.9 62.7 56.2 54.9 48.8 42.8 41.1 39.5 DMSO-d6 38.8 36.1 34.7 30.6 28.8 26.1 18.6 13.5
f1 (ppm)

***N*-(1-((2-(4-(4-((3-Chlorophenyl)sulfonyl)benzyl)-[1,4'-bipiperidine]-1'-carbonyl)-phenyl)amino)-10-oxo-3,6,13,16-tetraoxa-9-azaoctadecan-18-yl)-2',7'-difluoro-3',6'-dihydroxy-3-oxo-3*H*-spiro[isobenzofuran-1,9'-xanthene]-5-carboxamide** (33)

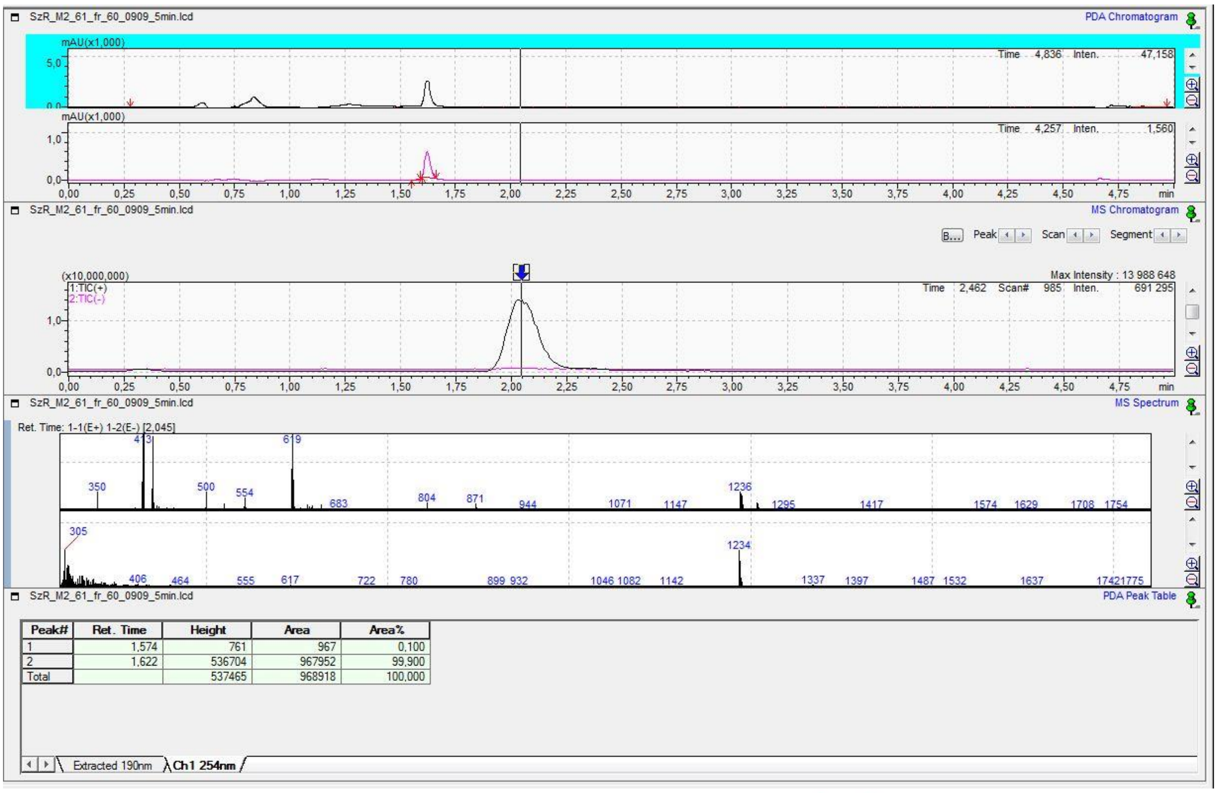

Peak#	Ret. Time	Height	Area	Area%
1	1,574	761	967	0,100
2	1,622	536704	967952	99,900
Total		537465	968918	100,000

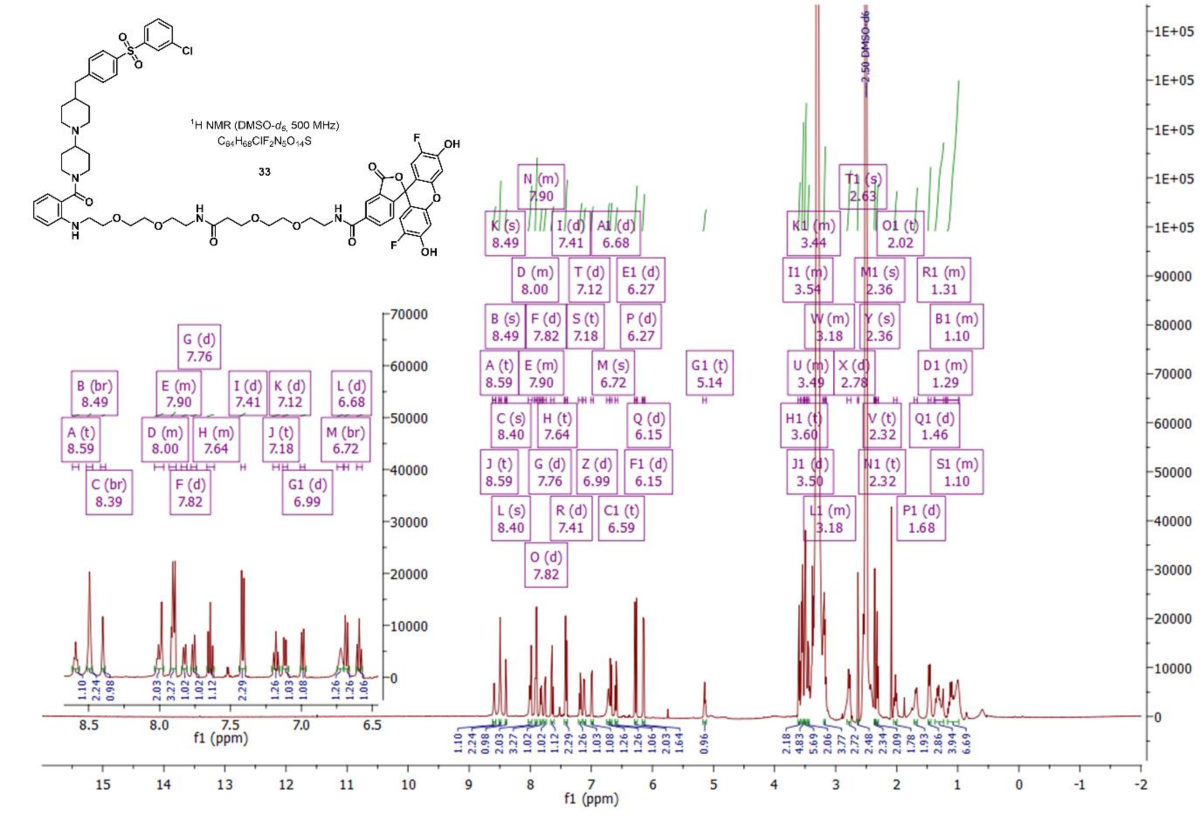

Notes and references

(1) Zeng, L.; Fu, H.; Qiao, R.; Jiang, Y.; Zhao, Y. Efficient Copper-Catalyzed Synthesis of N-Alkylanthranilic Acids via an Ortho-Substituent Effect of the Carboxyl Group of 2-Halobenzoic Acids at Room Temperature. *Adv. Synth. Catal.* **2009**, *351* (10), 1671–1676. https://doi.org/10.1002/adsc.200900065.

(2) Suno, R.; Lee, S.; Maeda, S.; Yasuda, S.; Yamashita, K.; Hirata, K.; Horita, S.; Tawaramoto, M. S.; Tsujimoto, H.; Murata, T.; Kinoshita, M.; Yamamoto, M.; Kobilka, B. K.; Vaidehi, N.; Iwata, S.; Kobayashi, T. Structural Insights into the Subtype-Selective Antagonist Binding to the M2 Muscarinic Receptor. *Nat. Chem. Biol.* **2018**, *14* (12), 1150–1158. https://doi.org/10.1038/s41589-018-0152-y.

(3) Madhavi Sastry, G.; Adzhigirey, M.; Day, T.; Annabhimoju, R.; Sherman, W. Protein and Ligand Preparation: Parameters, Protocols, and Influence on Virtual Screening Enrichments. *J. Comput. Aided. Mol. Des.* **2013**, *27* (3), 221–234. https://doi.org/10.1007/s10822-013-9644-8.

(4) Lu, C.; Wu, C.; Ghoreishi, D.; Chen, W.; Wang, L.; Damm, W.; Ross, G. A.; Dahlgren, M. K.; Russell, E.; Von Bargen, C. D.; Abel, R.; Friesner, R. A.; Harder, E. D. OPLS4: Improving Force Field Accuracy on Challenging Regimes of Chemical Space. *J. Chem. Theory Comput.* **2021**, *17* (7), 4291–4300. https://doi.org/10.1021/acs.jctc.1c00302.

(5) Fusani, L.; Palmer, D. S.; Somers, D. O.; Wall, I. D. Exploring Ligand Stability in Protein Crystal Structures Using Binding Pose Metadynamics. *J. Chem. Inf. Model.* **2020**, *60* (3), 1528–1539. https://doi.org/10.1021/acs.jcim.9b00843.

(6) Friesner, R. A.; Banks, J. L.; Murphy, R. B.; Halgren, T. A.; Klicic, J. J.; Mainz, D. T.; Repasky, M. P.; Knoll, E. H.; Shelley, M.; Perry, J. K.; Shaw, D. E.; Francis, P.; Shenkin, P. S. Glide: A New Approach for Rapid, Accurate Docking and Scoring. 1. Method and Assessment of Docking Accuracy. *J. Med. Chem.* **2004**, *47* (7), 1739–1749. https://doi.org/10.1021/jm0306430.

(7) Sun, W. C.; Gee, K. R.; Klaubert, D. H.; Haugland, R. P. Synthesis of Fluorinated Fluoresceins. *J. Org. Chem.* **1997**, *62* (19), 6469–6475. https://doi.org/10.1021/jo9706178.

(8) Tóth, A. D.; Garger, D.; Prokop, S.; Soltész-Katona, E.; Várnai, P.; Balla, A.; Turu, G.;

Hunyady, L. A General Method for Quantifying Ligand Binding to Unmodified Receptors Using Gaussia Luciferase. *J. Biol. Chem.* **2021**, *296*, 100366. https://doi.org/10.1016/j.jbc.2021.100366.

(9) Olsen, R. H. J.; DiBerto, J. F.; English, J. G.; Glaudin, A. M.; Krumm, B. E.; Slocum, S. T.; Che, T.; Gavin, A. C.; McCorvy, J. D.; Roth, B. L.; Strachan, R. T. TRUPATH, an Open-Source Biosensor Platform for Interrogating the GPCR Transducerome. *Nat. Chem. Biol.* **2020**, *16* (8), 841–849. https://doi.org/10.1038/s41589-020-0535-8.

(10) Kroeze, W. K.; Sassano, M. F.; Huang, X. P.; Lansu, K.; McCorvy, J. D.; Giguère, P. M.; Sciaky, N.; Roth, B. L. PRESTO-Tango as an Open-Source Resource for Interrogation of the Druggable Human GPCRome. *Nat. Struct. Mol. Biol.* **2015**, *22* (5), 362–369. https://doi.org/10.1038/nsmb.3014.

(11) András D. Tóth, Bence Szalai, Orsolya T. Kovács, Dániel Garger, Susanne Prokop, Eszter Soltész-Katona, András Balla, Asuka Inoue, Péter Várnai, Gábor Turu, L. H. G Protein–Coupled Receptor Endocytosis Generates Spatiotemporal Bias in β-Arrestin Signaling. *Sci. Signal.* **2024**, *17*, eadi0934. https://doi.org/10.1126/scisignal.adi0934.

(12) Vice, S.; Bara, T.; Bauer, A.; Evans, C. A.; Ford, J.; Josien, H.; Mccombie, S.; Miller, M.; Nazareno, D.; Palani, A.; Tagat, J. Concise Formation of 4-Benzyl Piperidines and Related Derivatives Using a Suzuki Protocol. *J. Org. Chem.* **2001**, *66*, 2487–2492. https://doi.org/10.1021/jo0007682.

(13) Suzuki, T.; Hisakawa, S.; Itoh, Y.; Suzuki, N.; Takahashi, K.; Kawahata, M.; Yamaguchi, K.; Nakagawa, H.; Miyata, N. Design, Synthesis, and Biological Activity of Folate Receptor-Targeted Prodrugs of Thiolate Histone Deacetylase Inhibitors. *Bioorg. Med. Chem. Lett.* **2007**, *17* (15), 4208–4212. https://doi.org/10.1016/j.bmcl.2007.05.040.
